# Sterically protected π-electron systems for efficient solid-state photon upconversion

**DOI:** 10.1038/s41467-026-73898-0

**Published:** 2026-06-23

**Authors:** Naoyuki Harada, Hayato Shoyama, Nutnicha Boonmong, Kiichi Mizukami, Yuya Watanabe, Pei Zhao, Masahiro Ehara, Yoichi Sasaki, Nobuo Kimizuka

**Affiliations:** 1 Department of Applied Chemistry, Graduate School of Engineering, Kyushu University, 744 Moto-oka, Nishi-ku, Fukuoka, Japan; 2 Research Center for Computational Science, Institute for Molecular Science, SOKENDAI, 38 Nishigo-Naka, Myodaiji, Okazaki, Japan; 3 Center for Molecular Systems (CMS), Kyushu University, 744 Moto-oka, Nishi-ku, Fukuoka, Japan; 4 Research Center for Negative Emissions Technologies (K-NETs), Kyushu University, 744 Moto-oka, Nishi-ku, Fukuoka, Japan

**Keywords:** Light harvesting, Materials for energy and catalysis, Excited states

## Abstract

A solid-state visible-to-ultraviolet triplet–triplet annihilation-based photon upconversion system driven by low-intensity light at sunlight levels is developed. Realizing such a solid-state photon upconversion system has been challenging because it is difficult to achieve high fluorescence quantum yield and fast triplet exciton diffusion simultaneously, which requires methodologies to precisely control interactions among chromophores and suppress quenching of both singlet and triplet excited states. Here, we report that a group of dihydroindeno[2,1-*a*]indene derivatives functionalized with alkyl chains above and below the π-plane satisfies all these requirements. Among three derivatives investigated, we identify the optimal emitter structure that exhibits the highest photon upconversion quantum yield in both solution and crystalline states. The solid-state system is less affected by crystalline defects, exhibiting high photoluminescence quantum yield, long triplet lifetime, and fast triplet diffusion, with an absolute photon upconversion quantum yield of 1.9% and a low threshold excitation intensity of 1.2 mW cm^−2^.

## Abstract (web summary)

A solid-state visible-to-ultraviolet upconverting system is developed using suitably alkyl-functionalized acceptors. Reconciling fast triplet energy diffusion and high luminescence intensity enable 1.9% conversion at solar-level irradiance (1.2 mW cm⁻²).

## Introduction

**Fig. 1:**
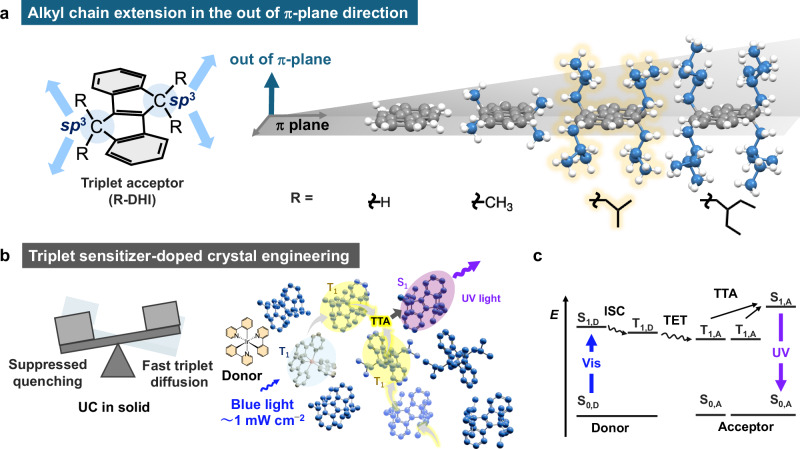
Engineering of intermolecular interactions for efficient solid-state photon upconversion. **a** Controlled intermolecular interaction by extending alkyl chains from the *sp*^3^ carbon of dihydroindeno[2,1-*a*]indene (DHI) derivatives in the out-of-π-plane direction. **b** Crystal engineering for highly efficient solid TTA-UC systems that simultaneously achieve suppression of the quenching and fast triplet diffusion. **c** An energy diagram of the Vis-to-UV TTA-UC.

The development of technologies for effectively harnessing sunlight is essential to the sustainable development of modern society. Although inorganic photocatalysts using ultraviolet (UV) light have achieved high-efficiency photocatalytic water splitting^1^, they suffer from the low UV fraction in sunlight (about 3% for the 300–400 nm range)^2,3^. To solve this issue, there is a thirst for developing high-performance solid-state upconversion materials that convert visible (Vis) light into UV light. Triplet-fusion, i.e., triplet–triplet annihilation-based photon upconversion (TTA-UC), has attracted much attention as its potential operation under excitation light close to solar irradiance (~1 mW cm^–2^) in deaerated solutions, attaining a high quantum yield exceeding 10%^4–16^. The TTA-UC process is initiated by light absorption by the donor, followed by intersystem crossing to form excited triplets, which are then transferred to the acceptor (triplet energy transfer, TET) through the Dexter mechanism. Subsequently, the two acceptors in the excited triplet state diffuse and collide in solution to form a singlet with a certain spin-statistical probability^17^, and upconverted light is emitted from the acceptor in the excited singlet state (Fig. 1c).

To date, efficient TTA-UC systems that convert visible light into UV light have been demonstrated exclusively by exploiting molecular diffusion in solution. By using acceptors such as 1,4-bis((triisopropylsilyl)ethynyl)naphthalene (TIPS-Nph) and 2,5-diphenyloxazole (PPO), UC quantum yields (*Φ*_UC_) of up to 16.4% have been achieved^18^. These systems have been applied to blue-light-induced photocatalytic water splitting and photoreactions in solution^18–23^. However, solvent volatility poses a critical limitation for device applications and long-term use. There are attempts to develop rigid polymer films containing liquid droplets of dissolved donors and acceptors. Despite exhibiting moderate self-standing properties and a high *Φ*_UC_^24,25^, these systems are highly susceptible to liquid leakage, and molecular diffusion limits the rate constants of the donor-to-acceptor TET and TTA.

To achieve the practical application of TTA-UC, it is essential to realize efficient upconversion in the solvent-free solid state under low excitation intensity. The highly ordered self-assembly of TTA-UC chromophores may enable more efficient TET and faster triplet energy migration processes than in solution. Other advantages of self-assembled TTA-UC include demonstrated oxygen tolerance^26–28^. In spite of these demands, there are few reports on the Vis-to-UV TTA-UC in neat solid systems^29–33^, and an unresolved issue remains: the *Φ*_UC_ in solid systems is considerably lower than in the solution systems. The significant reduction in UC performance in solids is due to the quenching of singlet and triplet excitons at nonradiative deactivation sites. The resultant nonradiative deactivation significantly reduces fluorescence quantum yield (*Φ*_F_) and triplet lifetime (*τ*_T_) compared to the molecularly dispersed solution state^34–36^. In addition, segregation of donors occurs in host acceptor crystals, significantly impairing the performance of TTA-UC^29,37–39^. Even polycrystalline films of PPO formed by a temperature-gradient solidification method required one to two orders of magnitude higher excitation intensity for TTA to occur in the solid state dominantly^29^. Thus, molecular design guidelines that enable uniform dispersion of the donors within the crystalline molecular assembly without phase separation, while demonstrating high quantum yield unaffected by the formation of crystalline defects, have been desired.

To circumvent the quenching of singlet and triplet excitons in crystals and enhance fluorescence quantum yield, it would be effective to introduce appropriate bulky substituents into acceptors. This modification prevents close contact between acceptors’ π-conjugated backbones within crystals, thereby reducing the number of exciton-quenching sites. In addition, to ensure miscibility with the donor, substituents introduced into the acceptors should be designed to have a large surface area so that the magnitude of the van der Waals forces, the source of the cohesive force of the acceptor molecule, can be adjusted.

Conventionally, methyl, phenyl, and *tert*-butyl groups have been introduced to highly emissive π-systems such as acenes and rylenes to prevent singlet quenching in the solid state^36,40–43^. In the case of triplets, it has been demonstrated that the separation of the chromophores extends the lifetime of the excited state^44^, but excessive separation of π-systems hampers energy migration in solids as efficient triplet energy transfer requires π-orbital overlap. Therefore, the interchromophore distance and molecular orientation of the acceptors must be optimized to achieve efficient solid-state TTA-UC that reconciles the conflicting properties of high fluorescence quantum yield, long triplet lifetime, and fast triplet energy diffusion.

Here, we achieve efficient TTA-UC in both solution and solid state by controlling intermolecular electronic interactions through alkyl-chain substitution at the *sp*^3^ carbons of the highly emissive 5,10-dihydroindeno[2,1-*a*]indene (DHI) skeleton (Fig. 1a). Thanks to the inherently non-planar tetrahedral geometry of *sp*^3^ carbon, a steric hindrance that spatially protects above and below the π-plane is straightforwardly introduced to DHI via one-pot synthesis. This acceptor design enables controlled intermolecular interactions in the crystalline state, leading to fast diffusion of triplet energy while suppressing singlet and triplet quenching (Fig. 1b); the DHI derivatives show defect-tolerant fluorescence quantum yields. A film using an isobutyl-substituted DHI (*i*Bu-DHI) acceptor and triplet donor Ir(ppy)_3_ shows a high *Φ*_UC_ of 1.9% and the low threshold excitation intensity (*I*_th_) of 1.2 mW cm^−2^ which is lower than the solar irradiance near the excitation wavelength (1.4 mW cm^–2^, 445 nm ± 5 nm)^45^. Furthermore, dense molecular assembly of the solid system provides oxygen-tolerant UC emission. Together with theoretically calculated TET and TTA reaction times for molecular pairs found in crystals, we discuss possible factors contributing to the superior UC performances of the *i*Bu-DHI/Ir(ppy)_3_ film.

## Results

**Fig. 2:**
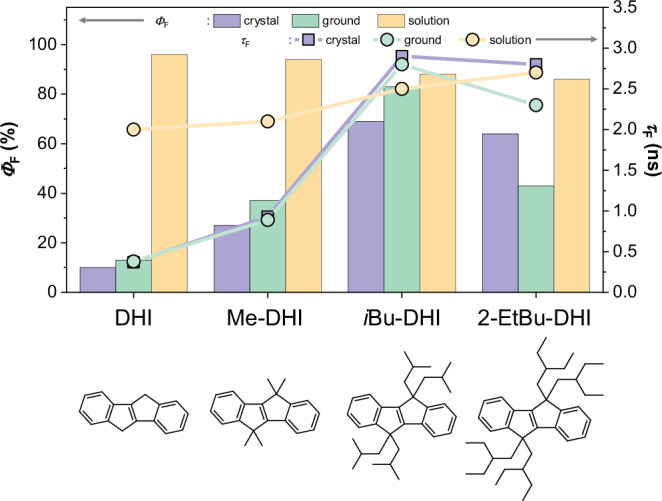
Photophysical properties of DHI derivatives. Fluorescence quantum yields (*Φ*_F_) and fluorescence lifetimes (*τ*_F_) of DHI, Me-DHI, *i*Bu-DHI, and 2-EtBu-DHI in the crystalline solid (purple), in the ground powder (green), and in the solution state (yellow; 200 μM in deaerated THF). Chemical structures of DHI, Me-DHI, *i*Bu-DHI, and 2-EtBu-DHI are also shown at the bottom.

DFT and TD-DFT calculations confirmed that introducing alkyl chains into DHI chromophores slightly reduces the lowest excited singlet (S_1_) and triplet (T_1_) energy levels, and spatial distributions of unpaired electron density in the T_1_ state were similar, as shown in Figures S5 and S6, respectively. Figure S7 shows the absorption and emission spectra of Ir(ppy)_3_, DHI, and alkyl-substituted DHIs in deaerated THF solutions. The unsubstituted DHI exhibits a high fluorescence quantum yield (*Φ*_F_ = 96%) in diluted solution, but it decreases dramatically in the crystal state (*Φ*_F_ = 10%, Fig. 2, and Table 1). This phenomenon is commonly observed in dyes and is explained by the picture where nonradiative quenching sites promote the deactivation of singlet excitons^44^. Changes in molecular geometry upon crystallization can also promote intersystem crossing to the triplet state and thereby enhance deactivation^46,47^. Meanwhile, the introduction of alkyl groups on the *sp*^3^ carbons of DHI increased fluorescence quantum yield in crystals. The *i*Bu-DHI solid showed high *Φ*_F_ (69–83%) comparable to that in solution (Fig. 2, and Table 1). These results indicate that the alkyl chains introduced to the *sp*^3^ carbons protect the upper and lower surfaces of the π-orbitals and effectively reduce their overlap in the crystalline state. Consequently, the formation of detrimental quenching sites is suppressed.

**Table 1:** Fluorescence quantum yield (*Φ*_F_), fluorescence lifetime (*τ*_F_) of DHI, Me-DHI, *i*Bu-DHI, and 2-EtBu-DHI in the crystalline state before and after grinding, and in deaerated THF (200 μM)

	*Φ*_F_(%)			*τ*_F_(ns)		
	Solution	Crystal	Ground	Solution	Crystal	Ground
DHI	96	10	12	2.0	0.37	0.38
Me-DHI	94	27	37	2.1	0.93	0.89
*i*Bu-DHI	88	69	83	2.5	2.9	2.8
2-EtBu-DHI	86	64	43	2.7	2.8	2.3

We then systematically investigated the TTA-UC characteristics in deaerated solutions and experimentally determined the alkyl chain structure to minimize the threshold excitation intensity (*I*_th_), the excitation light intensity above which the TTA process becomes dominant. We employed Ir(ppy)_3_ as a donor (sensitizer) because its triplet energy level (2.4 eV)^48,49^ is slightly higher than that calculated for alkylated DHI (2.33–2.36 eV, Figure S5). The mixed solution of DHI, Me-DHI, and *i*Bu-DHI with Ir(ppy)_3_ showed upconverted UV emission peaked around 360 nm, 365 nm, and 370 nm, respectively, upon laser irradiation at 445 nm under Ar atmosphere (Figure S9). The 2-EtBu-DHI UC emission was much weaker due to aggregation (section 7 in supplementary information). The double-logarithmic plots of UC emission intensity as a function of excitation intensity showed a change in slope from 2 to 1, characteristic of TTA-UC (Figure S10a). The *Φ*_UC_ in the optimized *i*Bu-DHI systems was  10% ([*i*Bu-DHI] = 10 mM), comparable to the highest *Φ*_UC_ in the previous Vis-to-UV TTA-UC systems (Figure S11b)^50–54^. In contrast, a small *Φ*_UC_ of 3.1% was observed for unsubstituted DHI (Figure S10b, and Table S1). This low *Φ*_UC_ may be attributed to the instability under the laser excitation (Figure S13) and quenching of the excited state in the encountered dimers^55–57^. The high *Φ*_UC_ observed for *i*Bu-DHI suggests that the presence of alkyl side chains suppresses such nonradiative deactivation processes^58,59^. The involvement of the TTA process is also supported by the long-delayed emission with over-microsecond lifetimes (Figure S10c). Interestingly, the observed triplet lifetimes (*τ*_T_) depend on the length of the alkyl group. As the side chain becomes bulkier from DHI to *i*Bu-DHI, the *τ*_T_ becomes longer from 1.4 ms to 7.8 ms (Figure S10c, and Table S1). This trend is consistent with the reports that TIPS-Nph derivatives with bulkier substituents exhibit longer triplet lifetimes due to the suppression of quenching by the T_1_–S_0_ interaction^18^. The long *τ*_T_ of *i*Bu-DHI realized significantly low *I*_th_ (1.8 mW cm^−2^, Figure S11a, Table S1) close to sunlight (ca. 1.4 mW cm^−2^ at 445 nm ± 5 nm)^45^. Also, it exhibited stable TTA-UC luminescence under continuous laser irradiation in THF (Figure S12). The excellent UC performances observed, especially for *i*Bu-DHI (Table S1) are associated with controlled interchromophore interactions enabled by the alkyl chains in the DHI derivatives extended in the out-of-π-plane direction.

**Fig. 3:**
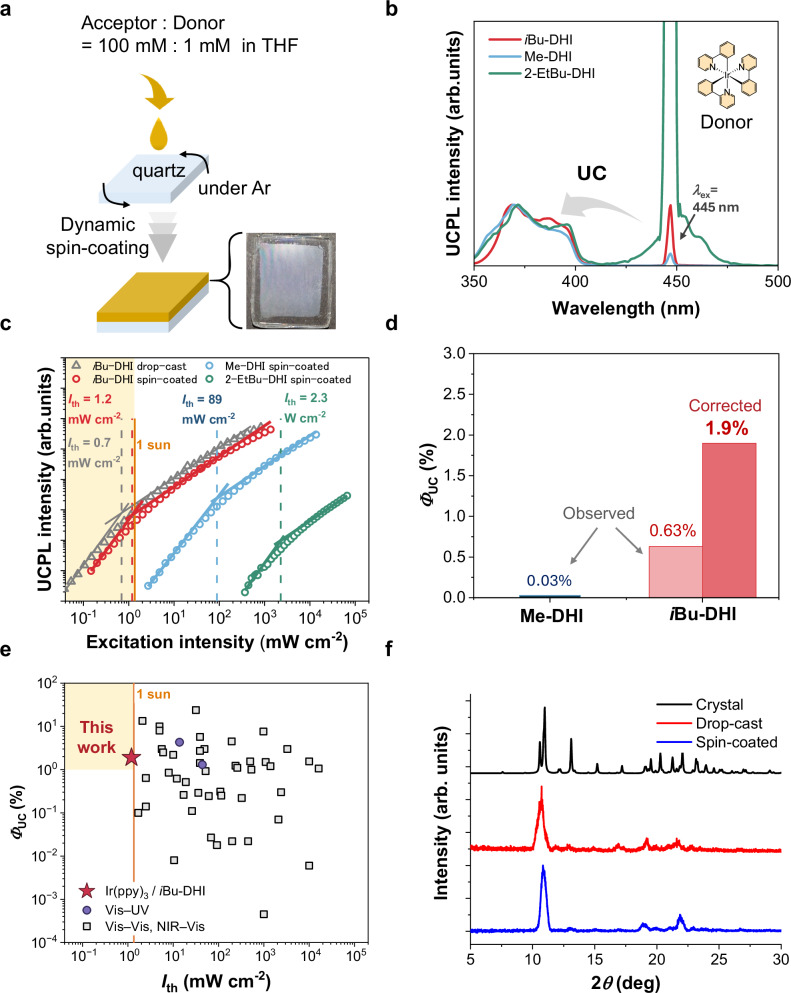
TTA-UC performances and characteristics of DHI-based UC solids. **a** Fabrication procedure of UC solid films by dynamic disperse spin-coating, **b** UC emission spectra, **c** Excitation intensity dependence of the upconverted emission intensity around 370 nm, and **d** absolute *Φ*_UC_ in solid of Me-DHI (blue), *i*Bu-DHI (red), and 2-EtBu-DHI (green) paired with Ir(ppy)_3_ (*λ*_ex_ = 445 nm, short-pass filter: 400 nm, molar ratio for preparation; acceptor : donor = 100 : 1). The gray plots in Fig. 3c show the data of *i*Bu-DHI/Ir(ppy)_3_ prepared by the drop-casting method. **e** *Φ*_UC_–*I*_th_ plots with the reported values of solid TTA-UC systems. Details of the references for the plot are provided in the supplementary information (Figure S32 and Table S8). **f** Comparison of X-ray diffraction patterns of crystalline solid of *i*Bu-DHI (black) and solid samples of *i*Bu-DHI with Ir(ppy)_3_ prepared by spin-coating (blue) and drop-casting (red) methods.

The π-orbital overlap was optimized not only in solution but also in the crystalline state, resulting in low *I*_th_ and long excited-state lifetimes. Spin-coated crystalline films of Me-DHI, *i*Bu-DHI, and 2-EtBu-DHI doped with Ir(ppy)_3_ showed clear UV upconversion emissions in the solid state (Fig. 3a, b). In contrast, a spin-coated DHI film did not exhibit detectable UC emission, likely due to its low *Φ*_F_. Among the three derivatives studied, *i*Bu-DHI exhibited the lowest *I*_th_ value (1.2 mW cm^−2^), which is considerably lower than those of previously reported donor–acceptor pairs (Fig. 3c, e). Based on the UC emission decay profiles, the *τ*_T_ of Me-DHI, *i*Bu-DHI, and 2-EtBu-DHI were determined to be 0.50 ms, 4.0 ms, and 4.6 ms, respectively (Figure S16). As revealed by the fluorescence quantum yields, the longer lifetimes of *i*Bu-DHI and 2-EtBu-DHI indicate suppressed quenching. Given the similar triplet lifetime between *i*Bu-DHI and 2-EtBu-DHI, the drastic reduction of the *I*_th_ value for *i*Bu-DHI/Ir(ppy)_3_ implies fast triplet energy diffusion in the crystalline solid^60^. The absolute *Φ*_UC_ was 1.9% after self-absorption correction (Fig. 3d, Figure S21)^61^. To our knowledge, this is the best value for the solid-state Vis-to-UV TTA-UC with *I*_th_ lower than 10 mW cm^−2^ ever reported. The overlap between the emission of *i*Bu-DHI and the absorption of Ir(ppy)_3_ in solution (Figure S7) would allow reabsorption and singlet back energy transfer (BET) from the acceptor to the donor in the donor-doped film. However, as the fluorescence lifetime of *i*Bu-DHI in the spin-coated film is almost unchanged (1.9 ns for the *i*Bu-DHI film, 1.8 ns for the *i*Bu-DHI/Ir(ppy)_3_ film, Figure S19b), the BET process is considered less effective. This small BET contribution is primarily ascribed to the intrinsically small molar absorption coefficient of Ir(ppy)_3_ (*ε*_370_ ~ 1.3 × 10^4 ^M^–1^ cm^–1^) around the acceptor emission peak (Figure S7). In addition, the short fluorescence lifetime ( ~ 2 ns) of the DHI derivatives would have made the BET less efficient (Figure S19, S20). In general, nanoscale phase separation could result in decrease TET and BET, but we could not confirm such sample inhomogeneity due to the limitation of the SEM-EDX experiments as mentioned later. The reabsorption could be further suppressed by using a sensitizer with minimal UV absorption. We also noticed that the drop-cast film of *i*Bu-DHI/Ir(ppy)_3_ shows an even lower *I*_th_ of 0.7 mW cm^–2^ with a sufficiently long *τ*_T_ of 3.8 ms (Fig. 3c, Figure S16d). As the triplet lifetimes of spin-coated and cast films are similar at 4.0 ms and 3.8 ms, respectively (Figure S16b, d), the lower *I*_th_ observed for the cast film indicates improved diffusion rate of triplet excitons possibly due to enhanced crystallinity resulting from the slower evaporation rate. We also found that when the spin-coated film of *i*Bu-DHI/Ir(ppy)_3_ was annealed at 120 °C above the melting point of *i*Bu-DHI (*T*_m_ ~ 110 °C), phosphorescence from Ir(ppy)_3_ was clearly observed from the microscope image (Figure S18f), and the UC emission became significantly lower (Figure S17), indicating the reduction of triplet sensitization efficiency due to the segregation. This also underscores the importance of kinetically controlling the sensitizer-doping process in this hybrid material. It is noteworthy that *i*Bu-DHI molecules achieve high donor–acceptor compatibility and excellent TTA-UC properties through simple film-forming techniques, such as room-temperature casting and spin coating, without requiring any special heating treatment ^28^.

**Fig. 4:**
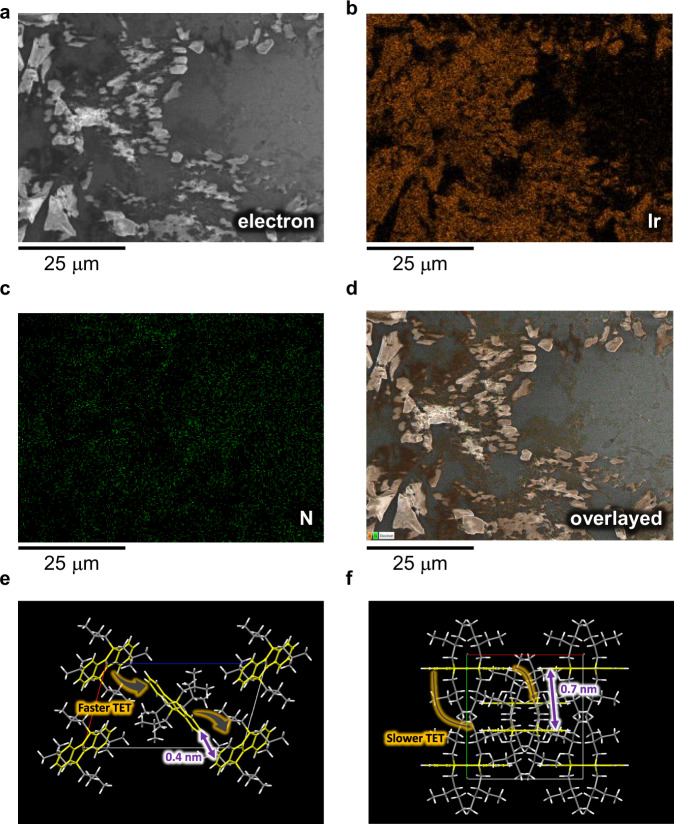
Sensitizer distribution and preferred TET pathways. **a** SEM image, and SEM-EDX mappings of **b** Ir, **c** N, and **d** their overlay for the drop-cast film of *i*Bu-DHI with Ir(ppy)_3_. Molecular packing structures in the crystal of **e** *i*Bu-DHI and **f** 2-EtBu-DHI. The purple double arrows indicate the distances of the closest carbon atoms of the adjacent π-planes. The gray arrows indicating the preferred directions of the triplet diffusion theoretically predicted by ab initio calculations are also shown (Table S7).

While segregation of sensitizers from the host annihilator crystals often causes a significant drop in sensitization efficiency and an increase in *I*_th_ values, this is not the case for the spin-coated or drop-cast crystalline DHI UC systems. Figure 4a–d and S22 show scanning electron microscopy (SEM) images with energy-dispersive X-ray (SEM-EDX) spectroscopy of *i*Bu-DHI and 2-EtBu-DHI crystals formed by spin-coating. Ir and N atoms of Ir(ppy)_3_ are homogeneously distributed in these microcrystals without micrometer-scale segregation, unlike the commonly observed inhomogeneous solid-state sensitizer–annihilator mixtures^7^. It is difficult to determine from SEM resolution whether the donors are located within the crystal or on its surface, or whether they are molecularly dispersed. However, the observation of high *Φ*_UC_ and low *I*_th_ in these crystal samples suggests that the donors are uniformly dispersed within the acceptor crystal, thereby enabling efficient triplet sensitization. The efficient energy transfer from Ir(ppy)_3_ to DHI derivatives in spin-coated films is confirmed by quenching of the donor phosphorescence. The main component of the phosphorescence decay profiles of Ir(ppy)_3_ dropped from 1.5 μs (in THF, Figure S8) or 45 ns (in the neat mixture solid, Figure S23, and Table S2) to less than 5 ns. By using the phosphorescence quantum yield of Ir(ppy)_3_ in the neat solid (*Φ*_P_ = 9.4%), the lower limit of TET efficiency to Me-DHI, *i*Bu-DHI, and 2-EtBu-DHI was estimated to be 59%, 71%, and 93%, respectively (Table S3). These features resemble the reported metal–organic framework UC system, which showed efficient triplet sensitization^62^. We also found that the upconversion emission of the *i*Bu-DHI can be sensitized by metal-free, thermally activated delayed fluorescence (TADF) sensitizers 4CzIPN^63^ and DABNA^64^ in spin-coated *i*Bu-DHI films (Figure S24). Thus, the Vis-to-UV TTA-UC in crystalline *i*Bu-DHI can also be achieved without the use of expensive Ir complexes. Our future work will report in-depth studies of these metal-free TTA-UC systems.

Another advantage expected for TTA-UC in high-density molecular assemblies is oxygen tolerance. The spin-coated film of *i*Bu-DHI/Ir(ppy)_3_ prepared in air-saturated conditions showed UC emission in air (Figure S25). A dense chromophore assembly can suppress oxygen diffusion while local triplet concentration increases in the excitation spot. TTA-UC is turned on when oxygen in the system is consumed by conversion to singlet oxygen^65–68^. The *i*Bu-DHI/Ir(ppy)_3_ film showed upconversion in air even under intense irradiation (*λ*_dt_ = 370 nm, *I*_ex_ = 2.0 W cm^–2^) for more than 1 h. This photodecomposition was suppressed by sealing in an inert atmosphere (Figures S25a and S25e).

To elucidate the relationship between the structure and UC performance, theoretical calculations of the TET and TTA reaction times were conducted for single-crystal structures of Me-DHI, *i*Bu-DHI, and 2-EtBu-DHI. The X-ray diffraction patterns of the single crystals and those of the spin-coated films were similar (Fig. 3f), and we evaluated the intermolecular geometries of the dimers in crystals (Fig. 4e, f). In the case of the *i*Bu-DHI crystal, the closest atom–atom distance of the DHI units packed in the crystal structure is 0.4 nm (Fig. 4e), which is much shorter than that of the 2-EtBu-DHI crystal (0.7 nm), the latter forming parallel-displaced stacking (Fig. 4f). The difference in the electronic coupling matrix elements in these packing structures, and the TET and TTA reaction times were theoretically estimated using the ab initio and DFT/TD-DFT calculations, followed by the methods similar to those of Sato and Shigeta et al. (Figure S30, S31, and Table S7)^69^. The direction giving the fastest reaction times of TET and TTA in *i*Bu-DHI and 2-EtBu-DHI, which are most likely to contribute to the triplet dynamics, are shown in Figure 4e, f. Thanks to sufficient π-orbital interaction between the edges of the π-planes, a moderate electronic coupling strength is realized for *i*Bu-DHI, providing a TET reaction time of 1.25 μs, which is a four orders of magnitude faster than that of 2-EtBu-DHI (4.20 × 10^4^ μs for pair ***a***, 2.85 × 10^4^ μs for pair ***b***). The TTA reaction times in the same directions were calculated to be 4.54 ns and 8.09 × 10^1 ^ns (for pair ***a***) or 1.10 × 10^6 ^ns (for pair ***b***) for *i*Bu-DHI and 2-EtBu-DHI, respectively (Figure S30, S31, Table S7). Although nanoscale donor–acceptor interface structures may affect TET efficiency, the calculated values explain the three orders of magnitude difference in *I*_th_ between *i*Bu-DHI and 2-EtBu-DHI, which is inversely proportional to the TTA rate constants, often approximated with the triplet diffusion rate constant. Note that the TET and TTA reaction times between Me-DHI and *i*Bu-DHI were similar (Table S7), which is also consistent with the *I*_th_ difference explained mainly by the triplet lifetime (∝τ_T_^−2^). Therefore, we concluded that the molecular organization of *i*Bu-DHI, with a proper balance of shielded and exposed π-surfaces provided by alkyl chains and adequate molecular distance, contributed to the low *I*_th_ and high *Φ*_UC_. The performance of the present solid-state Vis-to-UV TTA-UC system could be further improved by optimizing the donor molecular structure and employing a controlled crystallization process ^29^.

## Discussion

In this study, highly efficient Vis-to-UV TTA-UC in solid crystals was achieved by alkyl extension in the out-of-π-plane direction from the *sp*^3^ carbon of the acceptor dihydroindenoindene skeleton. While chromophores with bulky *tert*-butyl or phenyl groups have often been used in TTA-UC to suppress excimer formation in solution, the placement of these bulky substituents relative to the π-orbitals has not been controlled, and these conventional systems have limited success in solid-state TTA-UC (Figure S32–S36). The preset molecular design enabled us to precisely control the overlap between the π-orbitals and the intermolecular distances.

In the systematic investigation of TTA-UC for alkylated DHI derivatives in solution, the introduction of side chains longer than a methyl group was required to suppress quenching of the triplets. We demonstrated that *i*Bu-DHI is the best acceptor in the studied DHI series, and the importance of introducing bulky substituents to TTA-UC acceptors explains why acceptors with a triisopropylsilyl ethynyl group have shown efficient TTA-UC in solution^50,70–72^. The high *Φ*_UC_ of 10% observed for *i*Bu-DHI in the solution state is attributed to a high fluorescence quantum yield of around 90%, which is much larger than TIPS-Nph (*Φ*_F_ = 66%) ^54^.

Notably, the introduction of alkyl chains also allows the DHI chromophore to be isolated in the crystalline state at appropriate intermolecular distances. The simultaneous achievement of the high fluorescence quantum yield, long excited triplet lifetime, and efficient triplet energy migration in crystalline DHI systems would be explained by the fact that in the adequately isolated chromophore system, the singlet and triplet energy levels are less affected by the different relative orientations of the chromophores, and the deactivation due to the strong intermolecular interaction is suppressed. These unique features make *i*Bu-DHI the best acceptor to date, working in the crystalline state with a high *Φ*_UC_ of 1.9% and a low threshold excitation intensity of 1.2 mW cm^–2^. Note that while the significantly low *I*_th_ values and high *Φ*_UC_ in the films formed by spin coating or drop casting are attractive for low-cost fabrication, these processes are not suitable for controlling crystal sizes and grain boundaries, which are necessary to minimize batch or processing condition-dependence in UC and PL performance. Optimization of hybrid structures via processes such as controlled crystallization may improve UC performance by enabling control over crystal size and grain boundaries^29^. The dense molecular organization in crystals confers tolerance to TTA-UC performance under air, a distinct feature from the conventional UC system using polymer hosts or solvents, where oxygen efficiently quenches triplets. The design principle of the π-protected DHI chromophores developed in this study will be widely extended to various chromophores. It enables excellent TTA-UC properties in thin films prepared by simple spin-coating and drop-casting methods, paving the way for broad applications and promising to revolutionize photofunctional chemistry involving excited triplets.

## Methods

### Materials

All reagents and solvents were used as received without further purification, unless otherwise noted. Ir(ppy)_3_ was purchased from Wako. For TTA-UC measurements in the solution state, all samples were prepared in an Ar-filled glovebox ([O_2_] <0.1 ppm, [H_2_O] <0.1 ppm) using deoxidized tetrahydrofuran (THF) purchased from Wako. 5,10-Dihydroindeno[2,1-*a*]indene (DHI) and 5,5,10,10-tetramethyl-5,10-dihydroindeno[2,1-*a*]indene (Me-DHI) were purchased from TCI and Sigma Aldrich, respectively. These were then recrystallized from acetone or methanol, and their purity was confirmed by ^1^H-NMR and elemental analysis. The details were written in the supplementary information. Coumarin 6, 4CzIPN, and DABNA were purchased from TCI, Ossila, and NARD Institute, Ltd., respectively.

### Sample preparation

To prepare the spin-coated solid-state UC sample of the DHI derivative (Me-DHI, *i*Bu-DHI, or 2-EtBu-DHI) with Ir(ppy)_3_, 20 μmol of the DHI derivative and 200 nmol of Ir(ppy)_3_ were dissolved in 0.20 mL of THF in an Ar-filled glovebox. One drop of the solution was placed on a rotating quartz substrate at 1200 rpm, and the rotation was kept for 1 min. The exact process was repeated six times. The sample was then sandwiched between two quartz substrates, and the edges were sealed with ThreeBond 2086 M.

The drop-cast films were prepared with a THF solution of the DHI derivative and Ir(ppy)_3_ ([DHI derivative]: [Ir(ppy)_3_] = 1 mM: 100 μM or 10 mM: 100 μM). These solutions were applied to the substrate dropwise (about 0.5 mL in total), dried, and sealed using the same method.

### Characterization

For synthesis and purification, ^1^H-NMR (400 MHz) and ^13^C-NMR (101 MHz) spectra were measured on a JNM-ECZ 400S, and tetramethylsilane (TMS) was used as the internal standard. Elemental analysis was performed using Yanaco CHN Corder MT-5 at the Elemental Analysis Center, Kyushu University (absolute error ≤ ± 0.3%).

For photophysical characterization, UV–Vis absorption spectra were recorded using a JASCO spectrophotometers (V-670 and V-770). Photoluminescence (PL) spectra were obtained with a JASCO FP-8700. Fluorescence of the spin-coated film was detected along the normal direction by excitation at an angle of incidence of 30° to the surface. The fluorescence of the solution sample was collected at 90° to the incident exciting beam. The absolute fluorescence quantum yield was measured using an integrating sphere with a HAMAMATSU multichannel analyzer C10027-01. A time-correlated single-photon counting lifetime spectroscopy (TCSPC) system, Quantaurus-Tau C11367-02, was used to perform time-resolved PL lifetime measurements. Fluorescence and phosphorescence decay curves were fitted by single or multi exponential functions. The triplet lifetime (*τ*_T_) was estimated by fitting the tail of the UC emission decay profile with the following relationship^73^

1 $${I}_{{{\rm{UC}}}}\left(t\right)\propto \exp \left(\frac{-t}{{\tau }_{{{\rm{UC}}}}}\right)=exp \left(\frac{-2t}{{\tau }_{{{\rm{T}}}}}\right)$$

where *τ*_UC_ and *τ*_T_ are the UC emission lifetime and acceptor triplet lifetime, respectively. The lower limit of TET efficiency (*Φ*_TET_) in the solid state was estimated by the following equation.

2 $${\varPhi }_{{{\rm{TET}}}}=1-\frac{{\varPhi }_{{{\rm{P}}}}}{{\varPhi }_{{{\rm{P}}},0}}$$

For TTA-UC measurements, a 445 nm diode laser (75 mW, RGB Photonics) was used as the excitation light source. A PD300-UV photodiode sensor (OPHIR Photonics) and an SP620 CCD beam profiler (OPHIR Photonics) were used to measure the excitation light intensity and the laser beam profile (diameter). A program software (Ltune) and a rotatable variable neutral density filter were used to control the laser power. Achromatic lenses were used to focus the emitted light from the sample onto an optical fiber that was connected to an MCPD-9800 multichannel detector (Otsuka Electronics). A 400 nm short-pass filter was used between the sample and the detector in the solid-state measurements. The detector was calibrated in the range from 350 to 950 nm using a standard lamp HL-3 plus-CAL (Ocean Optics). Short-pass and long-pass filters were not used between the sample and the detector during the calibration measurement.

For SEM-EDX mappings, S-5200 (Hitachi High-Tech) was used with 2 kV acceleration voltage. A carbon coater SC-701C (SANYU ELECTRON) was used to coat the sample surface with carbon to prevent charge-up from electron beams. Note that increasing the acceleration voltage of the electron beams with SEM above ~15 keV caused significant damage that melted *i*Bu-DHI and 2-EtBu-DHI samples. For X-ray diffraction measurements, D2 PHASER 2nd Generation (BRUKER) and SmartLab (Rigaku) were used. Step-height analysis with a Surfcorder ET150 (Kosaka Laboratory Ltd.) revealed the following average thicknesses for the Ir(ppy)_3_ doped spin-coated films of Me-DHI, *i*Bu-DHI, and 2-EtBu-DHI: 82.2 nm, 128 nm, and 297 nm, respectively.

### Determination of threshold excitation intensity

The excitation intensity threshold (*I*_th_) for TTA-UC was determined from the intersection point of the linear fits for the low- and high-excitation regimes in the log–log plot of UC emission intensity (Fig. 3c). The obtained slopes transitioned from 1.9 to 1.0 for Me-DHI, from 2.0 to 1.0 for *i*Bu-DHI (for both drop-cast and spin-coated films), and from 2.1 to 1.0 for 2-EtBu-DHI.

### Calculation of UC quantum yield by the relative method

UC quantum yield (*Φ*_UC_) in deaerated THF was calculated by a relative method using Coumarin 6 in deaerated THF (20 μM) as a standard, according to the following equation,

3 $${\varPhi }_{{{\rm{UC}}}}={\varPhi }_{{{\rm{Std}}}}\left(\frac{1-{10}^{{-A}_{{{\rm{Std}}}}}}{1-{10}^{{-A}_{{{\rm{UC}}}}}}\right)\left(\frac{{E}_{{{\rm{UC}}}}}{{E}_{{{\rm{Std}}}}}\right)\left(\frac{{I}_{{{\rm{Std}}}}}{{I}_{{{\rm{UC}}}}}\right){\left(\frac{{n}_{{{\rm{UC}}}}}{{n}_{{{\rm{Std}}}}}\right)}^{2}$$

where *Φ*, *A*, *E*, *I*, and *n* are the quantum yield, absorbance at 445 nm, integrated PL spectral profile, excitation light intensity, and refractive index of the solvent, respectively. The subscripts UC and Std refer to the system of upconversion and standard samples, respectively.

### Determination of UC quantum yield by the absolute method

To measure absolute UC quantum yields, Quantaurus-QY Plus (HAMAMATSU) was used with a blue diode laser module at 450 nm (MDL-III-450). The correction process was conducted in accordance with the previous literature^61^. To obtain a sufficient absorption coefficient and a uniform surface, we introduced dynamic spin coating as described above.

### Computational details

Density functional theory (DFT) and time-dependent DFT (TD-DFT) calculations were conducted with the B3LYP exchange-correlation functional as implemented in the Gaussian 16 suite of programs (Rev. B.01^74^ for 2-EtBu-DHI, and Rev. C.01^75^ for DHI, Me-DHI, and *i*Bu-DHI). The 6-311 + + G(d,p) basis sets were used for the ground-state geometry optimizations and excited-state calculations, respectively. Vibrational frequency analyses were also performed to ensure the converged structures reached the local minima. Spin density surfaces were visualized by the GaussView 6.1.1 ^76^.

For the theoretical analysis of triplet energy transfer and triplet–triplet annihilation processes in crystals, the electronic coupling matrix elements ( | *T*_DA_ | ) and reaction times (*τ*) of TTA and TET were examined by the ab initio and DFT/TD-DFT calculations. The details of the calculations were referred to those in previous works and described in the supplementary information (Suppl. Note 16) ^69,77–79^.

### Crystallographic data

CCDC 2433438 (10.5517/ccdc.csd.cc2mp60k), 2433439 (10.5517/ccdc.csd.cc2mp61l), and 2433440 (10.5517/ccdc.csd.cc2mp62m) contain the supplementary crystallographic data for this paper. The data can be obtained free of charge via (www.ccdc.cam.ac.uk/data_request/cif), by emailing data_request@ccdc.cam.ac.uk, or by contacting The Cambridge Crystallographic Data Center, 12 Union Road, Cambridge CB2 1EZ, UK; fax: + 44 1223 336033.

## Supplementary information

Supplementary Information

Transparent Peer Review file

## Source data

Source Data

## Data Availability

Source data in this study are provided in the Source Data file. The input and output files for the DFT calculations and theoretical analysis of TET and TTA reaction times are deposited in the Zenodo data repository (10.5281/zenodo.20318973). Source data are provided with this paper.
