# Supplementary material for: Sterically protected π-electron systems for efficient solid-state photon upconversion
Source: Nat Commun. 2026 Jun 23;17:5134. doi: 10.1038/s41467-026-73898-0 (PMC13291331; doi:10.1038/s41467-026-73898-0)
Supplement: Supplementary file 1 — Supplementary Information [file 41467_2026_73898_MOESM1_ESM.pdf]

Supplementary Information

Sterically Protected π-Electron Systems for Efficient Solid-State Photon Upconversion

Naoyuki Harada[1†], Hayato Shoyama[1†], Nutnicha Boonmong[1†], Kiichi Mizukami[1], Yuya Watanabe[1], Pei Zhao[2], Masahiro Ehara[2], Yoichi Sasaki[*1,3,4], and Nobuo Kimizuka[*1,3,4]

[1]*Department of Applied Chemistry, Graduate School of Engineering, Kyushu University, 744 Moto-oka, Nishi-ku, Fukuoka 819-0395, Japan.*

[2]*Research Center for Computational Science, Institute for Molecular Science, SOKENDAI, 38 Nishigo-Naka, Myodaiji, Okazaki 444-8585, Japan.*

[3]*Center for Molecular Systems (CMS), Kyushu University, 744 Moto-oka, Nishi-ku, Fukuoka 819-0395, Japan.*

[4]*Research Center for Negative Emissions Technologies (K-NETs), Kyushu University, 744 Moto-oka, Nishi-ku, Fukuoka 819-0395, Japan.*

† These authors equally contributed to this work

[*]Corresponding authors (sasaki.yoichi.772@m.kyushu-u.ac.jp, kimizuka.nobuo.763@m.kyushu-u.ac.jp)

Supplementary Contents

Suppl. Note 1. Characterization of DHI and Me-DHI 3

1.1. Characterization of 5,10-dihydroindeno[2,1-*a*]indene (DHI) 3

1.2. Characterization of 5,5,10,10-Tetramethyl-5,10-dihydroindeno[2,1-*a*]indene (Me-DHI) 3

Suppl. Note 2. Synthesis of DHI derivatives 4

2.1. Synthesis of *i*Bu-DHI 4

2.2. Synthesis of 2-EtBu-DHI 6

Suppl. Note 3. Density functional theory (DFT) calculations 8

Suppl. Note 4. Basic photophysical properties in solution 10

Suppl. Note 5. Photon upconversion properties in solution 12

Suppl. Note 6. Photostability of the UC emission in solution 16

Suppl. Note 7. Formation of aggregates in the mixed solution of $Ir(ppy)_3$ and 2-EtBu-DHI 17

Suppl. Note 8. UC profiles in solid states 19

Suppl. Note 9. Fluorescence decay profiles in solid states 22

Suppl. Note 10. Self-absorption correction of an absolute UC quantum yield 24

Suppl. Note 11. Scanning electron microscopy with energy dispersive X-ray spectroscopy (SEM-EDX) 25

Suppl. Note 12. Phosphorescence decay profiles and quantum yield in solids 26

Suppl. Note 13. UC emission from heavy metal-free sensitizer-doped films 29

Suppl. Note 14. Evaluation of oxygen tolerance and stability of TTA-UC 30

Suppl. Note 15. Crystal structures of DHI derivatives 31

Suppl. Note 16. Theoretical analysis of triplet energy transfer and triplet–triplet annihilation reaction times in crystals 38

Suppl. Note 17. Reported solid-state TTA-UC properties 43

Supplementary References 50

Suppl. Note 1. Characterization of DHI and Me-DHI

1.1. Characterization of 5,10-dihydroindeno[2,1-*a*]indene (DHI)

^{1}H-NMR (400 MHz, Chloroform-*D*, TMS) δ (ppm) 7.51 (d, *J* = 7.3 Hz, 2H), 7.43 (d, *J* = 7.6 Hz, 2H), 7.31 (td, *J* = 7.5, 0.7 Hz, 2H), 7.19 (td, *J* = 7.4, 0.9 Hz, 2H), 3.63 (s, 4H).

Elemental analysis, calculated for $C_{16}H_{12}$: H 5.92 C 94.08 N 0.00; found H 6.04, C 94.05 N 0.02.

1.2. Characterization of 5,5,10,10-Tetramethyl-5,10-dihydroindeno[2,1-*a*]indene (Me-DHI)

^{1}H-NMR (400 MHz, Cyclohexane-*D12*, TMS) δ (ppm) 7.28 (dd, *J* = 2.3, 1.0 Hz, 2H), 7.26 (dd, *J* = 2.5, 0.7 Hz, 2H), 7.14 (td, *J* = 7.5, 1.0 Hz, 2H), 7.07 (td, *J* = 7.4, 1.1 Hz, 2H), 1.48 (s, 12H).

Elemental analysis, calculated for $C_{20}H_{20}$: H 7.74 C 92.26 N 0.00; found H 7.70, C 92.09 N 0.04.

Suppl. Note 2. Synthesis of DHI derivatives

2.1. Synthesis of *i*Bu-DHI

*i*Bu-DHI was synthesized according to the literature with some modifications.[1] 5,10-Dihydroindeno[2,1-*a*]indene (1.01 g, 4.96 mmol) was added to a three-necked flask and dissolved in dehydrated tetrahydrofuran (10.0 mL) under a nitrogen atmosphere. The flask was submerged in a cooling bath (liquid nitrogen/methanol) at –98 °C for 30 minutes. To this solution, *n*-butyllithium (2.6 mol/L in *n*-hexane solution, 5.0 mL, 13.0 mmol) was added slowly dropwise. Isobutyl bromide (1.86 g, 13.6 mmol) was added slowly, and then the flask was purged with nitrogen once again. The flask was brought to room temperature and stirred for 13 hours. Saturated hydrochloric acid (0.40 mL) and sodium thiosulfate (10 mL) were added, and the mixture was stirred for another 1 h. The solvent was removed under reduced pressure. The target product was extracted using a phase separator. The crude product obtained was purified by flash column chromatography using *n*-hexane as an eluent, followed by recrystallization from acetone. *i*Bu-DHI was obtained as a colorless solid (yield: 0.29 g (14%)).

^{1}H-NMR (400 MHz, Cyclohexane-*D12*, TMS) δ (ppm) 7.31–7.21 (m, 4H), 7.15 (td, J = 7.4, 0.8 Hz, 2H), 7.05 (td, J = 7.6, 0.8 Hz, 2H), 2.08 (dd, J = 13.4, 3.8 Hz, 4H), 1.97 (dd, J = 13.6, 6.7 Hz, 4H), 1.06 (m, 4H), 0.63 (d, J = 6.7 Hz, 12H), 0.37 (d, J = 6.9 Hz, 12H).

^{13}C-NMR (101 MHz, Cyclohexane-*D12*, TMS) δ (ppm) 156.24 (d, J = 19.1 Hz), 141.64, 127.23, 124.68, 123.43, 119.96, 54.67, 49.35, 25.83, 25.28, 24.96.

Elemental analysis, calculated for $C_{32}H_{44}$: H 10.35 C 89.65 N 0.00; found H 10.36, C 89.54 N 0.05.

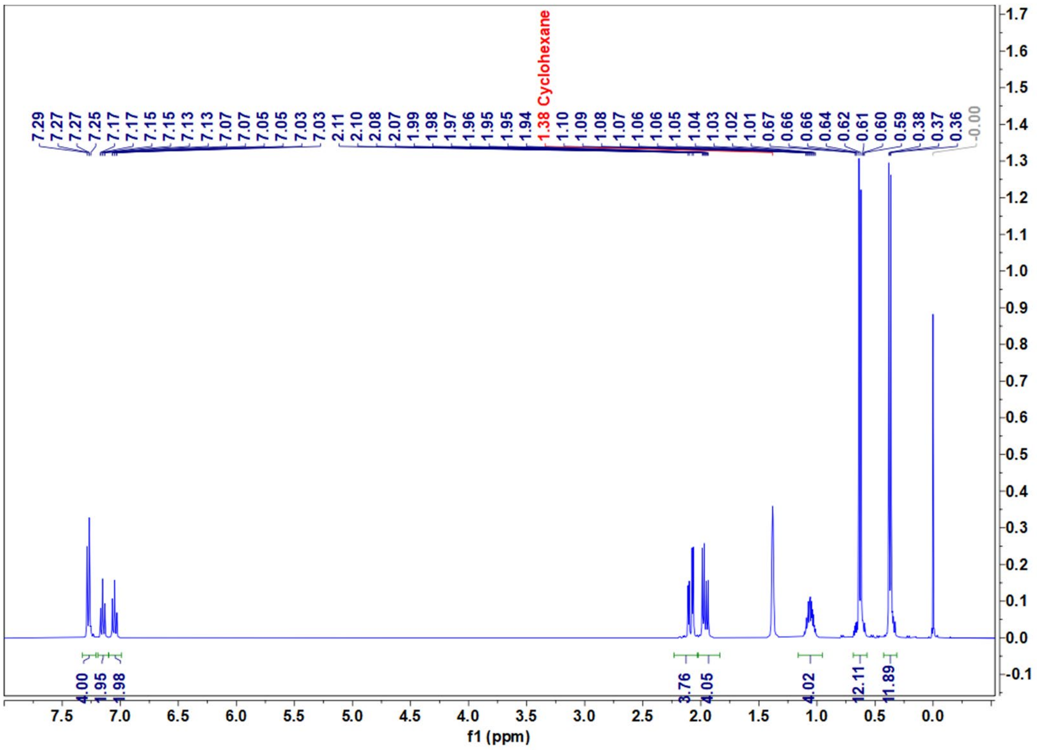

Supplementary Figure 1. ^{1}H-NMR spectrum of *i*Bu-DHI (400 MHz, Cyclohexane-*D12*, TMS).

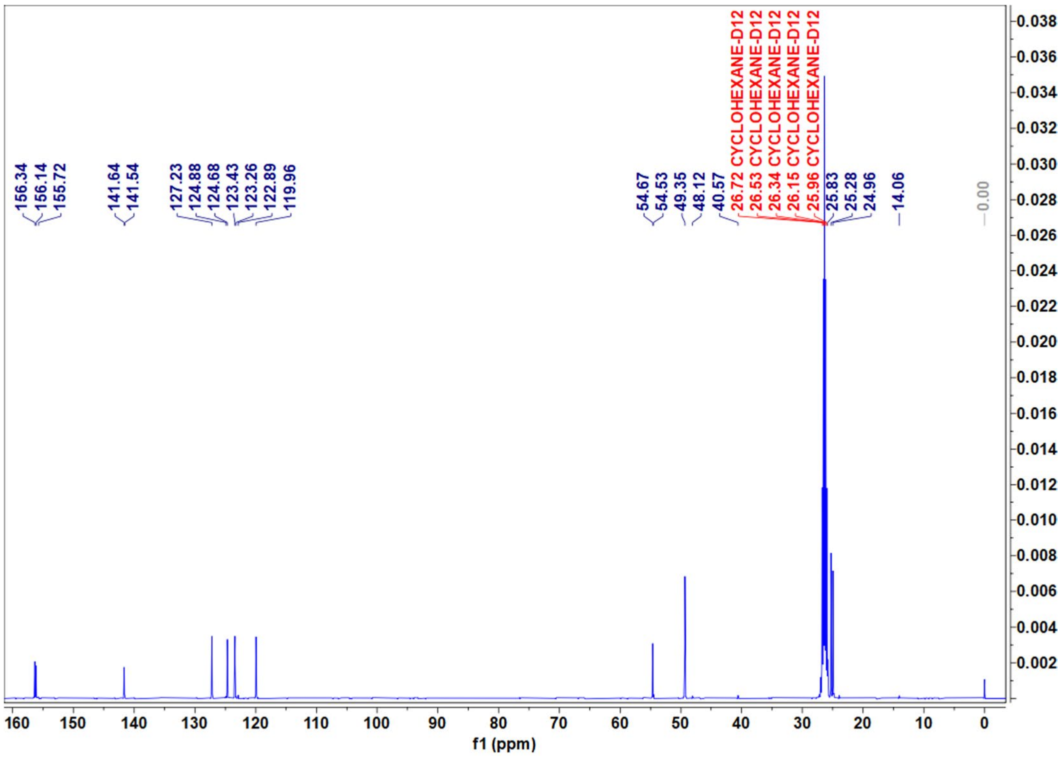

Supplementary Figure 2. ^{13}C-NMR spectrum of *i*Bu-DHI (101 MHz, Cyclohexane-*D12*, TMS).

2.2. Synthesis of 2-EtBu-DHI

2-EtBu-DHI was synthesized according to the literature with some modifications.[1] 5,10-Dihydroindeno[2,1-*a*]indene (2.04 g, 10.0 mmol) was added to a three-necked flask and dissolved in dehydrated tetrahydrofuran (50.0 mL) under a nitrogen atmosphere. The flask was submerged in a cooling bath (dry ice/acetone) at –78 °C for 30 minutes. To this solution, *n*-butyllithium (1.6 mol/L in *n*-hexane solution, 25.0 mL, 40.0 mmol) was added slowly dropwise. 1-Bromo-2-ethylbutane (5.00 g, 30.3 mmol) was added slowly, and the flask was purged with nitrogen once again. The flask was brought to room temperature and stirred for 24 hours. Saturated hydrochloric acid (4.00 mL) and sodium thiosulfate (10 mL) were added, and the mixture was stirred for another 1 h. After solvent removal under reduced pressure, the mixture was extracted with water and toluene, and the organic layer was dehydrated with anhydrous sodium sulfate and removed under reduced pressure. The crude product obtained was purified by flash column chromatography using *n*-hexane as the eluent, followed by successive recrystallizations from acetone and methanol. 2-EtBu-DHI was obtained as a colorless solid (yield: 0.72 g (13%)).

^{1}H-NMR (400 MHz, Methylene Chloride-*D2*, TMS) δ (ppm) 7.37 (dd, *J* = 16.1, 6.2 Hz, 4H), 7.23 (td, *J* = 7.6, 1.0 Hz, 2H), 7.11 (td, *J* = 7.6, 1.2 Hz, 2H), 2.01 (d, *J* = 4.2 Hz, 8H), 1.08–0.95 (m, 8H), 0.96 – 0.66 (m, 12H), 0.60 (t, *J* = 7.3 Hz, 12H), 0.50 (t, *J* = 7.2 Hz, 12H).

^{13}C-NMR (101 MHz, Methylene Chloride-*D2*, TMS) δ (ppm) 156.01 (d, *J* = 4.8 Hz), 141.11, 126.58, 123.98, 123.69, 120.14, 42.43, 36.01, 26.90, 26.49, 10.45, 9.87.

Elemental analysis, calculated for $C_{40}H_{60}$: H 11.18 C 88.82 N 0.00; found H 11.06, C 88.61, N 0.01.

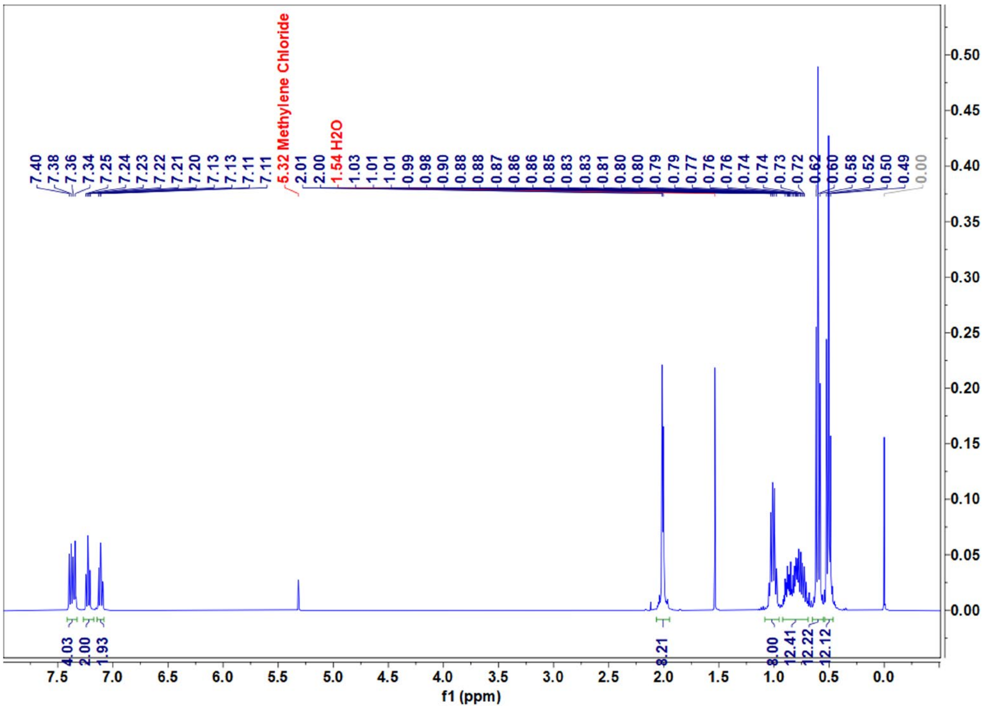

Supplementary Figure 3. ^{1}H-NMR spectrum of 2-EtBu-DHI (400 MHz, Methylene Chloride-*D2*, TMS).

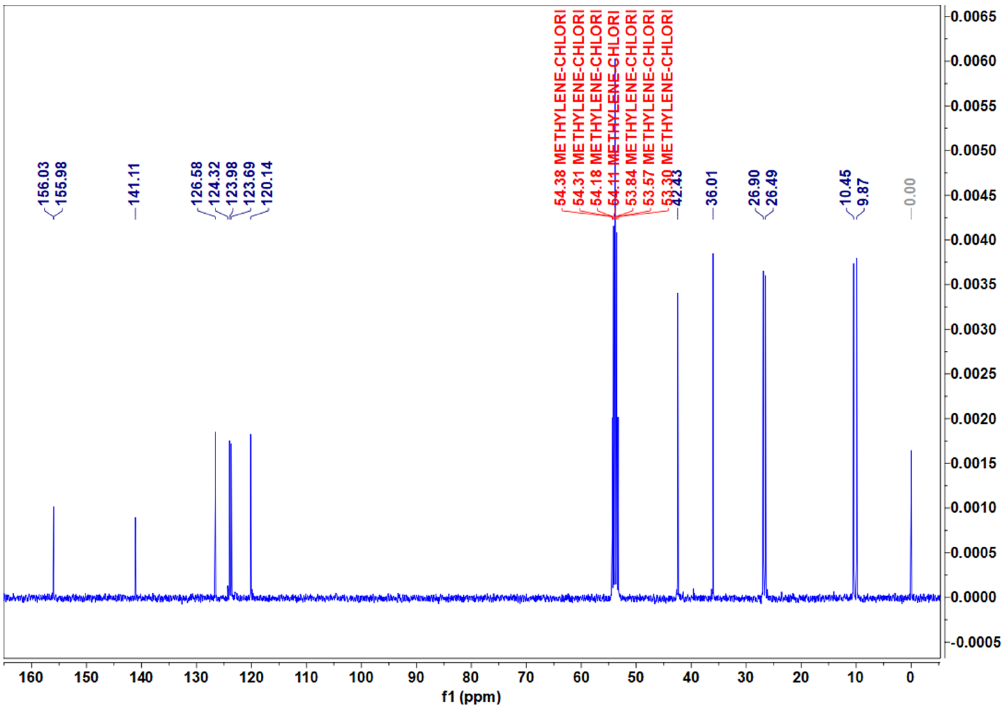

Supplementary Figure 4. ^{13}C-NMR spectrum of 2-EtBu-DHI (101 MHz, Methylene Chloride-*D2*, TMS).

Suppl. Note 3. Density functional theory (DFT) calculations

Density functional theory (DFT) and time-dependent DFT (TD-DFT) calculations were conducted with the B3LYP exchange-correlation functional as implemented in the Gaussian 16 suite of programs (Rev. B.01[2] for 2-EtBu-DHI, and Rev. C.01[3] for DHI, Me-DHI, and *i*Bu-DHI). The 6-311++G(d,p) basis sets were used for the ground-state geometry optimizations and excited-state calculations, respectively. Vibrational frequency analyses were also performed to ensure the converged structures reached the local minima. Spin density surfaces were visualized by the GaussView 6.1.1.[4]

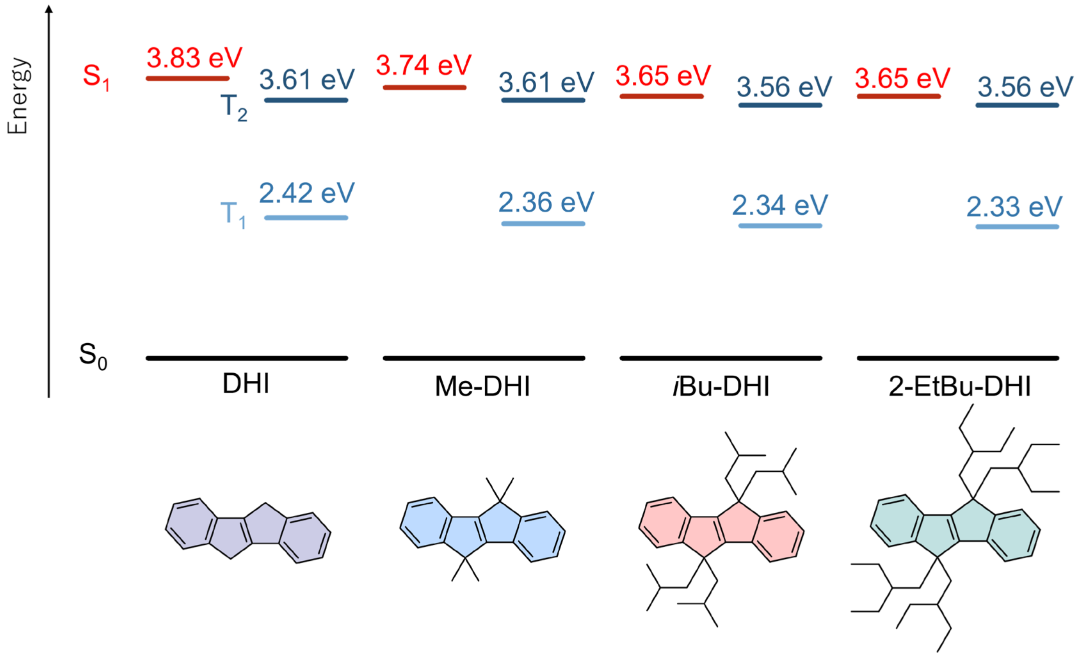

Supplementary Figure 5. Calculated singlet and triplet energy levels of DHI, Me-DHI, *i*Bu-DHI, and 2-EtBu-DHI by DFT/TD-DFT calculations (TD-B3LYP/6-311++G(d,p) // B3LYP/6-311++G(d,p)).

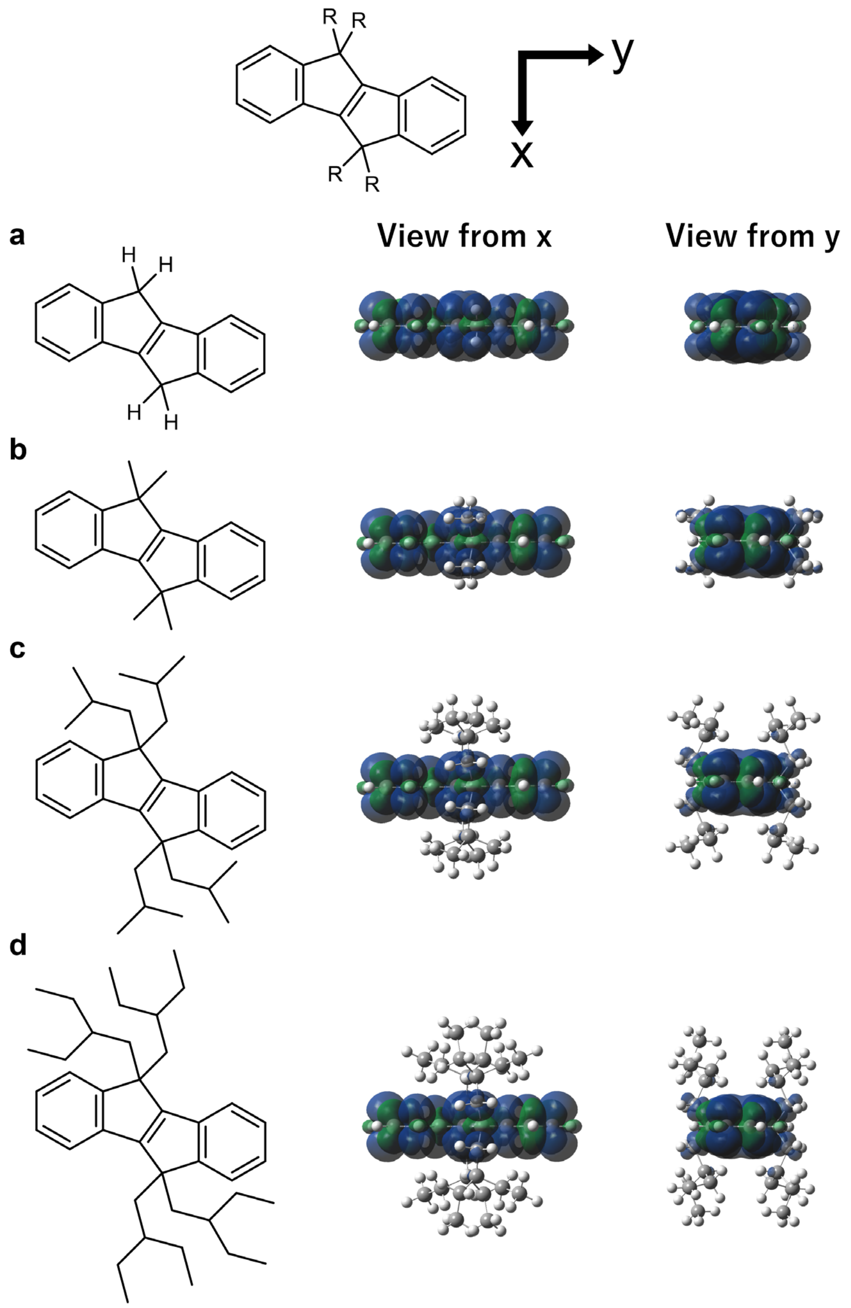

Supplementary Figure 6. Calculated spin density surfaces for DHI and its derivatives in the T_1 state (**a** DHI, **b** Me-DHI, **c** *i*Bu-DHI, and **d** 2-EtBu-DHI). All surfaces are shown for T_1-optimized structures using an iso-surface value of 0.0004.

Suppl. Note 4. Basic photophysical properties in solution

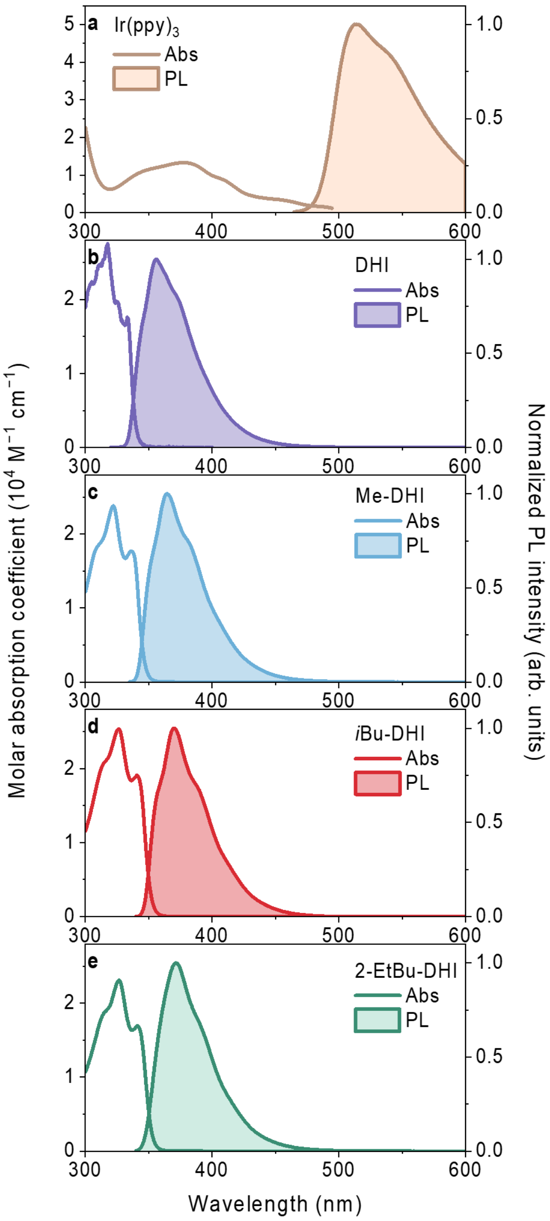

Supplementary Figure 7. Absorption spectra (molar absorption coefficient) (solid line) and normalized emission (filled in) spectra of **a** $Ir(ppy)_3$, **b** DHI, **c** Me-DHI, **d** *i*Bu-DHI, and **e** 2-EtBu-DHI. The samples were prepared in deaerated THF ($[Ir(ppy)_3]$ = 100 μM, [DHI/DHI derivative] = 200 μM).

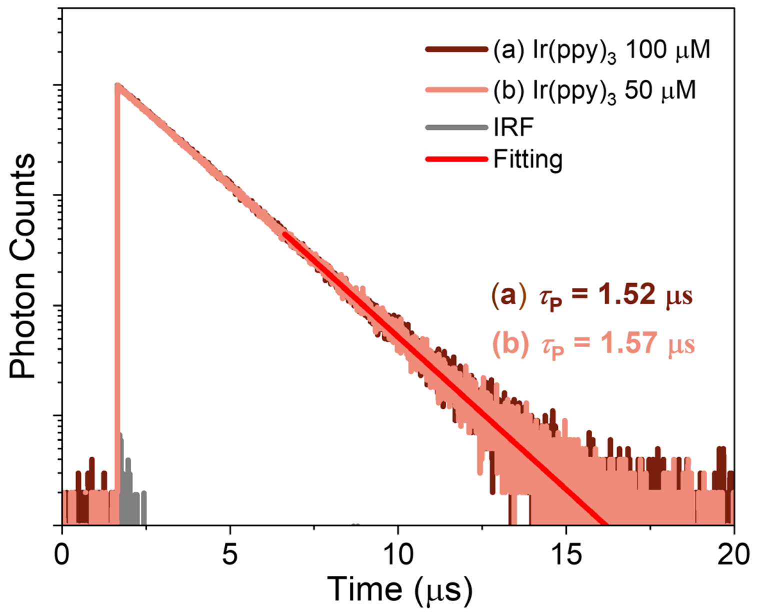

Supplementary Figure 8. Phosphorescence emission decay profiles of $Ir(ppy)_3$ in deaerated THF. They were recorded with excitation at 405 nm, with detection at 525 nm. The light gray lines show the instrumental response function (IRF). Mono-exponential fitting yielded lifetimes of 1.52 μs (100 μM, (a) wine red) and 1.57 μs (50 μM, (b) coral red), respectively.

Suppl. Note 5. Photon upconversion properties in solution

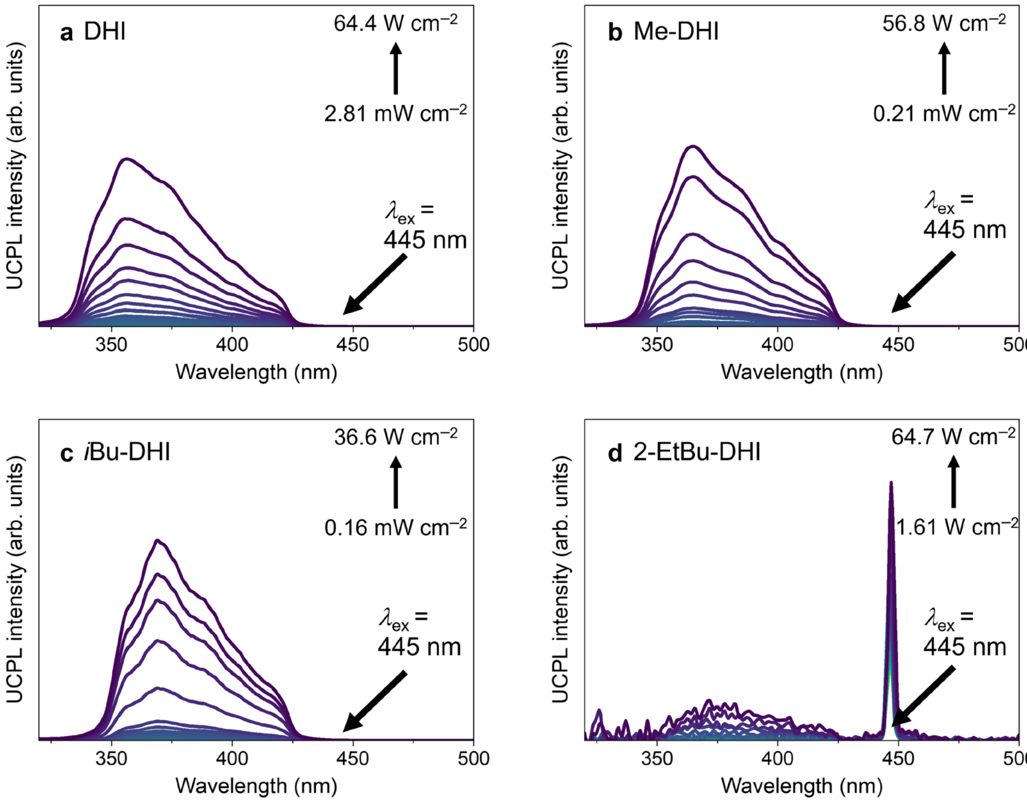

Supplementary Figure 9. Excitation intensity-dependent UCPL spectra for **a** DHI and its derivatives (**b** Me-DHI, **c** *i*Bu-DHI, and **d** 2-EtBu-DHI) at an acceptor concentration of 1 mM with 50 μM Ir(ppy)$_3$ in deaerated THF. Excitation light was removed with a 425 nm short-pass filter. Aggregation of 2-EtBu-DHI was observed upon 445 nm laser excitation, which may account for the negligible UC emission (see below for details).

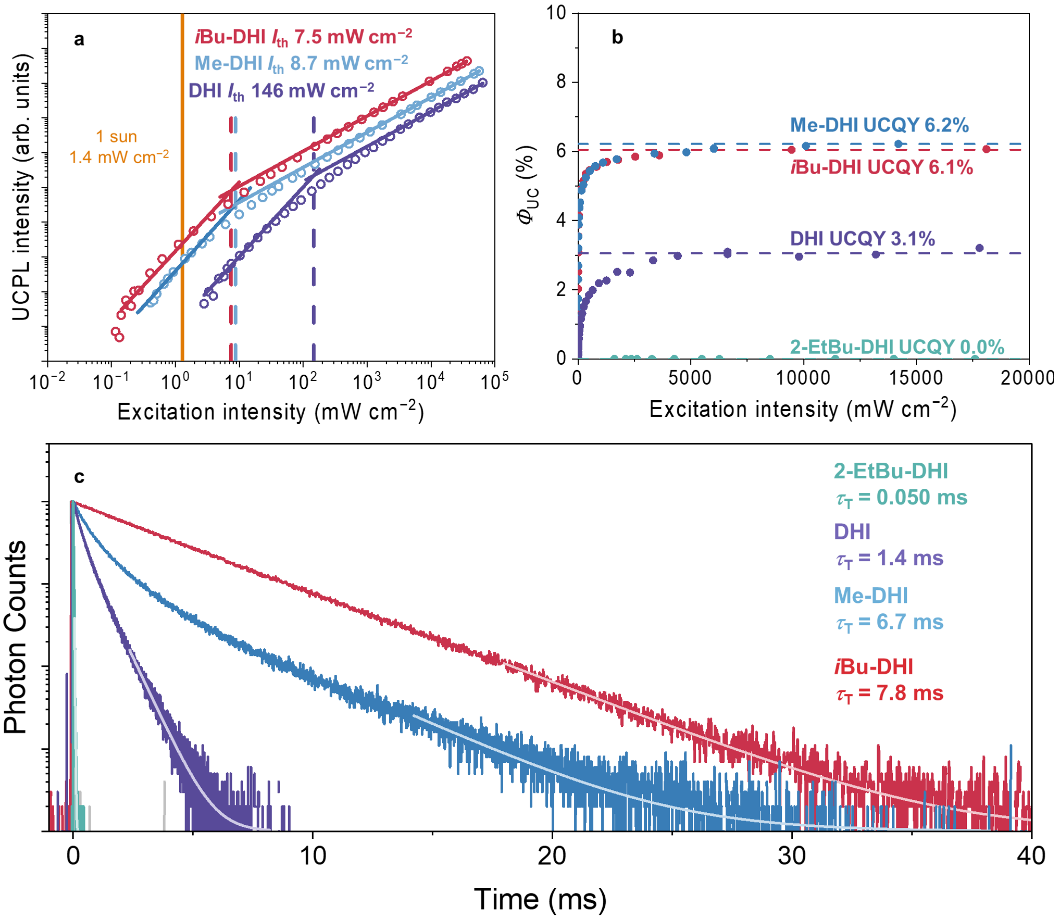

Supplementary Figure 10. Upconversion properties for DHI derivatives. **a** Plots of excitation intensity-dependent UC emission intensity. The slopes obtained from the linear fits transitioned from 2.0 to 1.0 for DHI, Me-DHI, and *i*Bu-DHI. **b** Plots of Φ_{UC} at various excitation intensities in deaerated THF. The yellow line represents the intensity of light in the 445±5 nm range in sunlight. **c** UC emission decay curves of DHI (purple), Me-DHI (blue), *i*Bu-DHI (red), and 2-EtBu-DHI (green) in deaerated THF ([DHI derivative : $Ir(ppy)_3$] = 1 mM : 50 μM, λ_{ex} = 445 nm, λ_{dt} = 370 nm). Excitation light was removed with a 425 nm short-pass filter. The light gray line is the instrumental response function (IRF) of the instrument. The fitting curves for the tail part of the UC decays were obtained by the known relationship,[5]

$$I_{UC}(t) \propto \exp\left(\frac{-t}{\tau_{UC}}\right) = exp\left(\frac{-2t}{\tau_T}\right) \qquad (1)$$

, where τ_{UC} and τ_T are the UC emission lifetime and acceptor triplet lifetime, respectively.

To optimize triplet energy transfer efficiency (Φ_{TET} > 90%), the UC sample of the concentration of [Me-DHI, *i*Bu-DHI, and 2-EtBu-DHI] : [Ir(ppy)$_3$] = 10 mM : 100 μM were measured (DHI was not dissolved at a concentration of 10 mM).

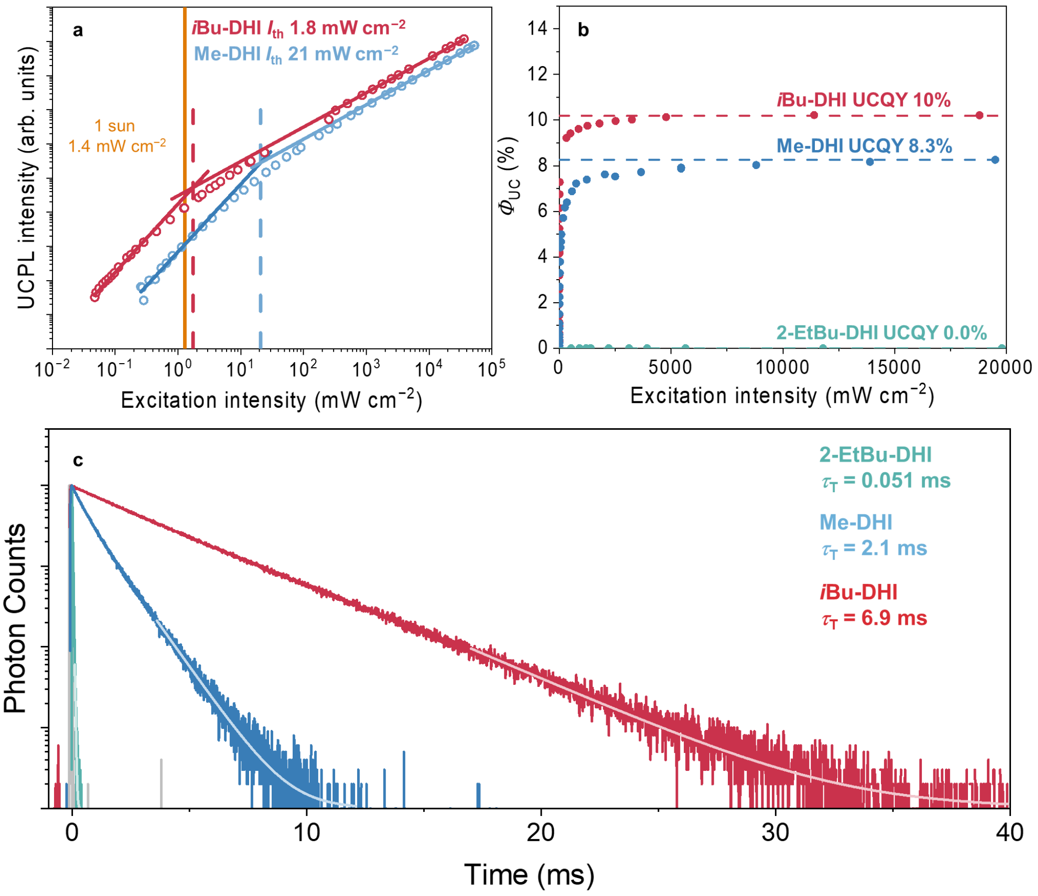

Supplementary Figure 11. Upconversion properties for DHI derivatives. **a** Plots of excitation intensity-dependent UC emission intensity. The slopes obtained from the linear fits transitioned from 2.0 to 1.0 for Me-DHI and *i*Bu-DHI. **b** Plots of Φ_{UC} at various excitation intensities in deaerated THF. The yellow line represents the intensity of light in the 445±5 nm range in sunlight. **c** UC emission decay curves of Me-DHI (blue), *i*Bu-DHI (red), and 2-EtBu-DHI (green) in deaerated THF ([DHI derivative] : [Ir(ppy)$_3$] = 10 mM : 100 μM, λ_{ex} = 445 nm, λ_{dt} = 370 nm). Excitation light was removed with a 425 nm short-pass filter. The fitting curves for the tail part of the UC decays were obtained using the known relationship, given by equation (1).

Supplementary Table 1. Summary of TTA-UC properties in deaerated THF ([Ir(ppy)$_3$] = 50 μM for 1 mM DHI, [Ir(ppy)$_3$] = 100 μM for 10 mM Me-DHI or *i*Bu-DHI, respectively).

Acceptor	Concentration (mM)	Φ_{UC} (%)	I_{th} (mW cm^{-2})	τ_T [a] (ms)	τ_P [b] (μs)	Φ_{TET} [b] (%)	Φ_F [c] (%)	f [d] (%)
DHI	1	3.1	1.5 × 10^2	1.5	0.56	65	88	11
Me-DHI	10	8.3	21	2.1	0.077	95	91	19
*i*Bu-DHI	10	10	1.8	6.9	0.15	91	92	24

[a] Triplet lifetime was obtained from UC decays using the known relationship, given by equation (1).

[b] The TET efficiency Φ_{TET} was estimated by the following equation,

$$\Phi_{TET} = 1 - \frac{\tau_P}{\tau_{P,0}} \quad (2)$$

, where τ_P and $\tau_{P,0}$ represent the phosphorescence lifetime of the donor with and without the acceptor (Figure S8), respectively.

[c] Fluorescence quantum yields were measured at [DHI] = 1 mM and [Me-DHI] = [*i*Bu-DHI] = 10 mM at λ_{ex} = 300 nm, λ_{dt} = 330 nm–470 nm.

[d] f was calculated by the following equation,

$$\Phi_{UC} = \frac{1}{2} f \Phi_{ISC} \Phi_{TET} \Phi_{TTA} \Phi_F \quad (3)$$

, and Φ_{ISC} and Φ_{TTA} were assumed to be 1.

Suppl. Note 6. Photostability of the UC emission in solution

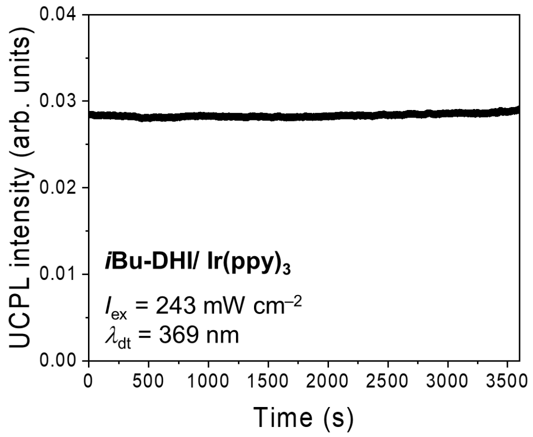

Supplementary Figure 12. Time-dependent UCPL intensity in deoxidized THF ([*i*Bu-DHI] = 10 mM, [Ir(ppy)$_3$] = 100 μM) under continuous laser irradiation for 1 hour (λ_{ex} = 445 nm, λ_{dt} = 369 nm, I_{ex} = 243 mW cm^{-2}).

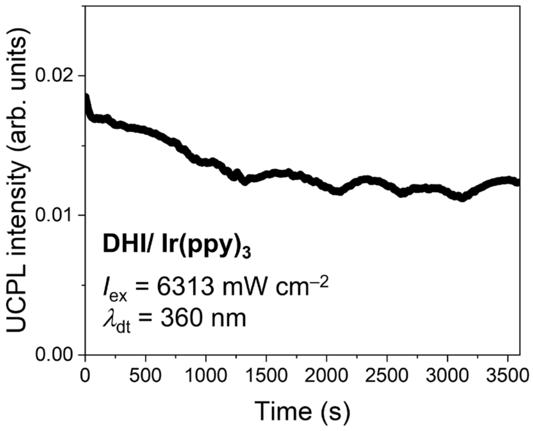

Supplementary Figure 13. Time-dependent UCPL intensity in deaerated THF ([DHI] = 1 mM, [Ir(ppy)$_3$] = 50 μM) under continuous laser irradiation for 1 hour (λ_{ex}= 445 nm, λ_{dt}= 360 nm, I_{ex} = 6.3 W cm^{-2}). We observed a gradual decrease in the UC emission intensity, likely arising from reactions such as photodimerization reported under UV irradiation.[6] To minimize this effect, the spectra were recorded soon after irradiation, with minimal exposure time (< 0.5 s).

Suppl. Note 7. Formation of aggregates in the mixed solution of $Ir(ppy)_3$ and 2-EtBu-DHI

Irradiation at 445 nm to the mixed solution of $Ir(ppy)_3$ and 2-EtBu-DHI resulted in the formation of aggregates. This appears to be the result of an interaction between $Ir(ppy)_3$ and 2-EtBu-DHI as aggregates were not observed upon UV irradiation of the concentrated 2-EtBu-DHI solution. To investigate the interaction between $Ir(ppy)_3$ and 2-EtBu-DHI in the ground state, ^{1}H-NMR spectroscopy was conducted on mixtures of 2-EtBu-DHI and $Ir(ppy)_3$ at molar ratios of 10:1, 20:1, and 100:1 (Figure S14). The 20:1 and 100:1 conditions are identical to those used for the UC measurements (Figure S9–11). The ^{1}H-NMR spectra of all samples showed the same pattern as 2-Et-Bu-DHI, suggesting that aggregation-induced species are not present. After laser exposure, a white precipitate formed in the cuvette (Figure S15b), indicating that the aggregation is a photo-induced event that occurs exclusively during laser excitation.

To evaluate whether aggregation occurs in other solvents, the sample in toluene was also tested (Figure S15a). Similar aggregation was observed in the toluene solution, suggesting that the interaction between $Ir(ppy)_3$ and 2-EtBu-DHI is not limited to a specific solvent environment. This indicates that the aggregation behavior is likely driven by intrinsic molecular interactions rather than by solvent-specific effects. However, the mechanism remains to be determined. Further investigation is required to clarify the origin of this phenomenon.

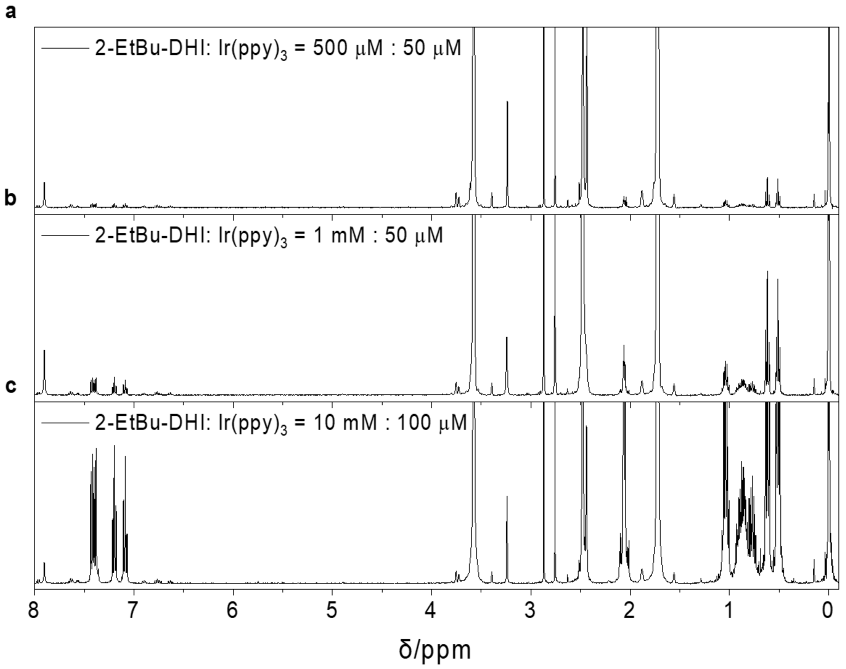

Supplementary Figure 14. ^{1}H-NMR (400 MHz, THF-*D8*, TMS) spectra of 2-EtBu-DHI and Ir(ppy)$_3$ mixtures at varying concentrations (**a** 500 μM: 50 μM mixture, **b** 1 mM: 50 μM mixture, and **c** 10 mM: 100 μM mixture).

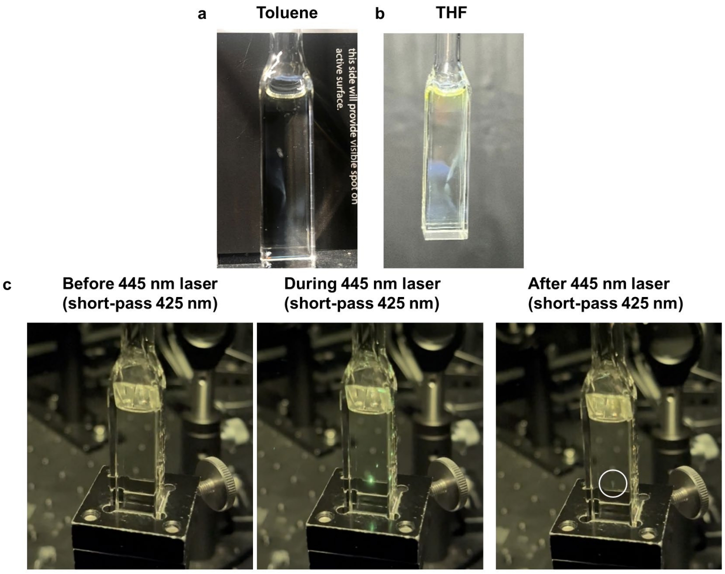

Supplementary Figure 15. Formation of aggregates in the mixed solution of Ir(ppy)$_3$ and 2-EtBu-DHI after irradiation with a 445 nm laser. **a**, **b** Photographs illustrating the influence of the solvent environment on the aggregate formation in toluene **a** and **b** THF. **c** Solvent-dependent aggregation behavior and photo-induced assembly of 2-EtBu-DHI with Ir(ppy)$_3$. The concentrations of 2-EtBu-DHI and Ir(ppy)$_3$ were 10 mM and 100 μM, respectively.

Suppl. Note 8. UC profiles in solid states

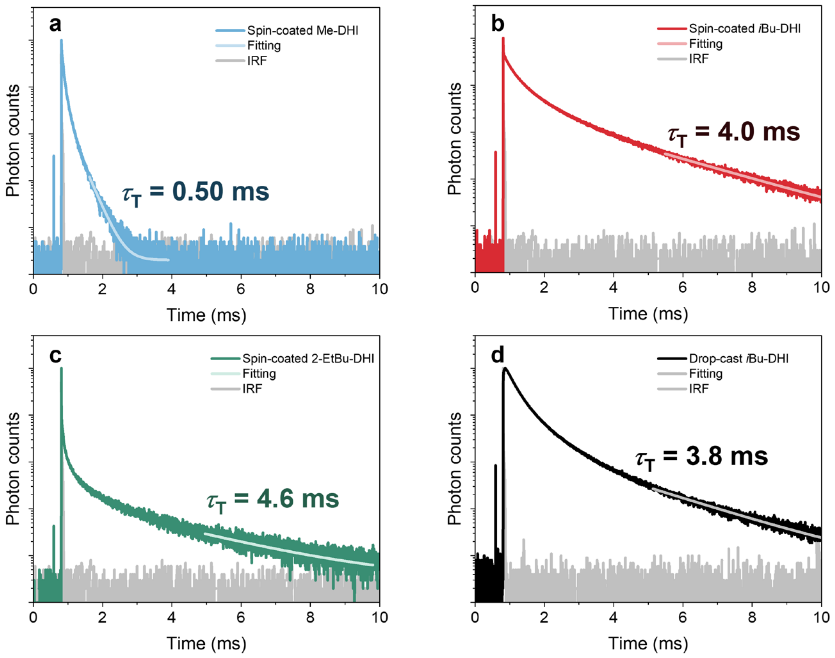

Supplementary Figure 16. Time-resolved UC emission decay profiles in thin films of DHI derivatives. Decay profiles of spin-coated films of **a** Me-DHI, **b** *i*Bu-DHI, **c** 2-EtBu-DHI, and the drop-cast film of **d** *i*Bu-DHI doped with $Ir(ppy)_3$ (molar ratio for preparation, DHI derivatives : $Ir(ppy)_3$ = 100 : 1). Decay profiles were recorded upon excitation at 445 nm, with detection at 370 nm. The light gray line is the instrumental response function (IRF) of the instrument. The fitting curves for the tail part of the UC decays were obtained using the known relationship described by equation (1).

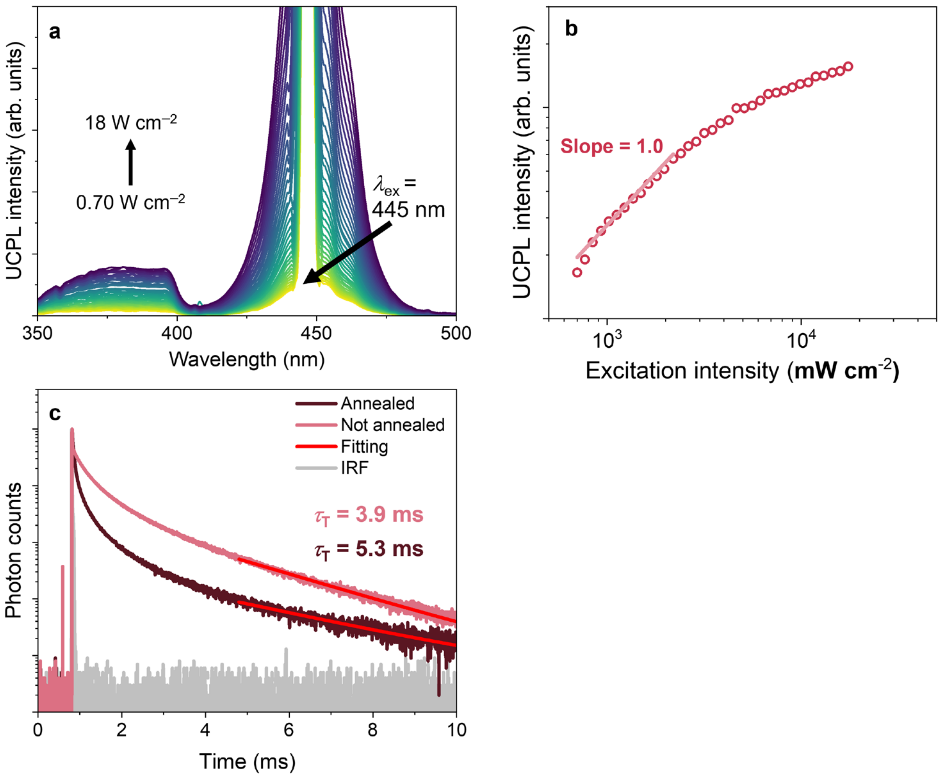

Supplementary Figure 17. Upconversion properties of the spin-coated film of *i*Bu-DHI (T_m ~ 110 °C) doped with $Ir(ppy)_3$ after annealing at 120 °C. **a** UC emission spectrum and **b** excitation intensity dependence of the upconverted emission intensity. Excitation light was removed with a 400 nm short-pass filter. **c** UC emission decay curves of the *i*Bu-DHI UC film before and after annealing.

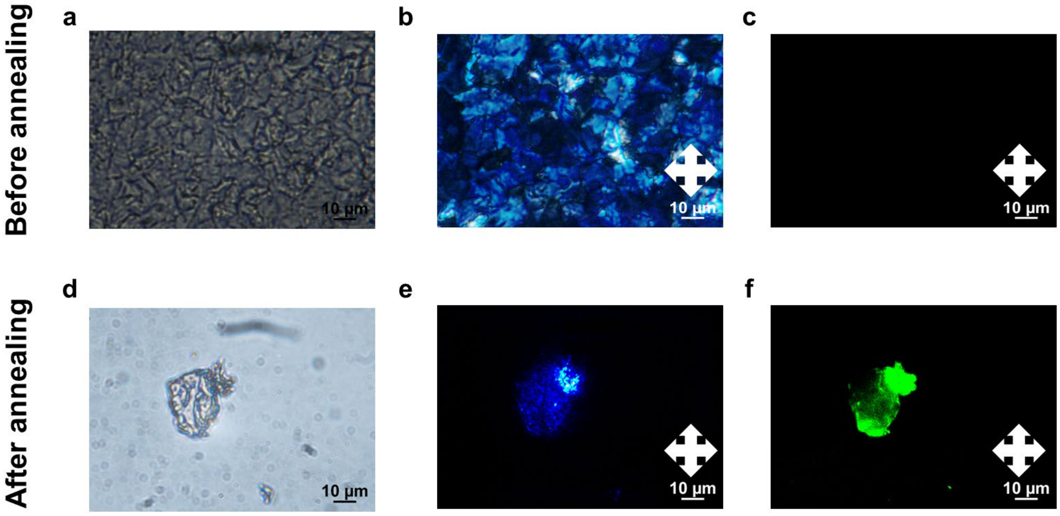

Supplementary Figure 18. Optical and phosphorescence microscopy images of spin-coated *i*Bu-DHI (T_m ~ 110 °C) doped with Ir(ppy)$_3$ films before and after thermal annealing. **a–c** before and **d–f** after annealing at 120 °C. Images were taken without (**a,d**) and with (**b,e**) crossed nicoles. The cross-polarized images under broad band excitation (450–490 nm) with 505 nm long-pass filters to detect Ir(ppy)$_3$ phosphorescence.

Suppl. Note 9. Fluorescence decay profiles in solid states

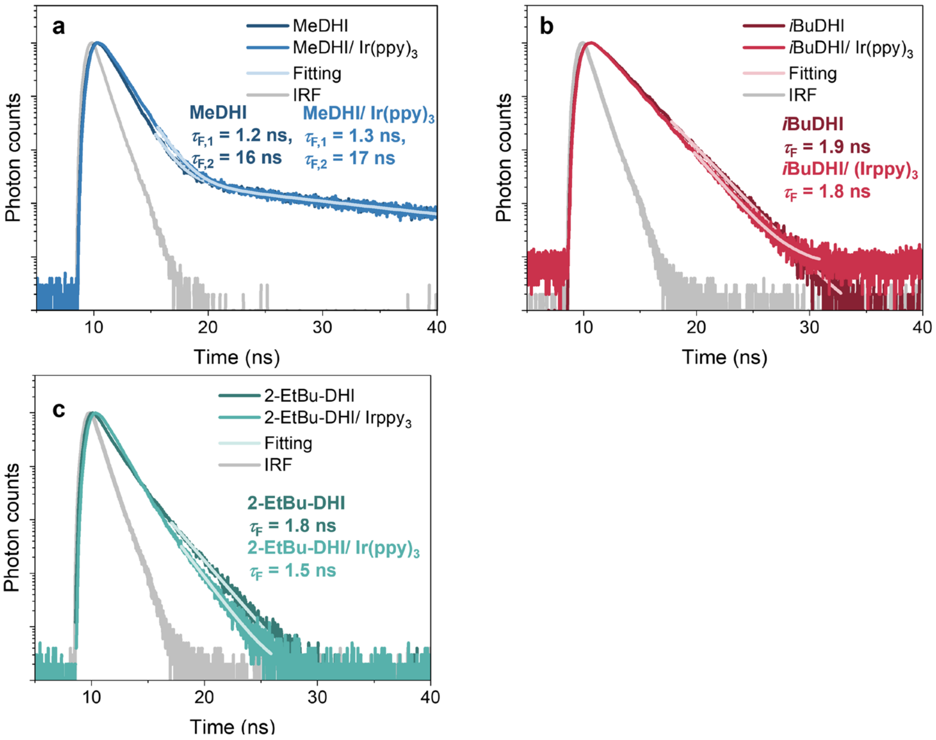

Supplementary Figure 19. Fluorescence decay kinetics of spin-coated films of DHI derivative (**a** Me-DHI, **b** *i*Bu-DHI, and **c** 2-EtBu-DHI) with and without $Ir(ppy)_3$ (molar ratio for preparation; DHI derivative: $Ir(ppy)_3$ = 100 : 1, λ_{ex} = 370 nm). The decay of Me-DHI was fitted with a bi-exponential function, and those for *i*Bu-DHI and 2-EtBu-DHI were fitted with a mono-exponential function. The light gray line represents the instrumental response function (IRF) of the instrument.

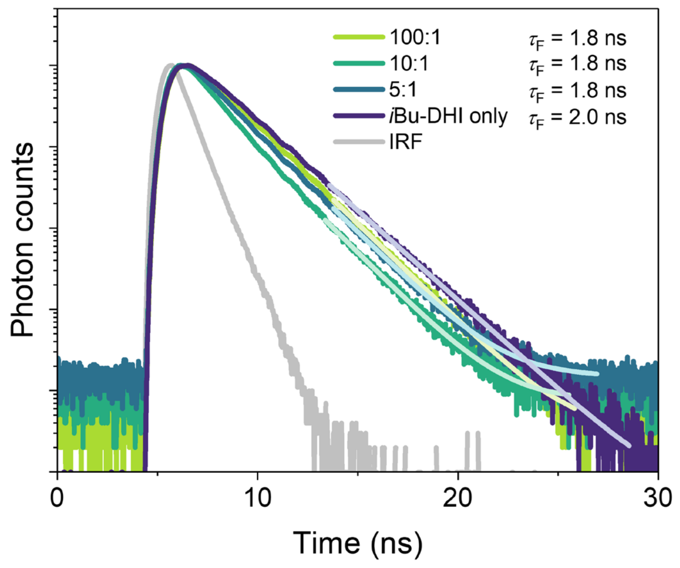

Supplementary Figure 20. Fluorescence decay profiles of spin-coated films of *i*Bu-DHI with Ir(ppy)$_3$ at various acceptor/donor ratios. The decays were fitted with a mono-exponential function. The light gray line is the instrumental response function (IRF) of the instrument.

Suppl. Note 10. Self-absorption correction of an absolute UC quantum yield

The absolute UC quantum yield of the spin-coated film of Ir(ppy)$_3$ and *i*Bu-DHI was estimated based on the protocol by N. Yanai et al.[7] In short, the TTA-UC emission spectra with (A) and without (B) integrating sphere were obtained by mechanically moving the sphere without changing any other optics. These spectra were normalized by the tail of the phosphorescence of Ir(ppy)$_3$ (Figure S21b). The reabsorption probability (a) was obtained by the following relationship

$$\frac{\int_{\lambda 1}^{\lambda 2} P_{\mathrm{A}}(\lambda) d\lambda}{\int_{\lambda 1}^{\lambda 2} P_{\mathrm{B}}(\lambda) d\lambda} = 1 - a \tag{4}$$

P is the emitted photons. The value of *i*Bu-DHI/ Ir(ppy)$_3$ was obtained for the integration from 350 nm to 405 nm, and the Φ_{UC} was estimated by the equation below.

$$\Phi_{\mathrm{UC,obs}} = \Phi_{\mathrm{UC}}(1 - a) \tag{5}$$

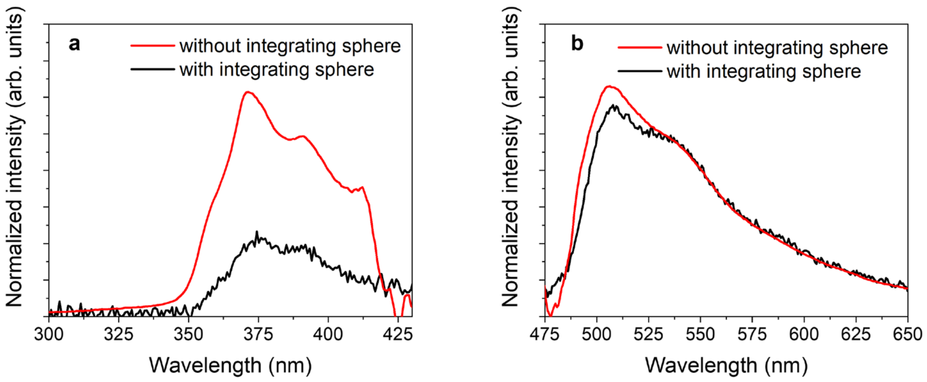

Supplementary Figure 21. Comparison of UC emission and phosphorescence spectra with and without an integrating sphere. **a** Normalized UC emission and **b** phosphorescence spectra of the spin-coated film of *i*Bu-DHI doped with Ir(ppy)$_3$ detected with (black) and without (red) integrating sphere (I_{ex} = 4260 mW cm^{-2}). The spectra were normalized to the intensity at 591 nm.

Suppl. Note 11. Scanning electron microscopy with energy dispersive X-ray spectroscopy (SEM-EDX)

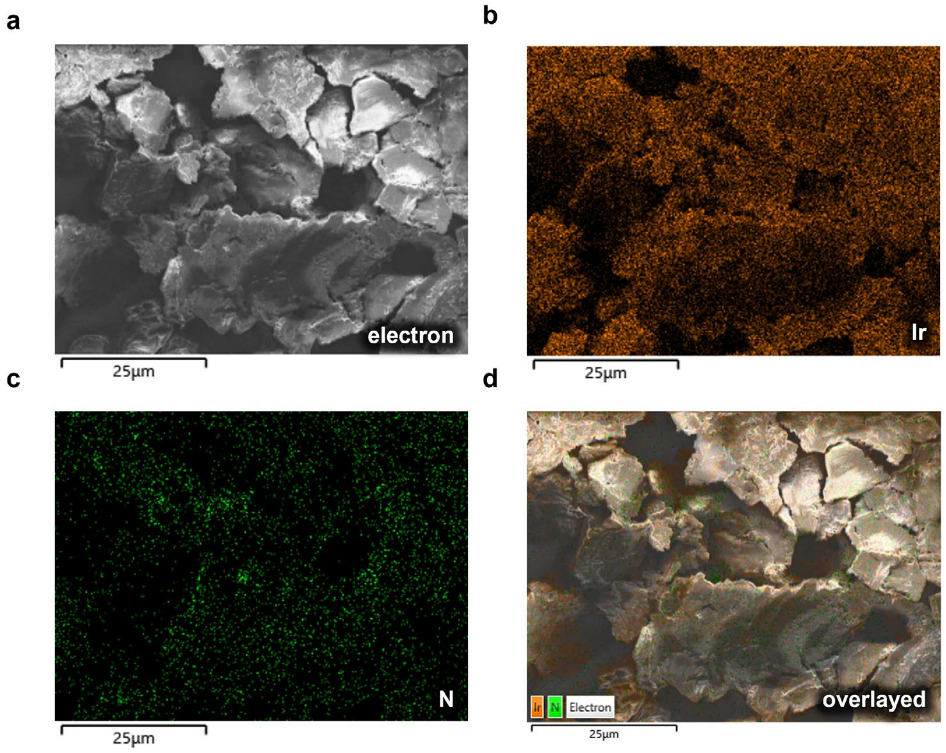

Supplementary Figure 22. Surface morphology and elemental distribution of 2-EtBu-DHI drop-cast films. **a** SEM image and SEM-EDX mappings of **b** Ir, **c** N, and **d** their overlay for the drop-cast film of 2-EtBu-DHI with Ir(ppy)$_3$.

Suppl. Note 12. Phosphorescence decay profiles and quantum yield in solids

The decay profile was fitted using a multi-exponential function of the form:

$$I(t) = \sum_{i=1}^{n} A_i \exp\left(-\frac{t}{\tau_i}\right) \quad (6)$$

, where I(t) is the phosphorescence intensity at time t, A_i represents the pre-exponential factor of each decay component, and τ_i is the corresponding photoluminescence lifetime. The amplitudes reflect the relative contribution of each decay component to the overall signal. The intensity-weighted average lifetime $\langle\tau\rangle$ and fractional contribution (f_i) were calculated using the following equations:

$$\langle\tau\rangle = \frac{\sum_{i=1}^{n} A_i {\tau_i}^2}{\sum_{i=1}^{n} A_i \tau_i} \quad (7)$$

$$f_i = \frac{A_i \tau_i}{\sum_{i=1}^{n} A_i \tau_i} \quad (8)$$

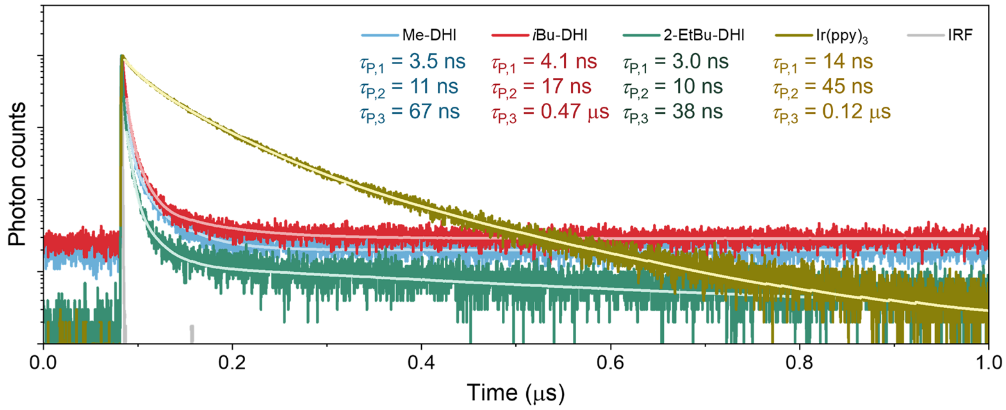

Supplementary Figure 23. Time-resolved phosphorescence decay curves of the spin-coated films of $Ir(ppy)_3$ in the presence of Me-DHI (blue), *i*Bu-DHI (red), and 2-EtBu-DHI (green), along with a reference drop-cast film of $Ir(ppy)_3$ alone (yellow). Decay profiles were recorded upon excitation at 405 nm, with detection at 515 nm. The instrumental response function (IRF) is also shown (gray).

Supplementary Table 2. Phosphorescence lifetime parameters obtained from multi-exponential fitting of the time-resolved decay profile of the spin-coated films of Ir(ppy)$_3$ in the presence of Me-DHI, *i*Bu-DHI, and 2-EtBu-DHI, along with a reference drop-cast film of Ir(ppy)$_3$ alone. Decay profiles were recorded upon excitation at 405 nm, with detection at 515 nm. The table summarizes the fitted lifetimes (τ_i), corresponding pre-exponential factor (A_i), fractional intensity (f_i), and the calculated average lifetime $\langle\tau\rangle$ from equations (6–8).

	$\langle\tau\rangle$ (ns)	τ_1 (ns)	τ_2 (ns)	τ_3 (ns)	A_1	A_2	A_3	f_1 (%)	f_2 (%)	f_3 (%)
Ir(ppy)$_3$	58.7	14.3	45.1	121	846.9	1006	153.1	16	60	24
Ir(ppy)$_3$/ Me-DHI	11.6	3.52	11.1	66.9	1722	244.8	13.49	63	28	9
Ir(ppy)$_3$/ *i*Bu-DHI	136	4.09	17.0	474	585.0	31.33	2.363	59	13	28
Ir(ppy)$_3$/ 2-EtBu-DHI	8.94	2.99	10.3	37.6	1597	150.8	24.40	66	21	13

The TET efficiency (Φ_{TET}) in the solid state was evaluated by the drop of phosphorescence quantum yield (Φ_P) using the following equation.

$$\Phi_{TET} = 1 - \frac{\Phi_P}{\Phi_{P,0}} \quad (9)$$

As we cannot deny the presence of nanoscale segregation, we used the neat solid of Ir(ppy)$_3$ as a reference to obtain the lower limit.

Supplementary Table 3. Phosphorescence quantum yield and TET efficiency of the neat solid of Ir(ppy)$_3$ and spin-coated DHI films with Ir(ppy)$_3$.

**Film**	Φ_P (%)	Φ_{TET} (%)
Ir(ppy)$_3$	9.4	-
Ir(ppy)$_3$/Me-DHI	3.9	59
Ir(ppy)$_3$/*i*Bu-DHI	2.7	71
Ir(ppy)$_3$/2-EtBu-DHI	0.63	93

When the phosphorescence quantum yield of the THF solution of Ir(ppy)$_3$ (100 μM) was used as a reference ($\Phi_{P,0}$ = 88%), Φ_{TET} values of Me-DHI, *i*Bu-DHI, and 2-EtBu-DHI UC films are calculated as 96%, 97%, and 99%, respectively. In either case, the difference in TET efficiency between *i*Bu-DHI and 2-EtBu-DHI does not account for the 3 orders of magnitude difference in I_{th}, indicating the contribution of triplet diffusion in the crystal discussed in section 16.

Suppl. Note 13. UC emission from heavy metal-free sensitizer-doped films

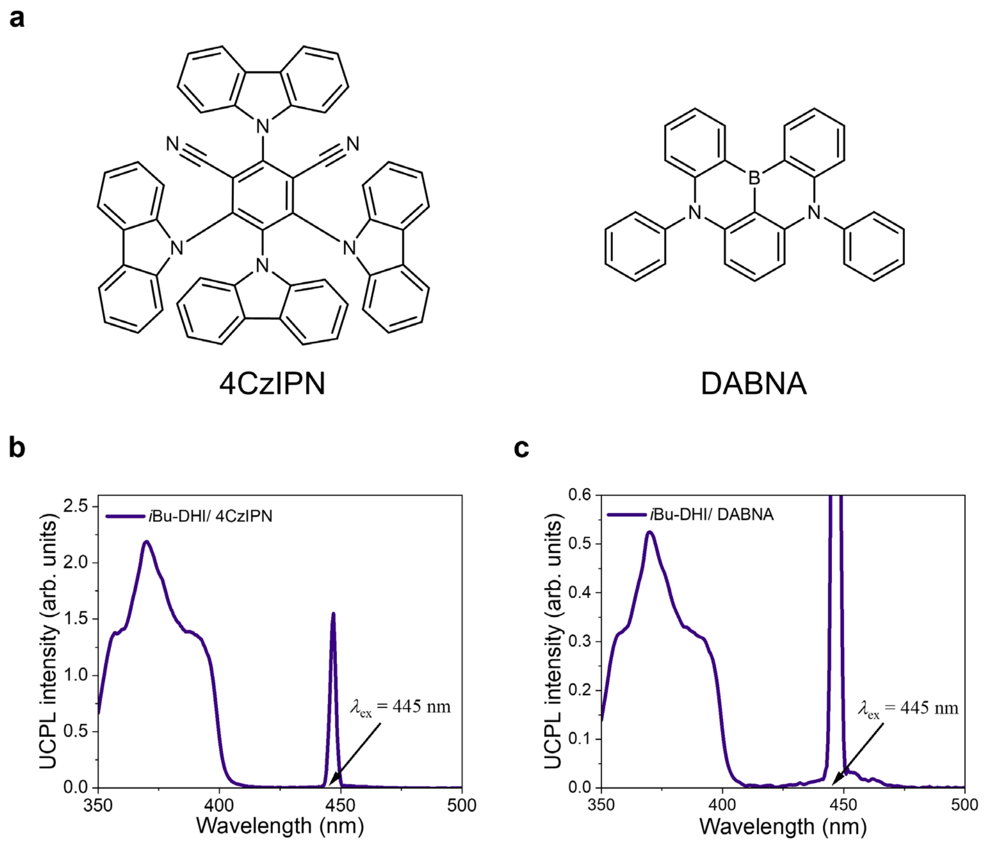

Supplementary Figure 24. Chemical structures and TTA-UC emission spectra for donor-acceptor thin-film systems. **a** Chemical structure of 4CzIPN and DABNA. TTA-UC emission spectra of spin-coated films of **b** *i*Bu-DHI/4CzIPN and **c** *i*Bu-DHI/DABNA under excitation at 445 nm (molar ratio for preparation; *i*Bu-DHI : donor = 100 : 1, I_{ex} = 138 mW cm^{-2}). Excitation light was removed with a 400 nm short-pass filter.

Suppl. Note 14. Evaluation of oxygen tolerance and stability of TTA-UC

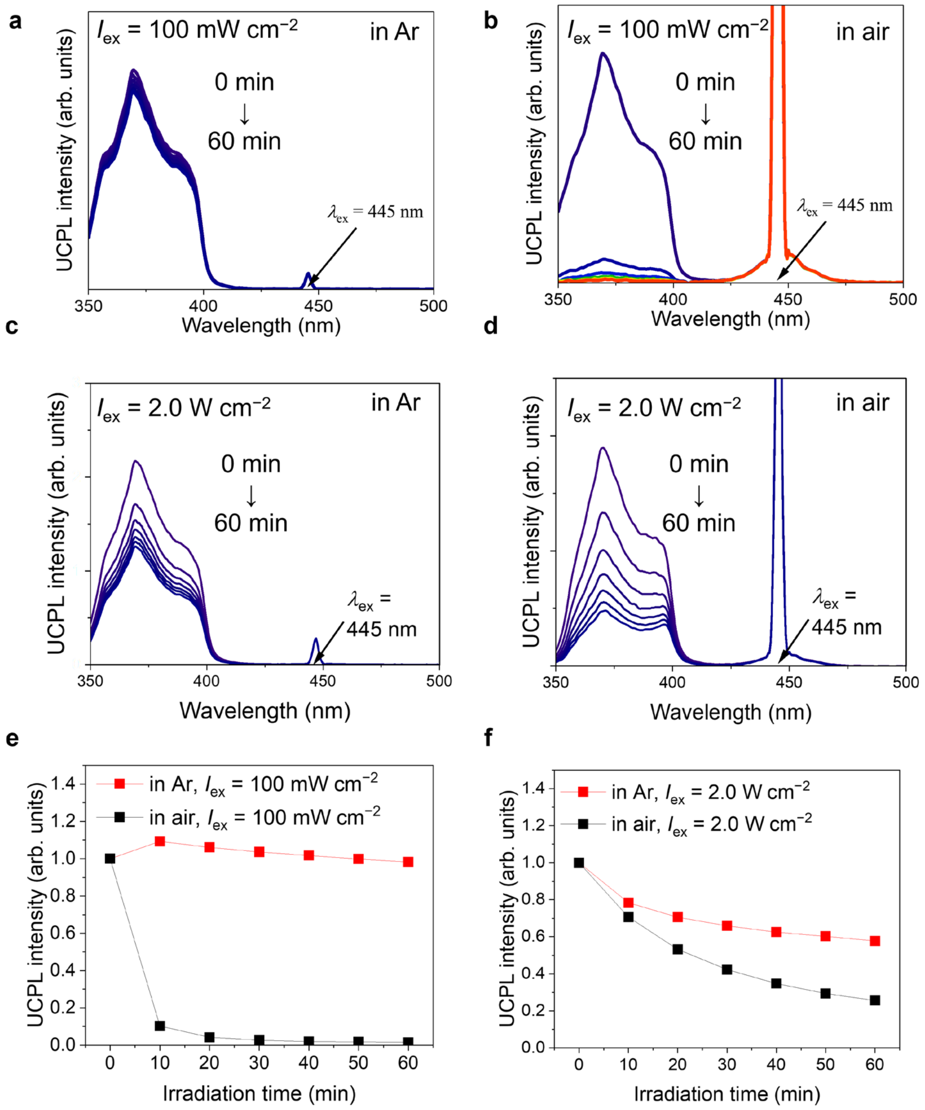

Supplementary Figure 25. Stability of TTA-UC emission in *i*Bu-DHI/Ir(ppy)$_3$ films in Ar and air. Time-dependent UC emission spectra of a spin-coated film of *i*Bu-DHI/Ir(ppy)$_3$ (molar ratio for preparation; *i*Bu-DHI : Ir(ppy)$_3$ = 100 : 1) prepared and measured **a** in Ar and **b** in air were recorded under excitation at 100 mW cm^{-2}. The results **c** in Ar and **d** in air were recorded under 2.0 W cm^{-2}. Excitation light was removed with a 400 nm short-pass filter. Time-dependent UC emission intensity with (red) and without (black) encapsulation under excitation intensity **e** at 100 mW cm^{-2} and **f** at 2.0 W cm^{-2} (λ_{dt} = 370 nm). It was difficult to evaluate Φ_{UC} and I_{th} in air at t = 0 min under air-saturated conditions because the weak UCPL intensity required approximately 1 minute of exposure time.

Suppl. Note 15. Crystal structures of DHI derivatives

Supplementary Table 4. Crystal data and structure refinement for Me-DHI.

Empirical formula	$C_{10}H_{10}$
Formula weight	130.18
Temperature (K)	100.00(10)
Crystal system	Orthorhombic
Space group	*Pbca*
a (Å)	11.1817(4)
b (Å)	8.6973(2)
c (Å)	15.0291(5)
Alpha (˚)	90
Beta (˚)	90
Gamma (˚)	90
Volume (Å^3)	1461.59(8)
Z	8
ρ_{calc}g (cm^3)	1.183
μ (mm^{-1})	0.496
F(000)	560.0
Crystal size (mm^3)	0.382 × 0.228 × 0.123
Reflections collected	4543
Goodness of Fit on F^2	1.036
Final R indices [I >= 2σ(I)]	R_1 = 0.0387, wR_2 = 0.1015
Final R indices [all data]	R_1 = 0.0402, wR_2 = 0.1031
Largest diff. peak/hole (e Å^{-3})	0.33/–0.19

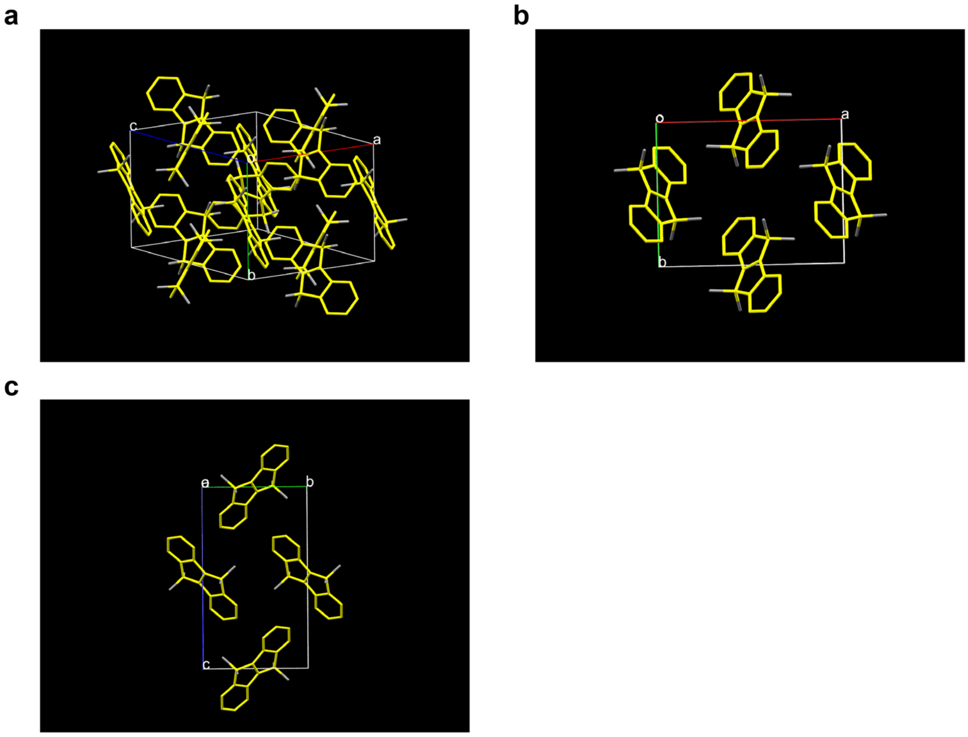

Supplementary Figure 26. Crystal structure of Me-DHI. **a** A diagonal view of the unit cell from above, **b** the packing on the ab plane, and **c** on the bc plane.

Supplementary Table 5. Crystal data and structure refinement for *i*Bu-DHI.

Empirical formula	$C_{32}H_{44}$
Formula weight	428.67
Temperature (K)	100.00(10)
Crystal system	Monoclinic
Space group	$P2_1/c$
a (Å)	9.7383(2)
b (Å)	16.2631(4)
c (Å)	17.2873(4)
Alpha (˚)	90
Beta (˚)	104.864(3)
Gamma (˚)	90
Volume (Å^3)	2646.27(11)
Z	4
ρ_{calc}g (cm^3)	1.076
μ (mm^{-1})	0.439
F(000)	944.0
Crystal size (mm^3)	0.29 × 0.182 × 0.054
Reflections collected	16973
Goodness of Fit on F^2	1.052
Final R indices [I >= 2σ(I)]	R_1 = 0.0454, wR_2 = 0.1216
Final R indices [all data]	R_1 = 0.0504, wR_2 = 0.1263
Largest diff. peak/hole (e Å^{-3})	0.47/–0.31

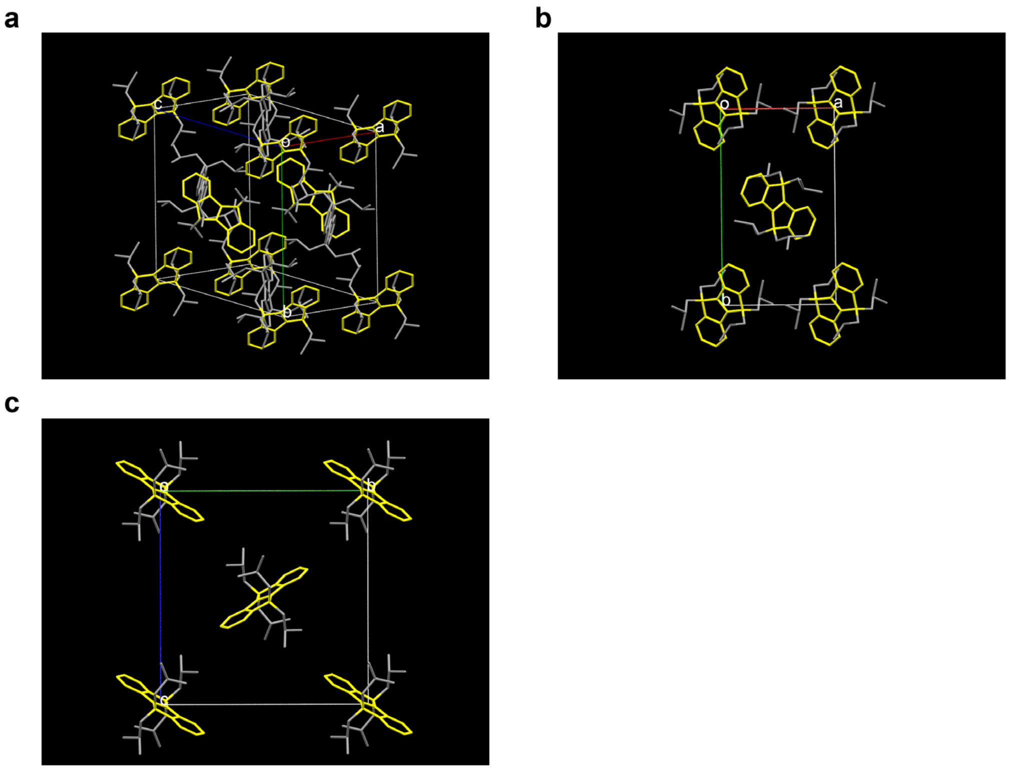

Supplementary Figure 27. Crystal structure of *i*Bu-DHI. **a** A diagonal view of the unit cell from above, **b** the packing on the ab plane, and **c** on the bc plane**.**

Supplementary Table 6. Crystal data and structure refinement for 2-EtBu-DHI.

Empirical formula	$C_{20}H_{30}$
Formula weight	270.44
Temperature (K)	100.00(10)
Crystal system	Monoclinic
Space group	*C*2/c
a (Å)	13.8586(4)
b (Å)	14.0026(4)
c (Å)	17.6924(5)
Alpha (˚)	90
Beta (˚)	108.770(3)
Gamma (˚)	90
Volume (Å^3)	3250.73(17)
Z	8
ρ_{calc}g (cm^3)	1.105
μ (mm^{-1})	0.447
F(000)	1200.0
Crystal size (mm^3)	0.2 × 0.112 × 0.106
Reflections collected	12582
Goodness of Fit on F^2	1.080
Final R indices [I >= 2σ(I)]	R_1 = 0.0413, wR_2 = 0.1145
Final R indices [all data]	R_1 = 0.0450, wR_2 = 0.1185
Largest diff. peak/hole (e Å^{-3})	0.26/–0.20

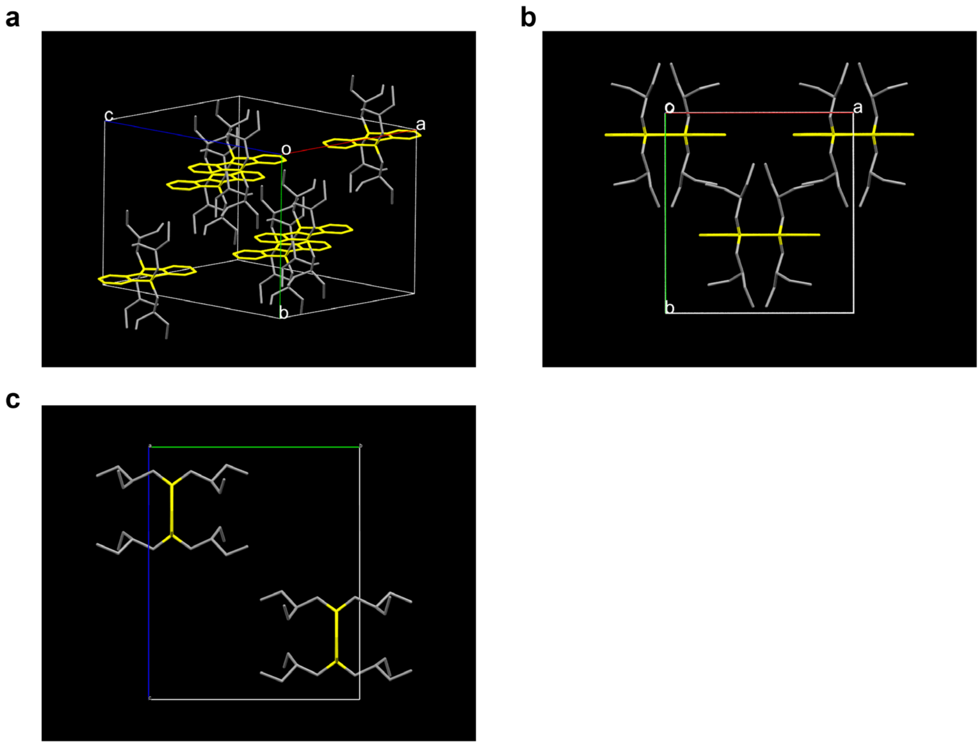

Supplementary Figure 28. Crystal structure of 2-EtBu-DHI. **a** A diagonal view of the unit cell from above, **b** the packing on the ab plane, and **c** on the bc plane.

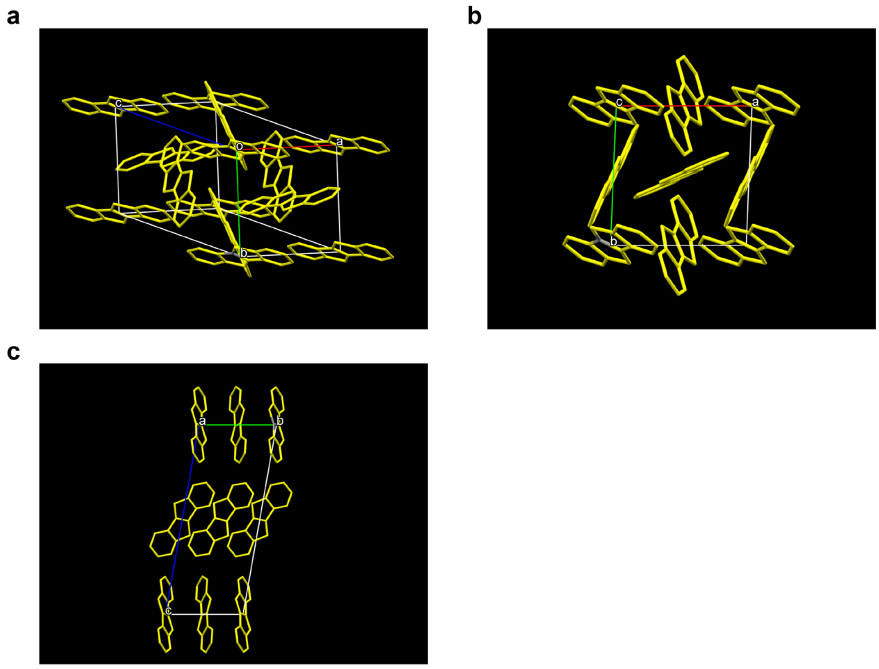

Supplementary Figure 29. Reported crystal structure of DHI (CSD deposition number: 1949606).[8] **a** A diagonal view of the unit cell from above, **b** the packing on the ab plane, and **c** on the bc plane.

Suppl. Note 16. Theoretical analysis of triplet energy transfer and triplet–triplet annihilation reaction times in crystals

To elucidate the triplet–triplet annihilation (TTA) and triplet energy transfer (TET) of Me-DHI, *i*Bu-DHI, and 2-EtBu-DHI in the crystal, the electronic coupling matrix elements ($|T_{DA}|$) and reaction times (τ) of TTA and TET were examined by the ab initio and DFT/TDDFT calculations. The details of the calculations are described below, which were referred to those in previous works.[9–12] The results of TTA and TET were summarized in Table S7 and Figure S30.

The TET is a key process for the TTA-UC. For Me-DHI, pair ***b*** shows $|T_{DA}^{TET}|$ and τ_{TET} values of 0.0836 meV and 5.27 μs (Table S7). In the case of *i*Bu-DHI, the ***b*** pathway has relevant $|T_{DA}^{TET}|$ and τ_{TET} values as 0.157 meV and 1.25 μs. For 2-EtBu-DHI, the TET is inferior to the other two compounds, and the $|T_{DA}^{TET}|$ and τ_{TET} values for possible TET paths are 0.0009 meV and 4.20×10^4 μs (pair ***a***) or 0.0011 meV and 2.85×10^4 μs (pair ***b***). These results are in good agreement with the experimental observation that the upconversion of *i*Bu-DHI is better than that of the other two molecules.

The notable $|T_{DA}^{TTA}|$ and τ_{TTA} values for TTA are in the order of *i*Bu-DHI ≈ Me-DHI ≫ 2-EtBu-DHI. This mainly originates from the distance between the interacting π-conjugated systems of the pair of chromophores in the crystal structure. The calculated relaxation energies were nearly the same for the three compounds. The $|T_{DA}^{TTA}|$ value is large for pair ***b*** of *i*Bu-DHI as 1.20 meV, and corresponding τ_{TTA} value is 4.54 ns, while those of pair ***b*** for Me-DHI are 1.49 meV and 6.17 ns. The values for pair ***a*** of 2-EtBu-DHI are 0.280 meV and 80.9 ns. Other pairs are less efficient for TTA according to the calculated values.

Supplementary Table 7. Electronic coupling matrix elements ($|T_{DA}|$) and reaction times (τ) of TET and TTA for Me-DHI, *i*Bu-DHI, and 2-EtBu-DHI.

Molecules	pair	$\lvert T_{DA}^{TET} \rvert$ (meV)	τ_{TET} (μs)	$\lvert T_{DA}^{TTA} \rvert$ (meV)	τ_{TTA} (ns)	d [a] (Å)
Me-DHI	***a***	0.0242	6.29×10	0.0119	9.67×10^4	4.444
	b	0.0836	5.27	1.4892	6.17	3.830
	c	0.0002	7.39×10^5	0.0027	1.89×10^6	4.304
*i*Bu-DHI	***a***	0.0026	4.42×10^3	0.0236	1.18×10^4	5.799
	b	0.1570	1.25	1.2038	4.54	3.756
	c	0.0012	2.27×10^4	0.0099	6.73×10^4	6.208
	d	0.0001	3.35×10^6	0.0033	6.08×10^5	7.313
2-EtBu-DHI	***a***	0.0009	4.20×10^4	0.2802	8.09×10	7.043
	b	0.0011	2.85×10^4	0.0024	1.10×10^6	6.800
	c	0.00004	1.91×10^7	0.0015	3.01×10^6	5.093
	d	0.0005	1.52×10^5	0.0061	1.73×10^5	6.014

[a] Nearest C–C distance between pair of π-conjugation of pair molecules.

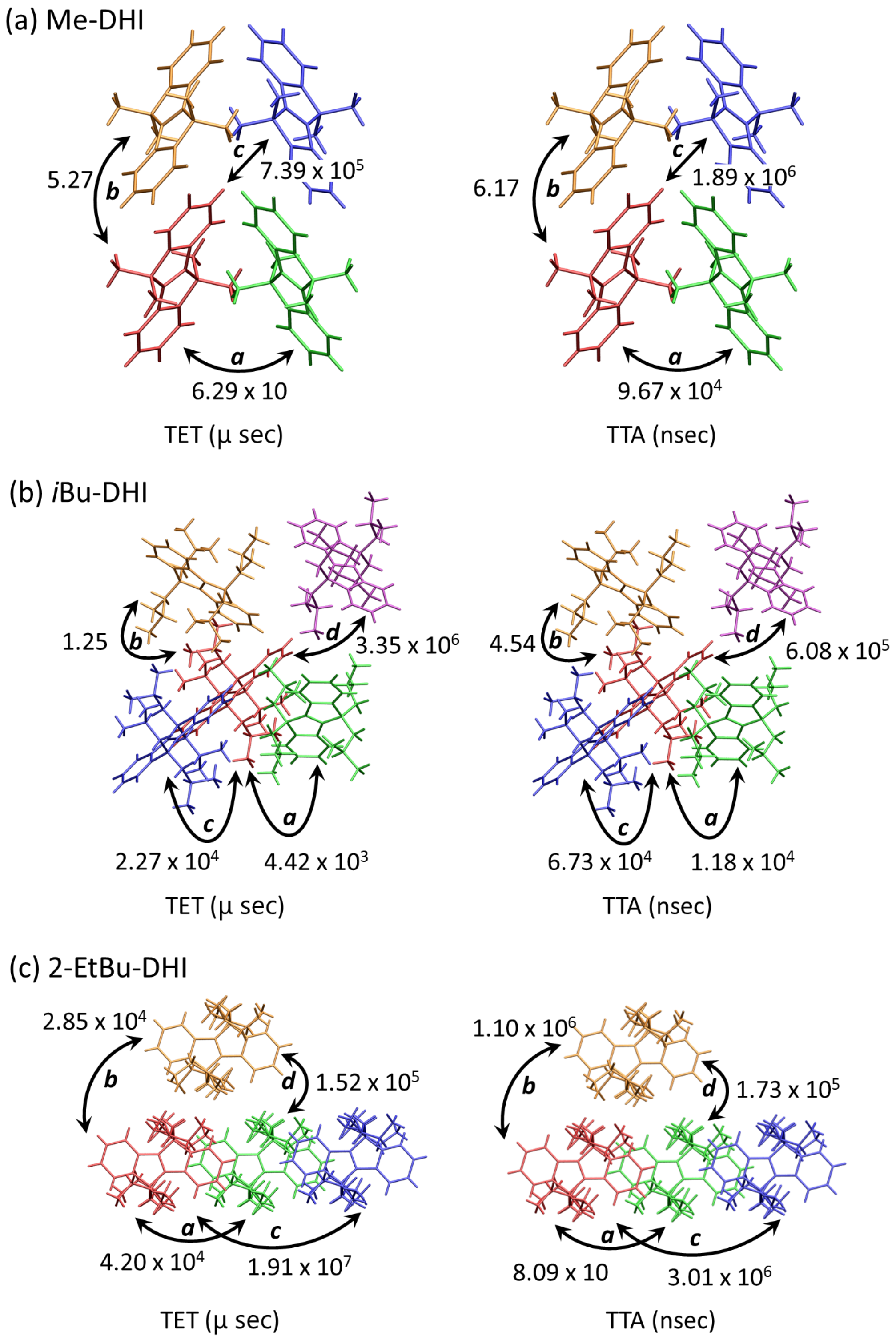

Supplementary Figure 30. Calculated reaction times of TTA (τ_{TTA}) and TET (τ_{TET}) in DHI derivatives (**a** Me-DHI, **b** *i*Bu-DHI, and **c** 2-EtBu-DHI).

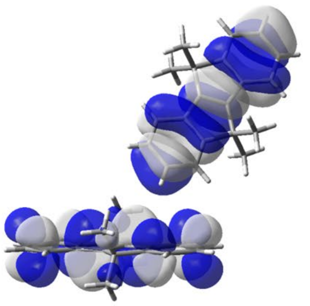

Me-DHI (pair ***b***) (HOMO−1, LUMO+1)

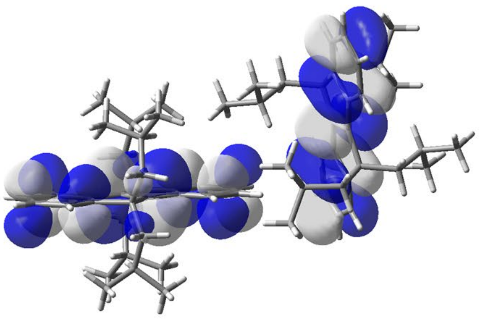

*i*Bu-DHI (pair ***b***) (HOMO−1, LUMO+1)

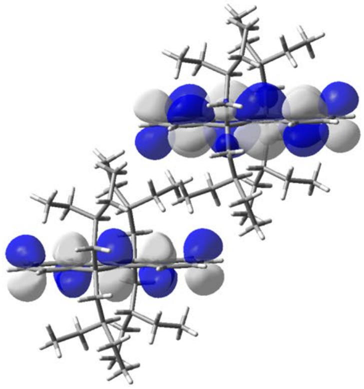

2-EtBu-DHI (pair ***a***) (HOMO−1, LUMO+1)

Supplementary Figure 31. The interaction orbitals of the relevant pairs or pathways, HOMO−1 and LUMO+1, for pairs of Me-DHI (pair ***b***), *i*Bu-DHI (pair ***b***), and 2-EtBu-DHI (pair ***a***) in Figure S30. Iso-surface value is 0.02.

Computational details

The rate constants for TTA and TET were calculated based on the Marcus theory as in the previous works.[9–11]

$$k = \frac{2\pi}{\hbar}|T_{\mathrm{DA}}|^2 \frac{1}{\sqrt{4\pi\lambda k_B T}} exp\left[-\frac{(\Delta G+\lambda)^2}{4\lambda k_B T}\right] \tag{10}$$

where the T_{DA}, λ, ΔG are electronic coupling matrix elements, relaxation energy, and free energy change by TTA/TET, respectively. k_{B} and $\hbar$ are the Boltzmann constant and the Planck constant, respectively, and T is set to 298.15 K.

The $T_{\mathrm{DA}}^{\mathrm{TTA}}$ of TTA-UC were calculated by the perturbative equation,[10,11]

$$T_{\mathrm{DA}}^{\mathrm{TTA}} = \langle T_1T_1|V|S_1S_0\rangle - 2\frac{\langle T_1T_1|H|AC\rangle\langle AC|H|S_1S_0\rangle}{E_{AC}-E_{\mathrm{av}}} \tag{11}$$

where E_{AC} and E_{av} are the charge transfer excitation energies and the averaged values of triplet–triplet excitation energy (T_1T_1) and singlet excitation energy (S_1). The matrix elements were calculated by the equations given in ref. 9.

The $T_{\mathrm{DA}}^{\mathrm{TET}}$ of TET were approximately evaluated by the reported method assuming the Dexter mechanism,[12]

$$T_{\mathrm{DA}}^{\mathrm{TET}} = -[L_D L_A|H_D H_A] \tag{12}$$

where H and L denote HOMO and LUMO, respectively.

The relaxation energies and excitation energies were evaluated using the DFT and TD-DFT calculations. The relaxation energies for TET were calculated by ΔSCF (self-consistent field) with DFT. The B3LYP functional[13] with the 6-311+G(2d,1p) basis sets[14] was adopted for the DFT/TD-DFT calculations. The matrix elements in the equations of the molecular pairs were calculated from the four orbitals constituted of monomer HOMO and LUMO by the Hartree-Fock calculations with 6-311G(d) basis sets. The interaction orbitals of the relevant pairs or pathways are shown in Figure S31. In particular, the diffuse functions caused problems in evaluating molecular integrals due to the extended orbital shape of the LUMO and therefore, we adopted the 6-311G(d) basis set.

All the calculations were conducted using the modified module of the Gaussian 16 version suite of programs, Rev. A.03.[15]

Suppl. Note 17. Reported solid-state TTA-UC properties

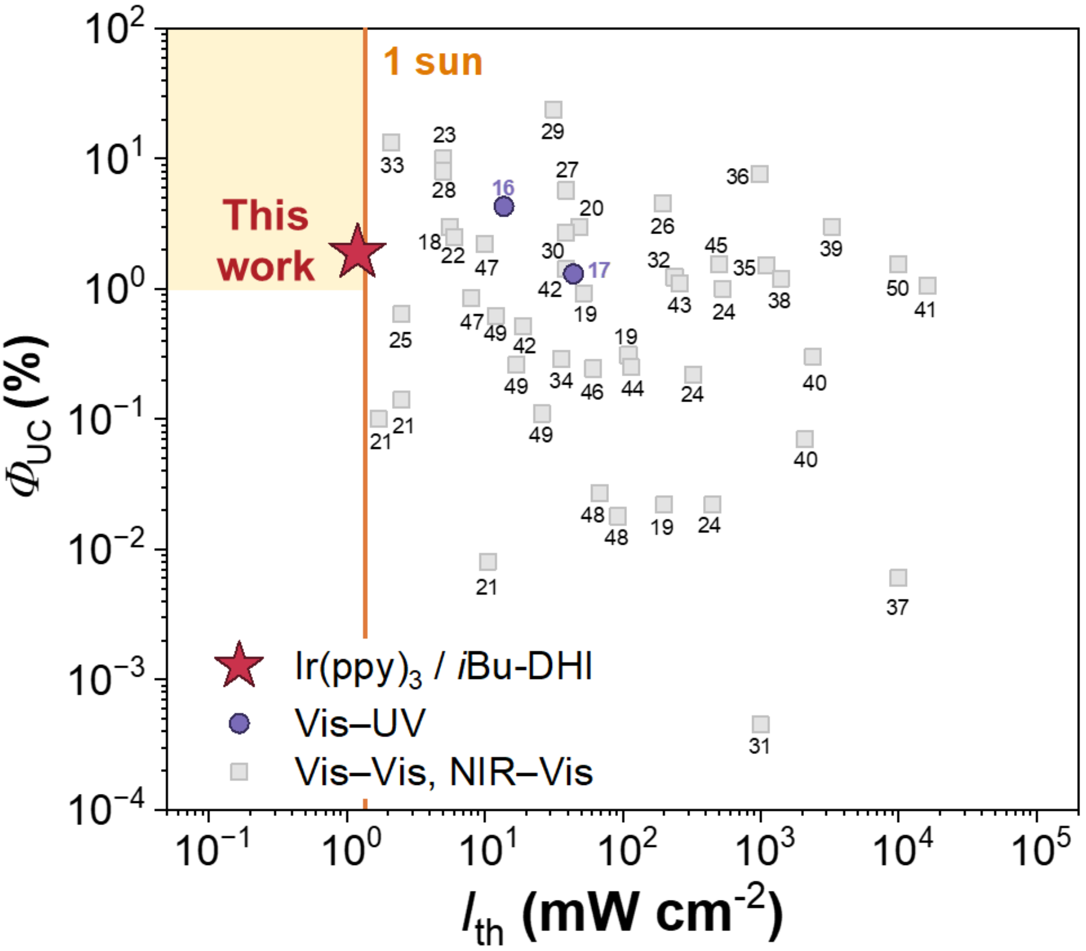

Supplementary Figure 32. Φ_{UC}–I_{th} plots with the reported values of solid TTA-UC systems with reference numbers.

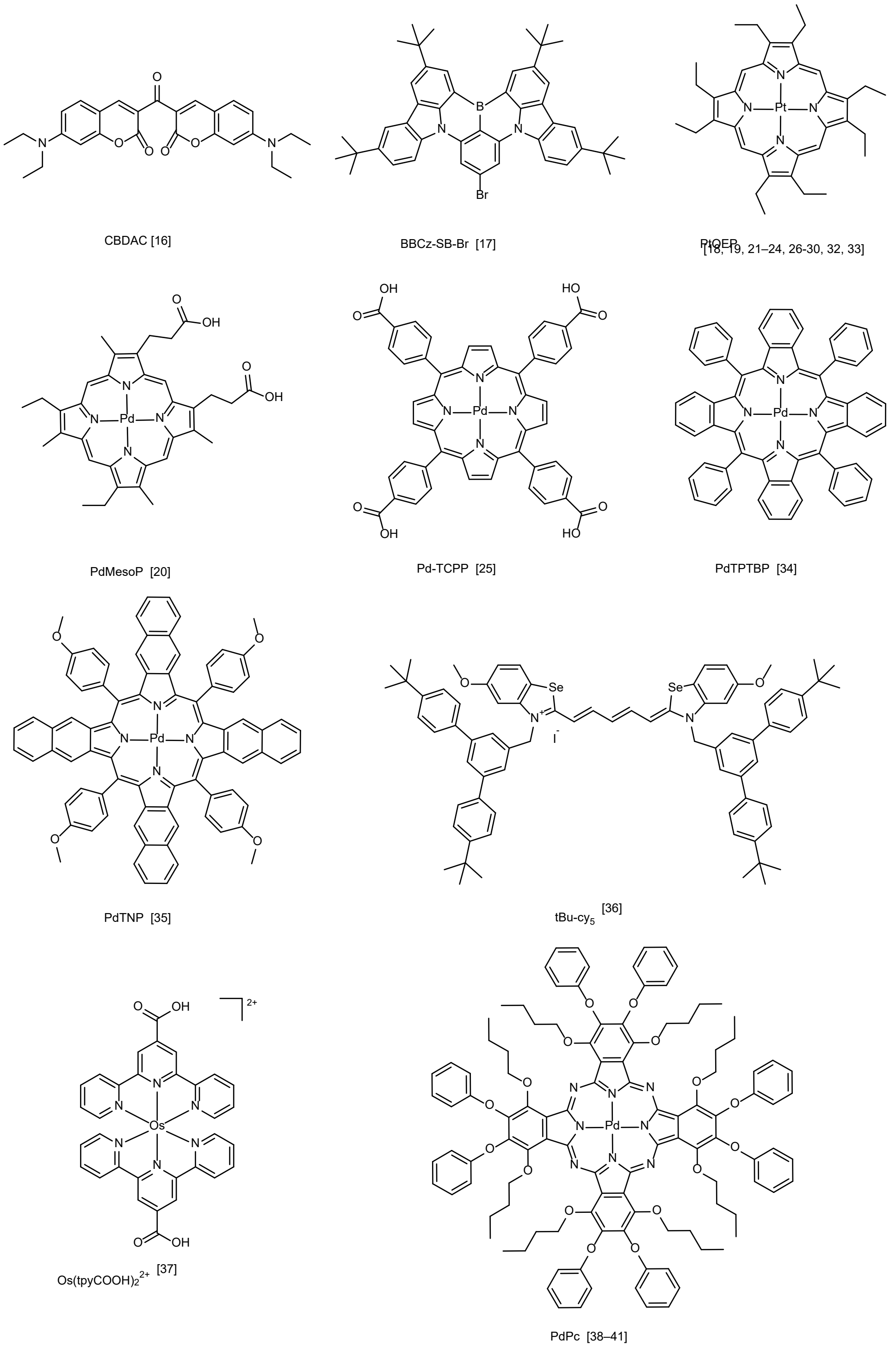

Supplementary Figure 33. Chemical structures of molecular triplet donors from Ref. 16 to 41.

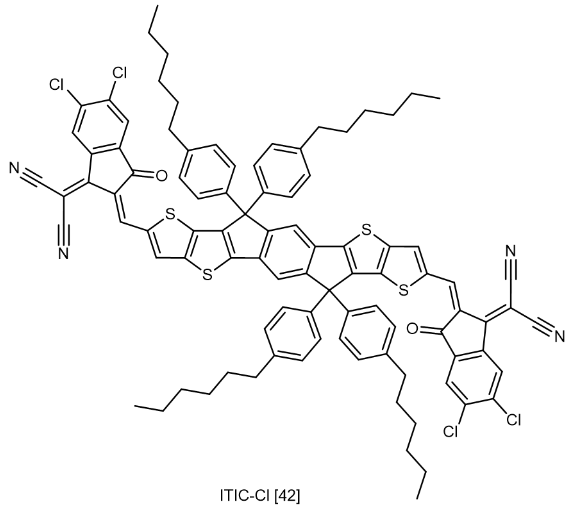

ITIC-Cl [42]

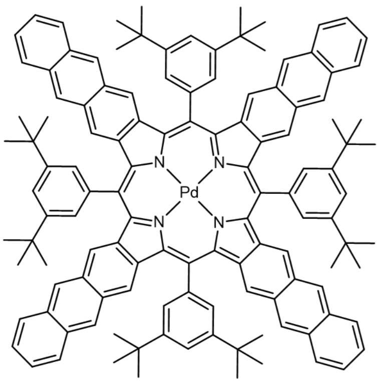

PdTPTAP [44]

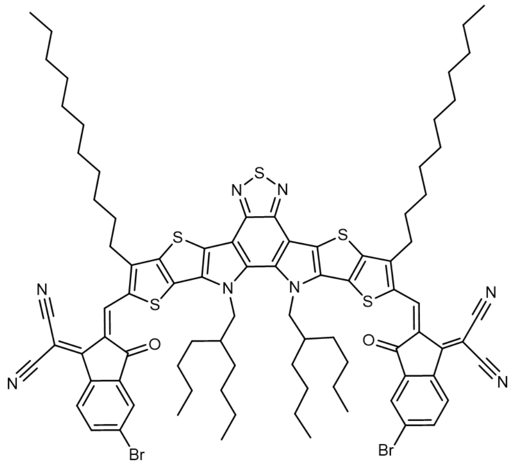

$PYIT_1$ [47]

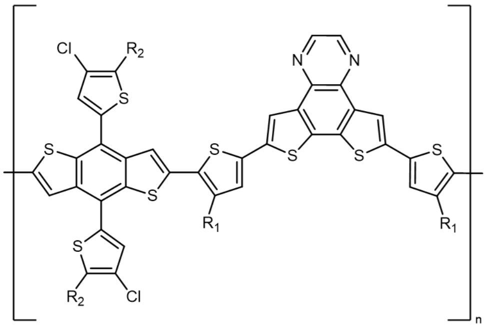

PBQx-TCl [47]

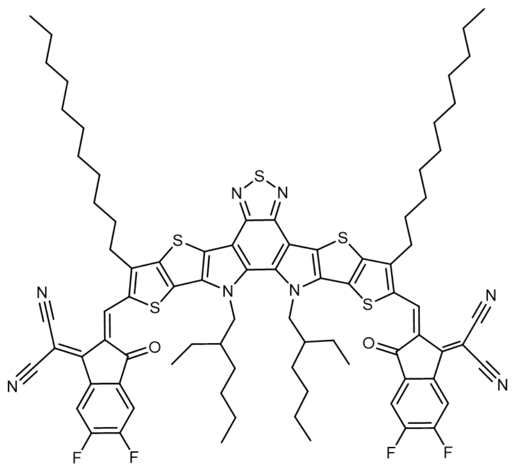

Y_6[42, 48]

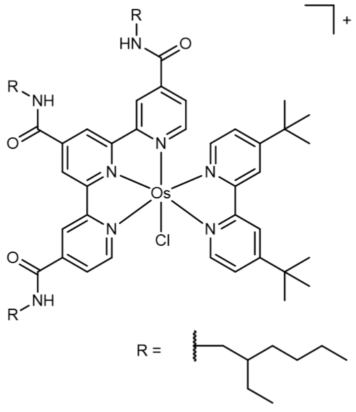

$Os(atpy)(tbbpy)Cl^+$ [50]

Supplementary Figure 34. Chemical structures of molecular triplet donors from Ref. 42 to 50.

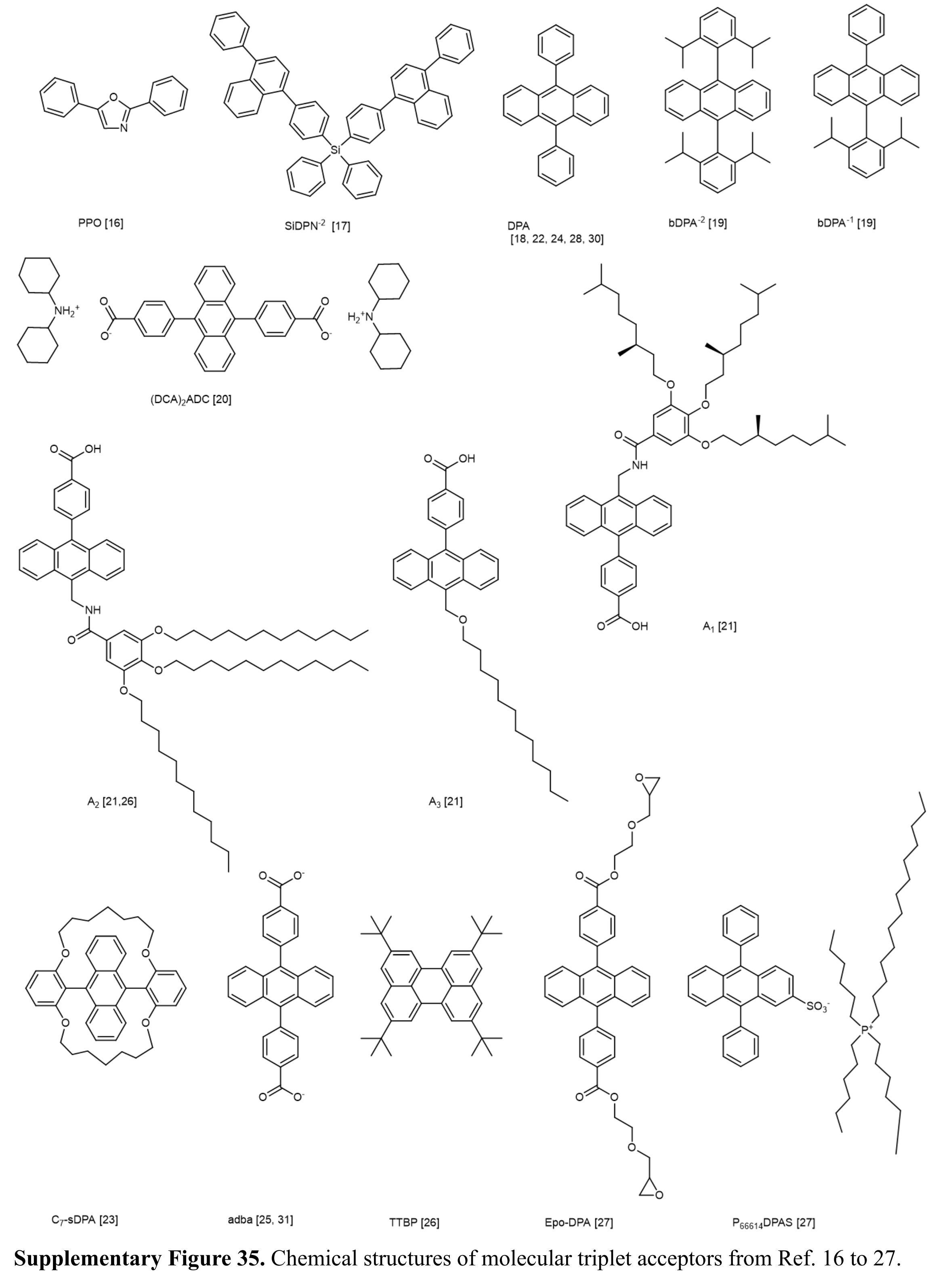

Supplementary Figure 35. Chemical structures of molecular triplet acceptors from Ref. 16 to 27.

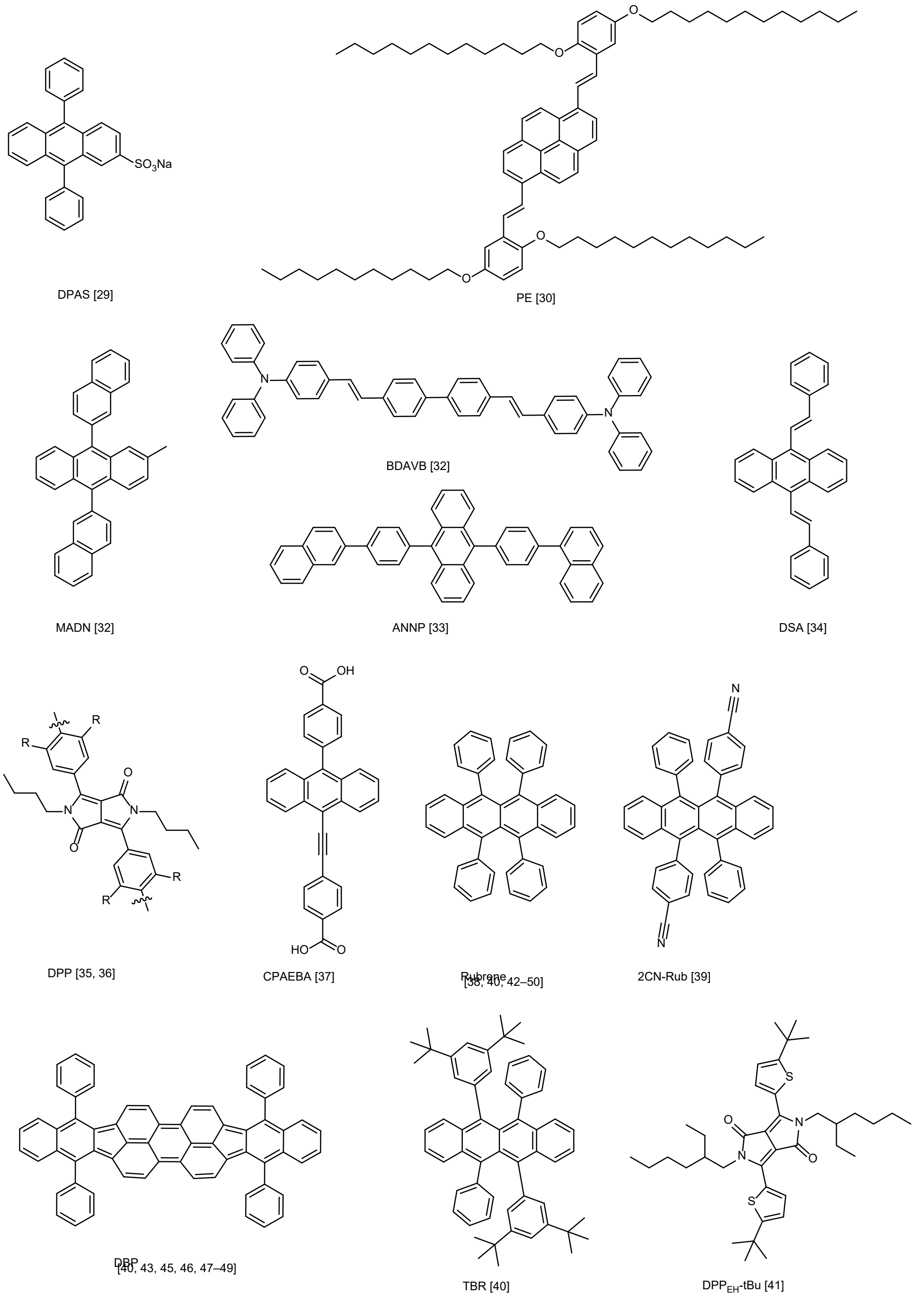

Supplementary Figure 36. Chemical structures of molecular triplet acceptors from Ref. 29 to 50.

Supplementary Table 8. Summary of energy migration based solid-state TTA-UC performances[a,b].

Excitation wavelength (nm)	UC emission wavelength (nm)	Donor[c]	Acceptor[c]	Φ_{UC}[d] (%)	I_{th} (mW cm^{-2})	Ref.
440	390–393	CBDAC	PPO	4.3	13.8	16
445	394	BBCz-SB-Br	SiDPN-2	1.3	44	17
530	435	PtOEP	DPA	3	5.6	18
532	427	PtOEP	bDPA-2	0.92	52	19
532	427	PtOEP	bDPA-1	0.31	110	19
532	435	PdMesoP	$(DCA)_2ADC$	3	49	20
532	436	PtOEP	DPA	0.022	200	19
532	440	PtOEP	A1	0.14	2.5	21
532	440	PtOEP	A2	0.1	1.7	21
532	440	PtOEP	DPA	2.5	6	22
532	440	PtOEP	C7-sDPA	5–10	5	23
532	440	PtOEP	DPA	0.022	450	24
532	440	PtOEP	DPA	1.0	531	24
532	440	PtOEP	DPA	0.22	326	24
532	440	Pd-TCPP	adba	0.64	2.5	25
532	440	PtOEP	A2/TTBP	4.5	195	26
532	440	PtOEP	Epo-DPA/ P_{66614}DPAS	5.7	39.1	27
532	440	PtOEP	DPA	8	5	28
532	445	PtOEP	DPAS	23.8	31.7	29
532	445	PtOEP	DPA/PE	2.7	39	30
532	450	CdSe/CdS	adba	0.00045	1000	31
532	470	PtOEP	A3	0.008	10.5	21
532	400–500	PtOEP	MADN/ BDAVBi	1.23	238	32
542	434	PtOEP	ANNP	13.4	2.1	33
640	514	PdTPTBP	DSA	0.29	35.9	34
721	573	PdTNP	DPP-PAF-2	1.5 (corrected)	1100	35
721	573	tBu-cy5	DPP-COF-2	7.6 (corrected)	980	36
724	540	$Os(tpyCOOH)_2^{2+}$	CPAEBA	0.006 (corrected)	10000	37
730	560	PdPc	Rubrene	1.2	1400	38
730	580	PdPc	2CN-Rub	3.0	3300	39

730	610	PdPc	Rubrene/DBP	0.07	2100	40
730	610	PdPc	TBR/DBP	0.3	2400	40
730	635	PdPc	DPP_{EH}-*t*Bu	1.06	16200	41
750	565	ITIC-Cl	Rubrene	1.42	39	42
772	610	$MoSe_2$	Rubrene/DBP	1.1	260	43
785	570	PdTPTAP	Rubrene	0.25	115	44
785	610	$MA_{0.15}FA_{0.85}PbI_3$	Rubrene/DBP	1.55	500	45
785	610	$MA_{0.15}FA_{0.85}PbI_3$	Rubrene/DBP	0.2445	61	46
808	573	PYIT1/PBQx-TCl	Rubrene	0.85	8	47
808	608	PYIT1/PBQx-TCl	Rubrene/DBP	2.2	10	47
808	610	Y6	Rubrene/DBP	0.0268	68.2	48
808	610	Y6	Rubrene/DBP	0.0179	92.5	48
808	612	PbS	Rubrene/DBP	0.26	17	49
808	612	PbS	Rubrene/DBP	0.62	12	49
808	612	PbS	Rubrene/DBP	0.11	26	49
850	565	Y6	Rubrene	0.515	19	42
938	580	Os(atpy)(tbbpy)Cl^+	Rubrene	1.55	10000	50

[a] Only reports documenting I_{th} and Φ_{UC} values are included in the summary.

[b] Some values were estimated from figures in the literature.

[c] Chemical structures are shown in Figure S33–36.

[d] The maximum value of Φ_{UC} is 50%.

Supplementary References

1. Song, S. *et al.* Stabilized polymers with novel indenoindene backbone against photodegradation for LEDs and solar cells. *Macromolecules* **41**, 7296–7305 (2008).

2. Frisch, M. J. *et al. Gaussian 16, Revision B.01*. (Gaussian, Inc., 2016).

3. Frisch, M. J. *et al. Gaussian 16, Revision C.01*. (Gaussian, Inc., 2019).

4. Dennington, R., Keith, T. A. & Millam, J. M. *GaussView, Version 6.1* (Shawnee Mission, KS: Semichem Inc., 2016).

5. Pope, M. & Swenberg, C. E. Single positive or negative carriers in organic crystals. in *Electronic Processes in Organic Crystals and Polymers* 192–455 (Oxford University Press, 1999).

6. Shim, S. C. & Chae, J. S. Photodimerization of Indeno[2,1-a]indene. *Bull. Chem. Soc. Jpn.* **55**, 1310–1312 (1982).

7. Yanai, N. *et al.* Absolute method to certify quantum yields of photon upconversion via triplet–triplet annihilation. *J. Phys. Chem. A* **123**, 10197–10203 (2019).

8. Detert, H. & Schollmeyer, D. CCDC 1949606: Experimental crystal structure determination. Cambridge Crystallographic Data Centre https://doi.org/10.5517/CCDC.CSD.CC23FQJT (2019).

9. Smith, M. B. & Michl, J. Singlet Fission. *Chem. Rev.* **110**, 6891–6936 (2010).

10. Berkelbach, T. C., Hybertsen, M. S. & Reichman, D. R. Microscopic theory of singlet exciton fission. II. Application to pentacene dimers and the role of superexchange. *J. Chem. Phys.* **138**,

114103 (2013).

11. Sato, R. *et al.* Synergetic effects of triplet–triplet annihilation and directional triplet exciton migration in organic crystals for photon upconversion. *J. Phys. Chem. Lett.* **9**, 6638–6643 (2018).

12. You, Z.-Q., Hsu, C.-P. & Fleming, G. R. Triplet–triplet energy-transfer coupling: theory and calculation. *J. Chem. Phys.* **124**, 044506 (2006).

13. Becke, A. D. Density-functional thermochemistry. III. The role of exact exchange. *J. Chem. Phys.* **98**, 5648–5652 (1993).

14. Krishnan, R., Binkley, J. S., Seeger, R. & Pople, J. A. Self-consistent molecular orbital methods. XX. A basis set for correlated wave functions. *J. Chem. Phys.* **72**, 650–654 (1980).

15. Frisch, M. J. *et al. Gaussian 16 Revision A.03*. (Gaussian, Inc., 2016).

16. Enomoto, R. & Murakami, Y. Solvent-free temperature gradient melt formation of efficient visible-to-UV photon upconversion organic films with subsolar threshold and over 100 h photostability in air. *J. Mater. Chem. C* **11**, 1678–1683 (2023).

17. Watanabe, S., Mizukami, K., Kimizuka, N. & Yasuda, T. Visible-to-UV photon upconversion in metal-free molecular aggregates based on glassy diphenylnaphthalene derivatives. *J. Mater. Chem. C* **12**, 10874–10878 (2024).

18. Monguzzi, A., Frigoli, M., Larpent, C., Tubino, R. & Meinardi, F. Low-power-photon up-conversion in dual-dye-loaded polymer nanoparticles. *Adv. Funct. Mater.* **22**, 139–143 (2012).

19. Gao, C. *et al.* Triplet fusion upconversion using sterically protected 9,10-diphenylanthracene as the emitter. *Phys. Chem. Chem. Phys.* **22**, 6300–6307 (2020).

20. Ogawa, T., Yanai, N., Fujiwara, S., Nguyen, T.-Q. & Kimizuka, N. Aggregation-free sensitizer dispersion in rigid ionic crystals for efficient solid-state photon upconversion and demonstration of defect effects. *J. Mater. Chem. C* **6**, 5609–5615 (2018).

21. Hosoyamada, M., Yanai, N., Ogawa, T. & Kimizuka, N. Molecularly dispersed donors in acceptor molecular crystals for photon upconversion under low excitation intensity. *Chem. Eur. J.* **22**, 2060–2067 (2016).

22. Monguzzi, A. *et al.* Unraveling triplet excitons photophysics in hyper-cross-linked polymeric nanoparticles: toward the next generation of solid-state upconverting materials. *J. Phys. Chem. Lett.* **7**, 2779–2785 (2016).

23. Kamada, K. *et al.* Efficient triplet–triplet annihilation upconversion in binary crystalline solids fabricated via solution casting and operated in air. *Mater. Horiz.* **4**, 83–87 (2017).

24. Ogawa, T., Yanai, N., Kouno, H. & Kimizuka, N. Kinetically controlled crystal growth approach to enhance triplet energy migration-based photon upconversion. *J. Photonics Energy* **8**, 022003 (2017).

25. Park, J., Xu, M., Li, F. & Zhou, H.-C. 3D long-range triplet migration in a water-stable metal–organic framework for upconversion-based ultralow-power in vivo imaging. *J. Am. Chem. Soc.*

140, 5493–5499 (2018).

26. Ogawa, T. *et al.* Donor–acceptor–collector ternary crystalline films for efficient solid-state photon upconversion. *J. Am. Chem. Soc.* **140**, 8788–8796 (2018).
27. Kashino, T. *et al.* Design guidelines for rigid epoxy resins with high photon upconversion efficiency: critical role of emitter concentration. *ACS Appl. Mater. Interfaces* **14**, 22771–22780 (2022).
28. Raišys, S., Juršėnas, S. & Kazlauskas, K. Boost in solid-state photon upconversion efficiency through combined approach of melt-processing and purification. *Sol. RRL* **6**, 2100873 (2022).
29. Wei, L. *et al.* Triplet-triplet annihilation upconversion in LAPONITE®/PVP nanocomposites: absolute quantum yields of up to 23.8% in the solid state and application to anti-counterfeiting. *Mater. Horiz.* **9**, 3048–3056 (2022).
30. Raišys, S., Juršėnas, S., Simon, Y. C., Weder, C. & Kazlauskas, K. Enhancement of triplet-sensitized upconversion in rigid polymers via singlet exciton sink approach. *Chem. Sci.* **9**, 6796–6802 (2018).
31. Amemori, S. *et al.* Hybridizing semiconductor nanocrystals with metal–organic frameworks for visible and near-infrared photon upconversion. *Dalton Trans.* **47**, 8590–8594 (2018).
32. Lin, T.-A., Perkinson, C. F. & Baldo, M. A. Strategies for high-performance solid-state triplet-triplet-annihilation-based photon upconversion. *Adv. Mater.* **32**, e1908175 (2020).

33. Enomoto, R. *et al.* van der Waals solid solution crystals for highly efficient in-air photon upconversion under subsolar irradiance. *Mater. Horiz.* **8**, 3449–3456 (2021).

34. Li, L. *et al.* Light-harvesting organic nanocrystals capable of photon upconversion. *ChemSusChem* **10**, 4610–4615 (2017).

35. Zhang, M.-Y. *et al.* High-performance 721 nm-excitable photon upconversion porous aromatic frameworks for broad-range oxygen sensing and efficient heterogeneous photoredox catalysis. *Adv. Mater.* **37**, e2502150 (2025).

36. Zhang, M.-Y. *et al.* Triplet–triplet annihilation upconversion in covalent organic frameworks via interface and bulk exciton tunability. *Chem* 102886 (2026).

37. Joarder, B. *et al.* Near-infrared-to-visible photon upconversion by introducing an S−T absorption sensitizer into a metal-organic framework. *ChemNanoMat* **6**, 916–919 (2020).

38. Radiunas, E. *et al.* Efficient NIR-to-vis photon upconversion in binary rubrene films deposited by simplified thermal evaporation. *J. Mater. Chem. C* **10**, 6314–6322 (2022).

39. Radiunas, E. *et al.* CN-Tuning: a pathway to suppress singlet fission and amplify triplet-triplet annihilation upconversion in rubrene. *Adv. Opt. Mater.* **13**, 2403032 (2025).

40. Radiunas, E. *et al.* Impact of *t*-butyl substitution in a rubrene emitter for solid state NIR-to-visible photon upconversion. *Phys. Chem. Chem. Phys.* **22**, 7392–7403 (2020).

41. Naimovicius, L. *et al.* Activating solid-state triplet–triplet annihilation upconversion via bulky

annihilators. *J. Am. Chem. Soc.* **148**, 3811–3819 (2026).

42. Izawa, S. & Hiramoto, M. Efficient solid-state photon upconversion enabled by triplet formation at an organic semiconductor interface. *Nat. Photonics* **15**, 895–900 (2021).
43. Duan, J. *et al.* Efficient solid-state infrared-to-visible photon upconversion on atomically thin monolayer semiconductors. *Sci. Adv.* **8**, eabq4935 (2022).
44. Abulikemu, A. *et al.* Solid-state, near-infrared to visible photon upconversion via triplet-triplet annihilation of a binary system fabricated by solution casting. *ACS Appl. Mater. Interfaces* **11**, 20812–20819 (2019).
45. Nienhaus, L. *et al.* Triplet-sensitization by lead halide perovskite thin films for near-infrared-to-visible upconversion. *ACS Energy Lett.* **4**, 888–895 (2019).
46. Wang, L. *et al.* Interfacial trap-assisted triplet generation in lead halide perovskite sensitized solid-state upconversion. *Adv. Mater.* **33**, e2100854 (2021).
47. Bi, P. *et al.* Donor-acceptor bulk-heterojunction sensitizer for efficient solid-state infrared-to-visible photon up-conversion. *Nat. Commun.* **15**, 5719 (2024).
48. Hu, M. *et al.* Bulk heterojunction upconversion thin films fabricated via one-step solution deposition. *ACS Nano* **17**, 22642–22655 (2023).
49. Wu, M. *et al.* Solid-state infrared-to-visible upconversion sensitized by colloidal nanocrystals. *Nat. Photonics* **10**, 31–34 (2016).

50. Amemori, S., Sasaki, Y., Yanai, N. & Kimizuka, N. Near-infrared-to-visible photon upconversion sensitized by a metal complex with spin-forbidden yet strong S_0–T_1 absorption. *J. Am. Chem. Soc.* **138**, 8702–8705 (2016).
