# Supplementary material for: Sterically protected π-electron systems for efficient solid-state photon upconversion
Source: Nat Commun. 2026 Jun 23;17:5134. doi: 10.1038/s41467-026-73898-0 (PMC13291331; doi:10.1038/s41467-026-73898-0)
Supplement: Supplementary file 2 — Transparent Peer Review file [file 41467_2026_73898_MOESM2_ESM.pdf]

nature portfolio

Peer Review File

Sterically Protected π-Electron Systems for Efficient Solid-State Photon Upconversion

Corresponding Author: Professor Yoichi Sasaki

This file contains all reviewer reports in order by version, followed by all author rebuttals in order by version.

Version 0:

Reviewer comments:

Reviewer #1

(Remarks to the Author)
Kimizuka, Sasaki and group members present a novel annihilator design for blue-to-UV upconversion systems. As a result of the smart choice of the basic annihilator structure, alkyl chains could be introduced at an sp^3 carbon atom resulting in sufficient steric hindrance while allowing high fluorescence quantum yield and suppressing quenching.
In a crystal co-doped with an iridium-based sensitizer, an outstanding upconversion performance regarding both the quantum yield and the threshold intensity was obtained, which is mainly due to the molecular design enabling energy diffusion and an essentially homogeneous distribution of the sensitizer molecules. With 4CzIPN and DABNA as sensitizers, purely organic systems could be realized.
The quality of the whole study, including all the experiments and the way the results are presented, is extremely high.
I already know the underlying preprint and I am deeply impressed by the ground-breaking results.
Therefore, I am happy to recommend this paper for Nat. Commun. after minor revisions as described below.

Minor points and questions:

-I agree that the presented system would likely operate under sunlight conditions, but the authors did not show this experiment in their paper, which is why I suggest to remove sunlight from the title.

-introduction: Albinsson et al. reported a quantum yield as high as 16.4 %, corresponding to an efficiency of 32.8 %, in solution (see J. Am. Chem. Soc. 2023, 145, 40, 22168–22175). Hence, the 27.6 % in the introduction have to be updated.

-SI, DFT part: I thought that the same basis set should be used for both the ground state optimization and the subsequent prediction of properties to get reliable results. Did the authors really change the settings after the structure optimization or is it maybe written in a confusing way?

- Fig. S7.: Spectra are normalized. Nevertheless it would be better to show 0 and 1 at the y axis.

- A curiosity-based question: Could the authors comment on the thickness of the spin-coated films for vis-to-UV upconversion?

- An additional table in the SI with the UC efficiencies and I_th values from Fig. 3e (and the corresponding references) would be extremely helpful. Moreover, are corrected (for self absorption) or uncorrected literature values included. This is crucial for the comparison to be fair.

Reviewer #2

(Remarks to the Author)
The study by N. Harada et al. developed a solid-state visible-to-UV triplet–triplet annihilation-based photon upconversion (TTA-UC) system, achieving a high upconversion efficiency of 3.8% in the solid state with a low excitation threshold intensity of 1.2 mW cm−2. By functionalizing dihydro-indenoindene derivatives with alkyl chains, the researchers optimized interchromophore interactions, suppressed quenching of excited states, and achieved high fluorescence quantum yields of over 70% in both solution and crystalline states. The iBu-DHI acceptor demonstrated superior performance due to its controlled intermolecular distances and high triplet lifetime of 4.0 ms. This molecular design offers tolerance to crystalline defects and oxygen quenching, marking a significant advancement in solid-state TTA-UC systems.

However, the numerous flaws and data uncertainties outlined below raise serious concerns about the reliability of the reported results, making the manuscript unsuitable for publication in its current form. Furthermore, the study's narrow scope may not align with the broad interdisciplinary readership of Nature Communications and may be better suited to a more specialized journal.

1. The abstract claims "We found an optimal emitter structure exhibiting the highest UC efficiency in both solution and crystalline state." This should be redefined as optimal among the four DHI derivatives studied, since untested substituents like tert-butyl or other groups might achieve even better solid-state UC performance.
2. The use of "UC efficiency" in Eq. 3 (SI) and throughout the text, instead of "quantum yield (QY)" is inconsistent, as all right-side terms, including fluorescence, are QYs, confusing readers. This choice is made to double the conventional UC QY to inflate its value, despite the well-established 50% theoretical UC QY limit. Such multiplication by two is widely discouraged in the field and must be avoided. As a partial solution, UC efficiency could be indicated in brackets alongside UC QY values.
3. The significant reduction in PL QY of unsubstituted DHI in crystals compared to solution is attributed to energy traps promoting singlet exciton deactivation. However, molecular geometry changes upon crystallization could also enhance intersystem crossing to the triplet state, an alternative deactivation pathway not considered. The authors are requested to measure phosphorescence in crystals at low temperatures to confirm this channel.
4. The choice of Ir(ppy)3 as a donor is flawed due to strong overlap between acceptor emission and donor absorption, likely causing significant BET and emission quenching. The authors should measure PL QY of acceptors with and without the donor in solution and crystals to evaluate BET effects.
5. It is unclear why the PL lifetime remains unchanged despite the BET-induced quenching. The authors should clarify this inconsistency, as this quenching typically affects both PL QY and lifetime.
6. FL decays profiles were provided only for iBu-DHI spin-coated films, with no data for other derivatives.
7. The UC emission spectra in Fig. S9 are truncated at 350 nm, showing only partial spectra (e.g., only half in Fig. S9a), limiting analysis. The authors should measure spectra from at least 280 nm using quartz optics to capture the full UC emission range. This may have also led to unreliable UC QY estimation as detailed in Sect. 8 (SI), since only part of UC spectra was measured. Note also that the UC spectrum in Fig. S15a abruptly changes at 350nm.
8. The UC QY of unsubstituted DHI, which has the highest Ith, saturates at a lower excitation intensity (~2500 mW/cm²) compared to Me-DHI and iBu-DHI (>5000 mW/cm²), raising concerns about data consistency.
9. Fig. S15a labels the Y-axis as "normalized intensity," but the spectra are clearly not normalized.
10. Regarding the possible 2-EtBu-DHI aggregation, suggested by Fig. S9d data. The authors are requested to test lower acceptor concentrations, such as 1 mM (as used for DHI) or even less, and include UC data for 2-EtBu-DHI at these concentrations in Fig. S10a-c.
11. The UC QY of unsubstituted DHI, which has the highest threshold intensity (Ith), saturates at a lower excitation intensity (~2500 mW/cm²) compared to Me-DHI and iBu-DHI (>5000 mW/cm²), raising concerns about data consistency.
12. To substantiate their claim of aggregate- or dimer-related excited state quenching in DHI solutions, the authors should conduct acceptor concentration-dependent measurements of spectra, lifetime, and QY.
13. To enable a fair comparison of DHI derivatives in Table S2, all parameters must be measured at a consistent acceptor concentration (1 mM or lower) and in the absence of aggregation. 2-EtBu-DHI must be included in the analysis as well.
14. Fig. 3 lacks data for unsubstituted DHI crystalline films, which is essential for a comprehensive comparison.
15. The claim that "deactivation of excited states by exciton trap sites in crystalline DHI systems is avoided by de-trapping via thermal excitation" is highly questionable. If true, the PLQY should decrease at lower temperatures due to insufficient thermal energy for de-trapping. This requires verification.
16. Although the authors identify iBu-DHI as the best crystalline-state acceptor to date, they should also emphasize its practical limitations, namely, the difficulty in controlling crystal size, grain boundaries, and ensuring consistent UC and PL performance across batches.
17. It is unclear why the average phosphorescence lifetime of the $Ir(ppy)_3$ donor increases in the iBu-DHI film (Table S3), whereas in other DHI films it decreases significantly due to TET.
18. What are the UCQY and Ith at t=0 min for the spin-coated iBu-DHI/$Ir(ppy)_3$ film prepared under air-saturated conditions (Fig. S19)?
19. The authors claim that the $Ir(ppy)_3$ donor is homogeneously distributed within DHI microcrystals without segregation, based on SEM and SEM-EDX data. However, these techniques cannot distinguish whether the donor is embedded within the crystals or merely on their surface. Additional evidence is needed, or the statement should be rephrased accordingly.
20. The authors state that "the drastic reduction of the Ith value for iBu-DHI/Ir(ppy)3 implies fast triplet energy diffusion in the crystal", but this is not necessarily true. A lower Ith could also result from significantly enhanced TET from donor to acceptor. To distinguish between TET and diffusion contributions, the authors should quantify TET in spin-coated DHI films, for example, by conducting donor phosphorescence quenching experiments with increasing acceptor concentration in an inert polymer matrix.

Reviewer #3

(Remarks to the Author)
In this work, the authors report the fabrication of triplet-triplet annihilation upconversion solid-state devices. The authors use the typical Ir(ppy)3 as sensitizer and present a series of four annihilators: the indenoindene backbone and three derivatives obtained by introducing alkyl spacers at the two sp3 carbons. The authors characterize TTA-UC in solution and solid-state by means of optical measurements to find that in both cases, iBu-DHI is the upconversion efficiency champion, which the authors relate to its higher PLQY, longer triplet lifetime and faster triplet diffusion. Compositional homogeneity of the films is probed using SEM-EDX, which confirms no sensitizer segregation and X-ray diffraction is employed to evaluate crystallinity, with comparison to single crystal structures. Steady-state and time-resolved photophysical measurements are used to determine PLQY, threshold intensity, and excited-state lifetimes. A strong upconversion efficiency from visible light to UV is achieved under excitation intensities below one sun. Additionally, the authors find that the more compact crystalline structure of iBu-DHI prevents oxygen permeation and enables the measurement of UC emission up to 1 hour in an air environment. Overall, the work is reasonably well executed and important for the field, and publication could be considered after addressing the following points:

Major comments:
• The authors attribute the increased UC efficiency to higher PLQY, increased lifetime and fast triplet diffusion. Please make clearer evidence to support faster triplet diffusion.
• The authors claim that these devices are unaffected by the formation of crystalline domains. However, without a direct comparison to an amorphous analogue or analysis of morphology–performance correlations, it is difficult to substantiate this claim. Additional evidence is necessary.
• Page 7, Figure S9: the authors wrote “aggregation of 2-EtBu-DHI was observed upon 445nm laser excitation, which might be the reason for negligible UC emission”. Could the authors comment on why the compound with the longer alkyl chains is the one susceptible to aggregation? Was the compound stable in solution upon excitation for other measurements (e.g. quantum yield, fluorescence lifetime)? The compound can be dissolved in other solvents (i.e. methylene chloride is used for characterization by NMR spectroscopy); does this aggregation occur also in other solvents? Can the authors comment on the possibility of this aggregation being the reason why solid-state devices with 2-EtBu-DHI as an acceptor have the lowest performance?
• In page 9, the authors comment on the similarities/differences in XRD diffraction patterns between single crystals and deposited samples. Given the difference in the position of the main reflection between drop casted and spin coated samples, could the authors comment on what does this imply (i.e. preferred crystalline orientation) and the effect it might have on TTA-UC?
• Page S23, Figure S19. In panel a, add legend/arrow/color code indicating the changes in spectra upon time.
The caption of figure S19a states that the UC emission of the spin-coated film was “prepared and measured in air”; contrarily, in the main script (page 11) the text states “prepared in air-saturated conditions showed UC emission without encapsulation in an inert atmosphere”. These statements seem contradictory. Could the authors clarify?
In some solid-state devices, it has been observed that higher power density excitation can lead to sample degradation, even under inert conditions. Could the authors comment on why they chose to use different power densities (up to 3 orders of magnitude). This experiment should be repeated comparing both samples at the same power density excitation (presumably the highest one, which is needed to activate TTA-UC in the presence of oxygen).
The authors state that the high-density molecular assembly of iBu-DHI/Ir(ppy)3 prevents oxygen diffusion. Would the 2-EtBu-DHI films be considered lower-density packaging, given that the distance between DHI units is larger for this compound? If so, could they compare the stability against oxygen for these two films?
In my opinion, Figure 4b is not sufficient to make claims about Irppy dispersion, as the scale is dramatically too big to draw conclusions.

Minor comments:
• Please avoid claims like “never been developed”
• The field has moved away from the “free factor of 2” in upconversion efficiencies. Please consider removing from your calculations.
• In figure 1b, the authors should describe the chemical nature of the sensitizer.
• In figure 2, the ground and solution fluorescent lifetimes are hard to distinguish, having the same marker and a very similar color. Authors should change this for greater clarity. Arrows to indicate which axis is related to markers and bars should be added.
• Page 10, Figure 3c: missing a legend (green, blue and red points can be deduced from Figure 3b, not grey data points, which is the same acceptor as red but drop-casted instead of spinned). Additionally, authors should add Ith to all of them.
• Page S14, Figure S11. Legend indicates laser used is 369 nm while caption states 445 nm.
• The addition of the crystalline structure of DHI would help visualize the pi-stacking that is prevented in the derivatives thanks to the addition of the alkyl chains.

Version 1:

Reviewer comments:

Reviewer #1

(Remarks to the Author)
The authors addressed all of the (mostly minor) issues that I had very carefully.
I also had a look at the comments of the other referees and I believe that the authors did a great job during the revision. The manuscript with the outstanding results on solid-state upconversion systems should be published without further delay as the findings and the high-level methodology will certainly stimulate further research activities.

Reviewer #2

(Remarks to the Author)
While the revision addresses some points, several issues still remain and require further revision.

1) The authors introduced a ½ prefactor in Eq. 3 (SI), enforcing the 50% maximum for UC QY, but the revised manuscript’s relative-method equation for UC QY omits this prefactor and therefore treats UC QY like an ordinary PLQY. As written, that equation can output up to 100% (since PLQY is bounded by 100%), which is inconsistent with their SI definition and leaves the “factor of 2” problem unresolved.
2) The claim “no new 77K emission features ⇒ no ISC-mediated deactivation” (Fig. R4) is not justified, because phosphorescence can be fully quenched nonradiatively or too weak to be observed under these measurement conditions. So, these results cannot exclude ISC as a competing deactivation channel.
3) Fig. 3e and Table S8 do not provide a clean “solid-state” benchmark: it mixes genuinely rigid systems with low-T_g matrices (e.g., Ref. 16, 31) where molecular diffusion contributes to TTA-UC, making the comparison misleading. At the same time, authors omit relevant high-performing, truly solid-state reports (e.g., Mater. Horiz., 2022,9, 3048; Sol. RRL 2022, 2100873, Adv. Optical Mater. 2025, 13, 2403032), so the literature context is selective and incomplete.

Reviewer #3

(Remarks to the Author)
I thank the authors for their work and changes to the manuscript. I feel my concerns have been well addressed, though I strong encourage the authors to include the information (particularly the aggregation work/R20) in the manuscript or supporting information, as it could be quite helpful for follow-on studies.

I highlight that I found the other reviewers' comments to be quite reasonable, and therefore they should be fully resolved before publication.

Open Access This Peer Review File is licensed under a Creative Commons Attribution 4.0 International License, which permits use, sharing, adaptation, distribution and reproduction in any medium or format, as long as you give appropriate credit to the original author(s) and the source, provide a link to the Creative Commons license, and indicate if changes were made.
In cases where reviewers are anonymous, credit should be given to 'Anonymous Referee' and the source.
The images or other third party material in this Peer Review File are included in the article’s Creative Commons license, unless indicated otherwise in a credit line to the material. If material is not included in the article’s Creative Commons license and your intended use is not permitted by statutory regulation or exceeds the permitted use, you will need to obtain

permission directly from the copyright holder.
To view a copy of this license, visit https://creativecommons.org/licenses/by/4.0/

Response Letter

Reviewer 1's comments

Kimizuka, Sasaki and group members present a novel annihilator design for blue-to-UV upconversion systems. As a result of the smart choice of the basic annihilator structure, alkyl chains could be introduced at an sp^3 carbon atom resulting in sufficient steric hindrance while allowing high fluorescence quantum yield and suppressing quenching. In a crystal co-doped with an iridium-based sensitizer, an outstanding upconversion performance regarding both the quantum yield and the threshold intensity was obtained, which is mainly due to the molecular design enabling energy diffusion and an essentially homogeneous distribution of the sensitizer molecules. With 4CzIPN and DABNA as sensitizers, purely organic systems could be realized. The quality of the whole study, including all the experiments and the way the results are presented, is extremely high. I already know the underlying preprint and I am deeply impressed by the ground-breaking results. Therefore, I am happy to recommend this paper for Nat. Commun. after minor revisions as described below.

We greatly appreciate the reviewer's positive comments.

Minor points and questions:

-I agree that the presented system would likely operate under sunlight conditions, but the authors did not show this experiment in their paper, which is why I suggest to remove sunlight from the title.

We appreciate this suggestion. Following the reviewer's suggestion, we have removed the word "Sunlight-Driven" from the title and updated it to "Sterically Protected π-Electron Systems: A Viable Solution for Efficient Solid-State Photon Upconversion".

-introduction: Albinsson et al. reported a quantum yield as high as 16.4 %, corresponding to an efficiency of 32.8 %, in solution (see J. Am. Chem. Soc. 2023, 145, 40, 22168–22175). Hence, the 27.6 % in the introduction have to be updated.

Following the reviewer's comment, the quantum yield has been updated to 16.4% (page 3, line 1).

-SI, DFT part: I thought that the same basis set should be used for both the ground state optimization and the subsequent prediction of properties to get reliable results. Did the authors really change the settings after the structure optimization or is it maybe written in a confusing way?

We appreciate the comment. We have replaced the figure with the results obtained with the same basis set of 6-311++G(d,p) (Figure R1, S5).

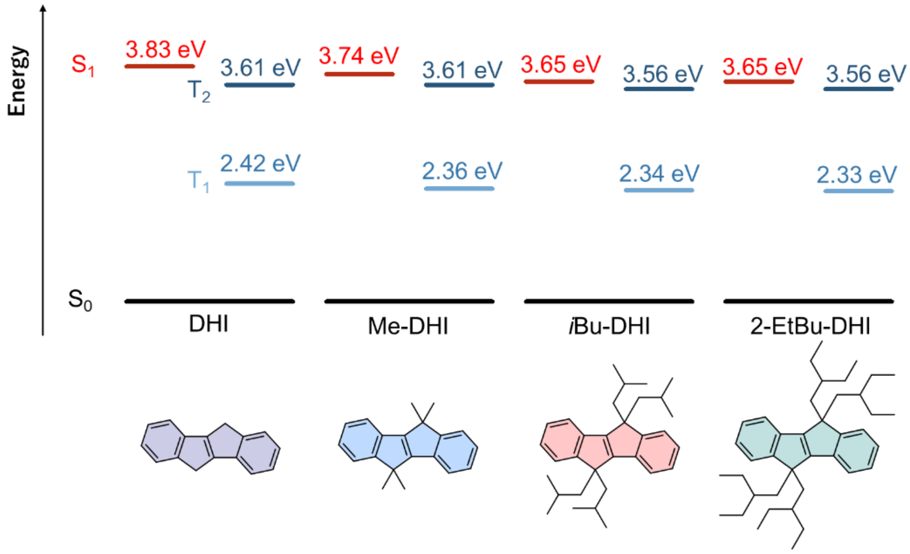

Figure R1. Calculated singlet and triplet energy levels of DHI, Me-DHI, *i*Bu-DHI, and 2-EtBu-DHI by DFT calculations (TD-B3LYP/6-311++G(d,p) // B3LYP/6-311++G(d,p)).

- Fig. S7.: Spectra are normalized. Nevertheless it would be better to show 0 and 1 at the y axis.

We appreciate this suggestion. We have modified the graph in the revised manuscript as follows (Figure R2, S7).

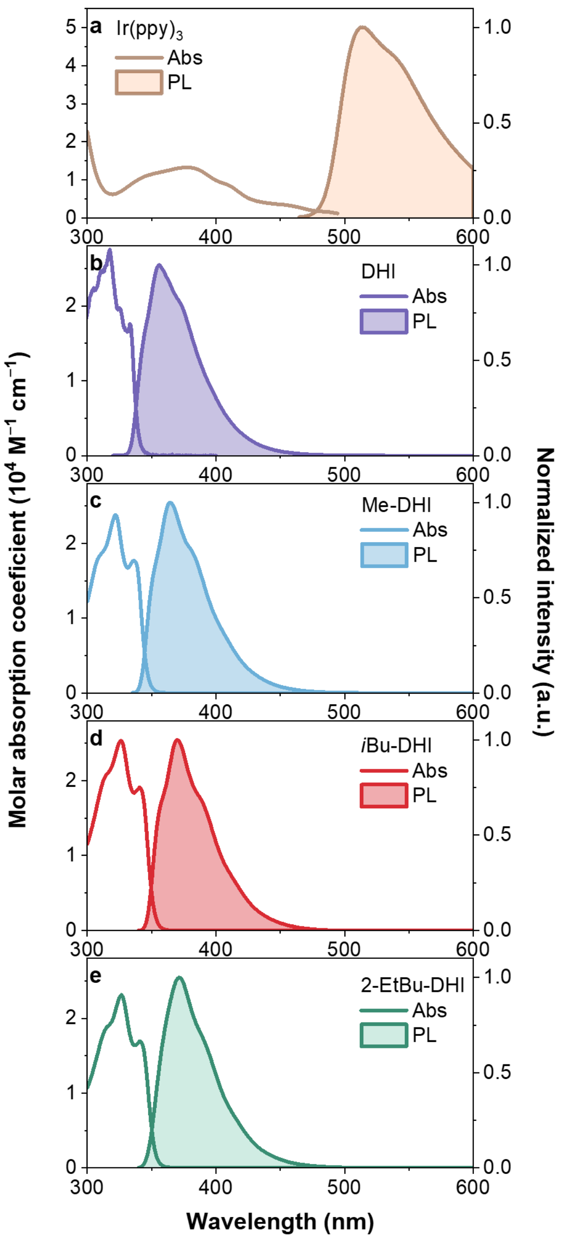

Figure R2. Absorption spectra (molar absorption coefficient) (solid line) and normalized emission (filled in) spectra of **a** $Ir(ppy)_3$, **b** DHI, **c** Me-DHI, **d** *i*Bu-DHI, and **e** 2-EtBu-DHI in THF ($[Ir(ppy)_3]$ = 100 μM, [DHI and DHI derivatives] = 200 μM).

- A curiosity-based question: Could the authors comment on the thickness of the spin-coated films for vis-to-UV upconversion?

We appreciate this comment. We have conducted step-height analysis of the spin-coated films of Me-DHI, *i*Bu-DHI, and 2-EtBu-DHI with $Ir(ppy)_3$. The average thicknesses were 82.2 nm, 128 nm, and 297 nm, respectively. We inserted the following comments in the sample preparation section in the revised manuscript (page 18, line 6).

"Step-height analysis with a Surfcorder ET150 (Kosaka Laboratory Ltd.) revealed the following average thicknesses for the $Ir(ppy)_3$ doped spin-coated films of Me-DHI, *i*Bu-DHI and 2-EtBu-DHI: 82.2 nm, 128 nm, and 297 nm, respectively."

- An additional table in the SI with the UC efficiencies and Ith values from Fig. 3e (and the corresponding references) would be extremely helpful. Moreover, are corrected (for self absorption) or uncorrected literature values included. This is crucial for the comparison to be fair.

We appreciate this positive comment. Following the reviewer's suggestion, we have updated the literature values in Table R1, including whether they were corrected for self-absorption (Figure S30, R3, Table R1, S8).

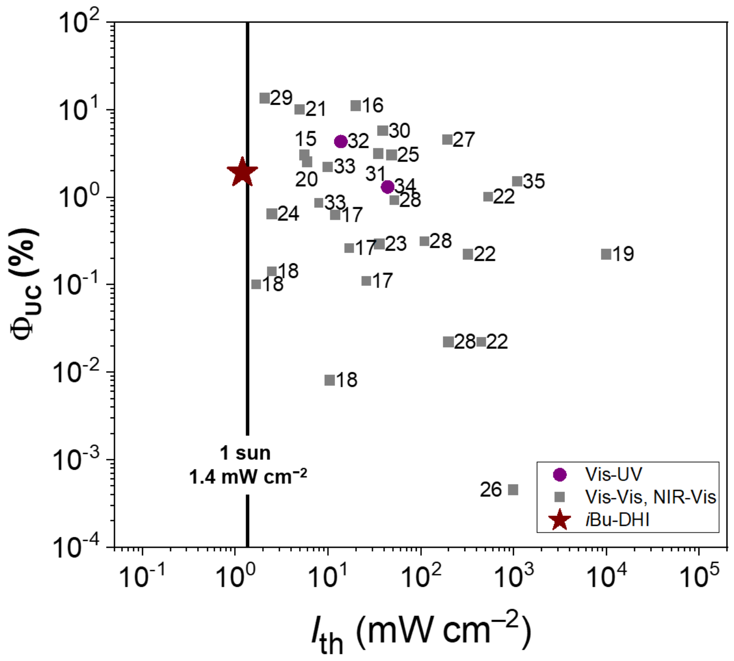

Figure R3. Φ_{UC}–I_{th} plots with the reported values of solid TTA-UC systems with reference numbers.

Table R1. Summary of solid-state TTA-UC performances reported to date. Note that the maximum value of Φ_{UC} is 50%.

Excitation wavelength (nm)	UC emission wavelength (nm)	Φ_{UC} (%)	I_{th} (mW cm^{-2})	Reference
530	435	3	5.6	15
514.5	430	11	20	16
808	612	0.62	12	17
808	612	0.26	17	17
808	612	0.11	26	17
532	470	0.008	10.5	18
532	440	0.1	1.7	18
532	440	0.14	2.5	18
938	580	0.22	10000	19
532	440	2.5	6	20
532	440	5−10	5	21
532	440	0.22	326	22
532	440	1.0	531	22
532	440	0.022	450	22
640	514	0.29	35.9	23
532	440	0.64	2.5	24
532	435	3	49	25
785	450	0.00045	1000	26
532	440	4.5	195	27
532	436	0.022	200	28
532	427	0.31	110	28
532	427	0.92	52	28
542	434	13.4	2.1	29
532	440	5.7	39.1	30
635	470	3.1	35	31
440	390–393	4.3	13.8	32
808	608	2.2	10	33
808	608	0.85	8	33
445	394	1.3	44	34
721	573	1.5 (corrected)	1100	35

Reviewer 2's comments

The study by N. Harada et al. developed a solid-state visible-to-UV triplet–triplet annihilation-based photon upconversion (TTA-UC) system, achieving a high upconversion efficiency of 3.8% in the solid state with a low excitation threshold intensity of 1.2 mW cm−2. By functionalizing dihydro-indenoindene derivatives with alkyl chains, the researchers optimized interchromophore interactions, suppressed quenching of excited states, and achieved high fluorescence quantum yields of over 70% in both solution and crystalline states. The iBu-DHI acceptor demonstrated superior performance due to its controlled intermolecular distances and high triplet lifetime of 4.0 ms. This molecular design offers tolerance to crystalline defects and oxygen quenching, marking a significant advancement in solid-state TTA-UC systems.

However, the numerous flaws and data uncertainties outlined below raise serious concerns about the reliability of the reported results, making the manuscript unsuitable for publication in its current form. Furthermore, the study's narrow scope may not align with the broad interdisciplinary readership of Nature Communications and may be better suited to a more specialized journal.

We appreciate the reviewer for carefully reading the manuscript and providing detailed feedback.

1. The abstract claims "We found an optimal emitter structure exhibiting the highest UC efficiency in both solution and crystalline state." This should be redefined as optimal among the four DHI derivatives studied, since untested substituents like tert-butyl or other groups might achieve even better solid-state UC performance.

We appreciate this comment. Following the reviewer's suggestion, we have revised the sentence as follows (page 2, line 1).

"Among four DHI derivatives investigated, we identified the optimal emitter structure that exhibits the highest UC quantum yield in both solution and crystalline states."

2. The use of "UC efficiency" in Eq. 3 (SI) and throughout the text, instead of "quantum yield (QY)" is inconsistent, as all right-side terms, including fluorescence, are QYs, confusing readers. This choice is made to double the conventional UC QY to inflate its value, despite the well-established 50% theoretical UC QY limit. Such multiplication by two is widely discouraged in the field and must be avoided. As a partial solution, UC efficiency could be indicated in brackets alongside UC QY values.

We appreciate the comment. The UC efficiencies were replaced with Φ_{UC} in the revised manuscript as follows (Table S8, R1).

Table R1. Summary of solid-state TTA-UC performances reported to date. Note that the maximum value of Φ_{UC} is 50%.

Excitation wavelength (nm)	UC emission wavelength (nm)	Φ_{UC} (%)	I_{th} (mW cm^{-2})	Reference
530	435	3	5.6	15
514.5	430	11	20	16
808	612	0.62	12	17
808	612	0.26	17	17
808	612	0.11	26	17
532	470	0.008	10.5	18
532	440	0.1	1.7	18
532	440	0.14	2.5	18
938	580	0.22	10000	19
532	440	2.5	6	20
532	440	5–10	5	21
532	440	0.22	326	22
532	440	1.0	531	22
532	440	0.022	450	22
640	514	0.29	35.9	23
532	440	0.64	2.5	24
532	435	3	49	25
785	450	0.00045	1000	26
532	440	4.5	195	27
532	436	0.022	200	28
532	427	0.31	110	28
532	427	0.92	52	28
542	434	13.4	2.1	29

532	440	5.7	39.1	30
635	470	3.1	35	31
440	390–393	4.3	13.8	32
808	608	2.2	10	33
808	608	0.85	8	33
445	394	1.3	44	34
721	573	1.5 (corrected)	1100	35

3. The significant reduction in PLQY of unsubstituted DHI in crystals compared to solution is attributed to energy traps promoting singlet exciton deactivation. However, molecular geometry changes upon crystallization could also enhance intersystem crossing to the triplet state, an alternative deactivation pathway not considered. The authors are requested to measure phosphorescence in crystals at low temperatures to confirm this channel.

We appreciate this comment. We measured the phosphorescence spectra of recrystallized DHI at both 77 K and room temperature (Figure R4). The spectrum at 77 K showed no additional emissive features and closely resembled that at room temperature, which does not support an intersystem-crossing-mediated deactivation channel in DHI crystals.

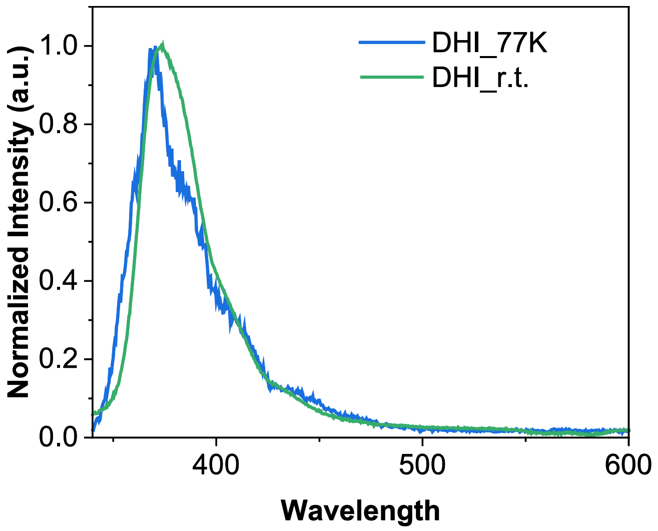

Figure R4. Emission spectra of DHI crystal at 77 K and room temperature (λ_{ex} = 300 nm). The emission spectrum of DHI at 77 K did not show new peaks corresponding to its phosphorescence.

4. The choice of Ir(ppy)3 as a donor is flawed due to strong overlap between acceptor emission and donor absorption, likely causing significant BET and emission quenching. The authors should measure PLQY of acceptors with and without the donor in solution and crystals to evaluate BET effects.

We acknowledge the comment on the spectral overlap between the acceptor emission and the donor absorption, which could lead to resonant singlet back energy transfer (BET) from the acceptor to the donor and reabsorption of emitted photons by the donor. Our choice of $Ir(ppy)_3$ as the donor is based on its favorable triplet energy levels, excellent stability, and commercial availability. Additionally, Ir-based complex is widely used as a donor in visible-to-UV triplet-triplet annihilation upconversion (TTA-UC) systems (*Angew. Chem. Int. Ed.*, **2023**, *135*, e202301506), allowing our results to be meaningfully compared with previous studies.

Tables of PLQY in solution and in crystalline solids are shown in Table R2. In the case of *i*Bu-DHI discussed in the manuscript, as the fluorescence decay profiles did not change much, the resonant BET process is less significant. Therefore, we concluded that the reabsorption mainly contributes to the reduction. We agree with the importance of managing the spectral overlap between the donor absorption and acceptor emission, which is one of our future works.

Table R2. PLQY of the THF solution at the concentration of 10 mM and spin-coat films of DHI derivatives with and without $Ir(ppy)_3$.

	Φ_F in solution (%)		Φ_F in the solid state (%)	
	without $Ir(ppy)_3$	with $Ir(ppy)_3$	without $Ir(ppy)_3$	with $Ir(ppy)_3$
Me-DHI	89	33	61	38
*i*Bu-DHI	91	38	92	44
2-EtBu-DHI	74	29	11	15

5. It is unclear why the PL lifetime remans unchanged despite the BET-induced quenching. The authors should clarify this inconsistency, as this quenching typically affects both PLQY and lifetime.

We appreciate this comment. The small change in the fluorescence lifetime indicates that the effect of BET is not significant in *i*Bu-DHI (Figure R5b, S17b, 5.3% based on the equation $\Phi_{BET}= 1- \tau_F / \tau_{F,0}$, *ChemPhotoChem*, **2020**, *4*, 5271–5278).The reduction of PLQY of *i*Bu-DHI from 92% to 44% is mainly due to the reabsorption by $Ir(ppy)_3$. This small BET contribution is primarily because of the intrinsically small molar absorption coefficient of $Ir(ppy)_3$ (ε_{370} = 1.3×10^4 M^{-1} cm^{-1}) around the acceptor emission peak. This value is similar to that of the typical sensitizer PtOEP (ε_{410} ~1.5×10^4 M^{-1} cm^{-1}) paired with 9,10-diphenylanthracene system. In addition, the short fluorescence lifetime (~2 ns) of the DHI derivatives may make BET less efficient. Nanoscale phase separation may also reduce the chance of BET, but we could not investigate it due to the resolution limit of our experiments. We

have inserted these comments in the revised manuscript (page 9 line 7).

"This small BET contribution is primarily ascribed to the intrinsically small molar absorption coefficient of Ir(ppy)3 (ε_{370} ~ 1.3 × 10^4 M^{-1} cm^{-1}) around the acceptor emission peak (Figure S7). In addition, the short fluorescence lifetime (~2 ns) of the DHI derivatives would have made the BET less efficient (Figure S17, S18). In general, nanoscale phase separation could result in decrease TET and BET, but we could not confirm such sample inhomogeneity due to the limitation of the SEM-EDX experiments as mentioned later."

6. FL decays profiles were provided only for iBu-DHI spin-coated films, with no data for other derivatives.

We appreciate this suggestion. We have added the fluorescence decays of Me-DHI and 2-EtBu-DHI to the supporting information (Figure R5, S17).

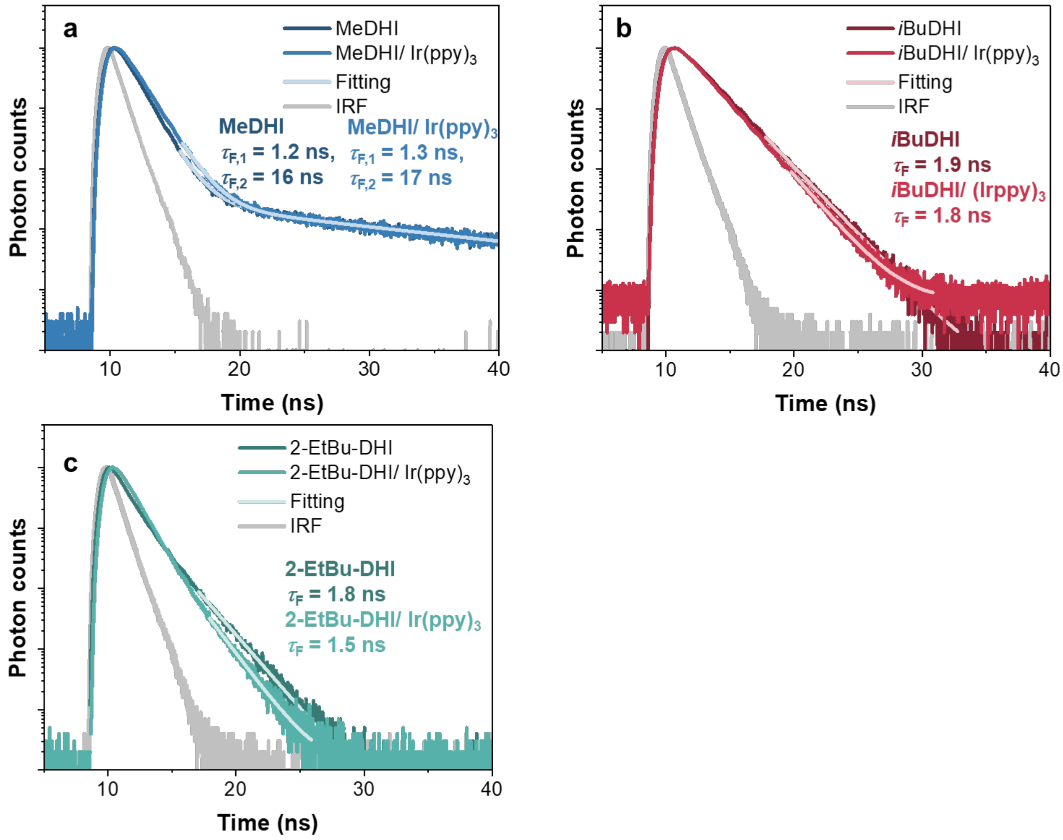

Figure R5. Fluorescence decay profiles of spin-coated films of **a** Me-DHI, **b** *i*Bu-DHI, and **c** 2-EtBu-DHI with and without Ir(ppy)3 (molar ratio for preparation; DHI derivative: Ir(ppy)3 = 100 : 1). Photo excitation at 370 nm. The decay of Me-DHI was fitted with a bi-exponential function, and those for *i*Bu-DHI and 2-EtBu-DHI were fitted with a mono-exponential function. The light gray line represents the instrumental response function (IRF) of the instrument.

7. The UC emission spectra in Fig. S9 are truncated at 350 nm, showing only partial spectra (e.g., only half in Fig. S9a), limiting analysis. The authors should measure spectra from at least 280 nm using quartz optics to capture the full UC emission range. This may have also led to unreliable UCQY estimation as detailed in Sect. 8 (SI), since only part of UC spectra was measured. Note also that the UC spectrum in Fig. S15a abruptly changes at 350nm.

We appreciate the reviewer's comments. We installed quartz optics to obtain the spectra shorter than 350 nm and repeated the experiments. The results are inserted into the revised supporting information (Figure R6–8, S9–11). For DHI, although the spectra from 300 nm excitation were measured, the Φ_{UC} seems to be underestimated because the UCPL intensity decreases over time under high excitation power (Figure R9, S13), which is possibly due to a dimerization reaction that has been reported for unsubstituted DHI (*Bull Chem. Soc. Jpn.*, **1982**, *55*, 1310–1312).

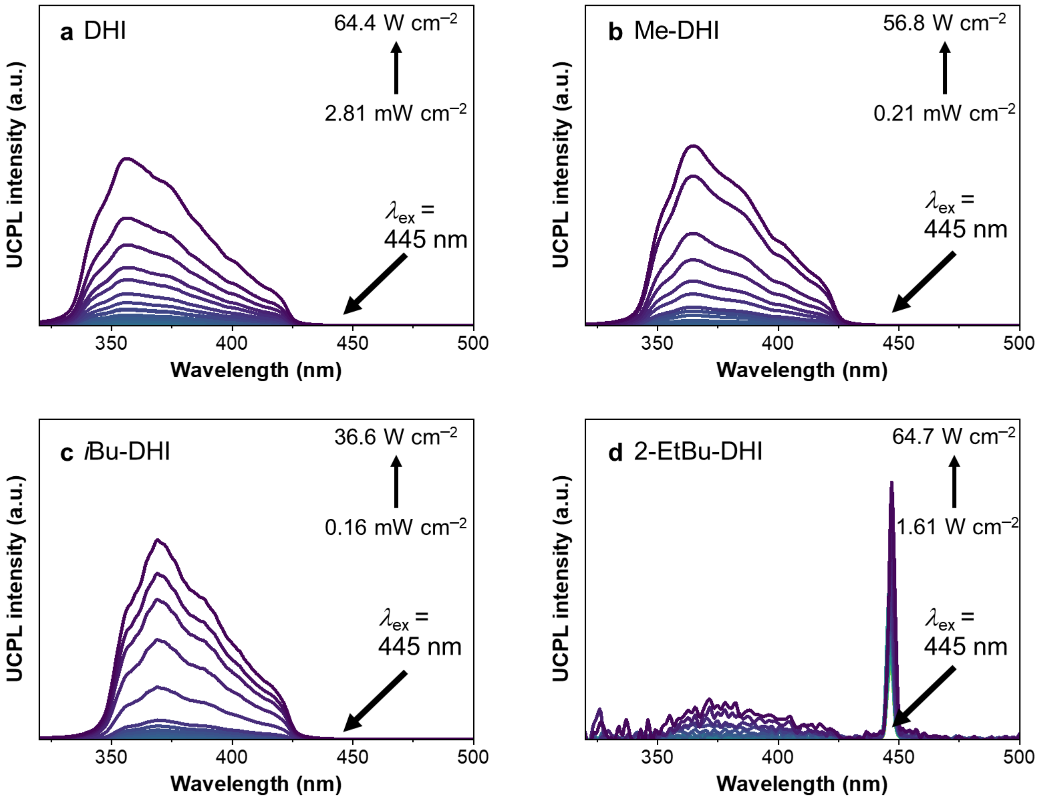

Figure R6. UCPL spectra at different excitation intensities of **a** DHI, **b** Me-DHI, **c** *i*Bu-DHI, and **d** 2-EtBu-DHI at an acceptor concentration of 1 mM with 50 μM $Ir(ppy)_3$ in deaerated THF. Excitation light was removed with a 425 nm short-pass filter. Aggregation of 2-EtBu-DHI was observed upon 445 nm laser excitation, which might be the reason for the negligible UC emission.

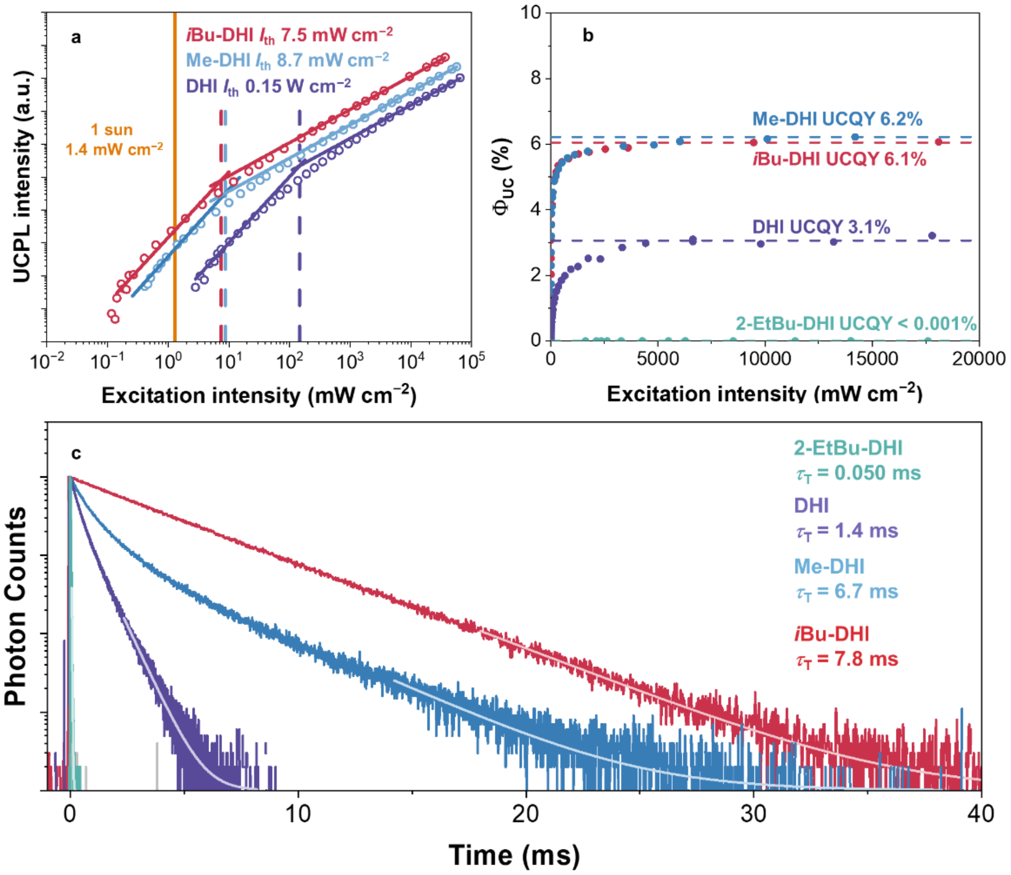

Figure R7. a Plots of excitation intensity-dependent UC emission intensity, **b** Φ_{UC} at various excitation intensities in deaerated THF. The yellow line represents the intensity of sunlight. **c** UC emission decay curves of DHI (purple), Me-DHI (blue), *i*Bu-DHI (red), and 2-EtBu-DHI (green) in deaerated THF ([DHI derivatives: Ir(ppy)$_3$] = 1 mM : 50 μM, λ_{ex} = 445 nm, λ_{dt} = 370 nm). Excitation light was removed with a 425 nm short-pass filter. The light gray line is the instrumental response function (IRF) of the instrument. The fitting curves for the tail part of the UC decays were obtained by the known relationship,

$$I_{UC}(t) \propto \exp\left(\frac{-t}{\tau_{UC}}\right) = exp\left(\frac{-2t}{\tau_T}\right) \qquad (1)$$

, where τ_{UC} and τ_T are the UC emission lifetime and acceptor triplet lifetime, respectively.

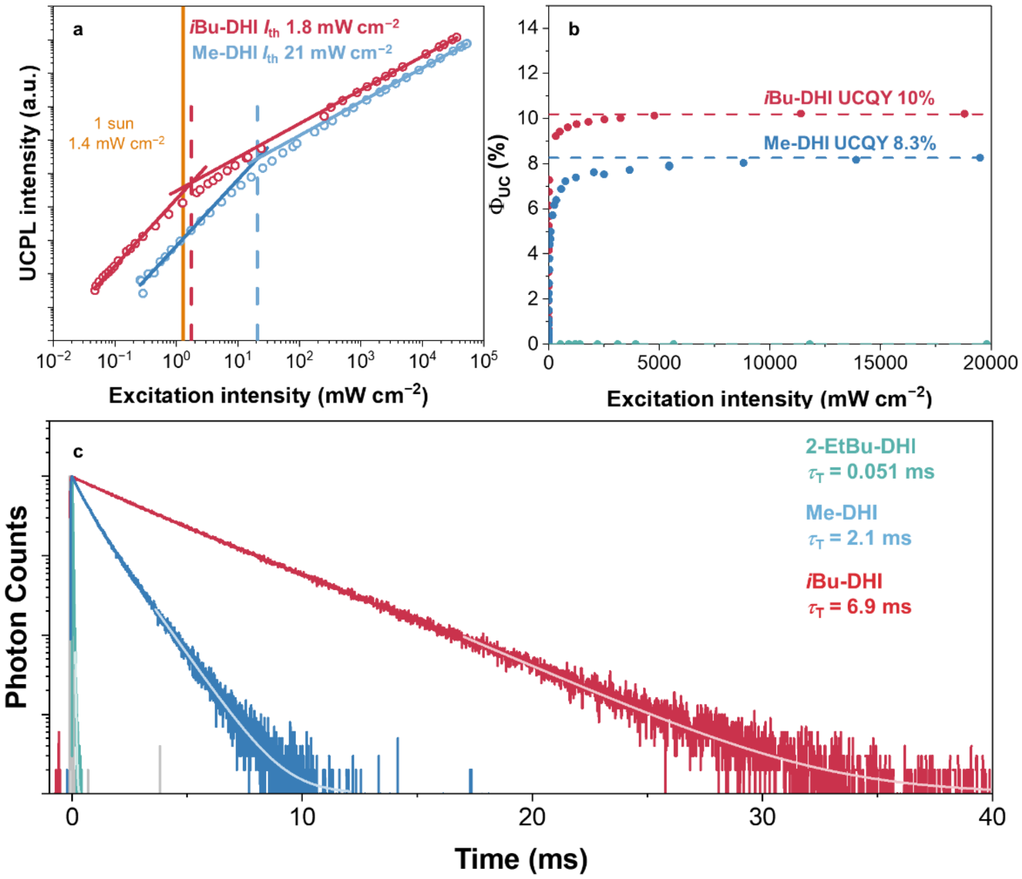

Figure R8. a Plots of excitation intensity-dependent UC emission intensity, **b** Φ_{UC} at various excitation intensities in deaerated THF. The yellow line represents the intensity of sunlight. **c** UC emission decay curves of Me-DHI (blue), *i*Bu-DHI (red), and 2-EtBu-DHI (green) in deaerated THF ([DHI derivatives: $Ir(ppy)_3$] = 10 mM : 100 µM, λ_{ex} = 445 nm, λ_{dt} = 370 nm). Excitation light was removed with a 425 nm short-pass filter. The light gray line is the instrumental response function (IRF) of the instrument. The fitting curves for the tail part of the UC decays were obtained using the known relationship according to equation (1).

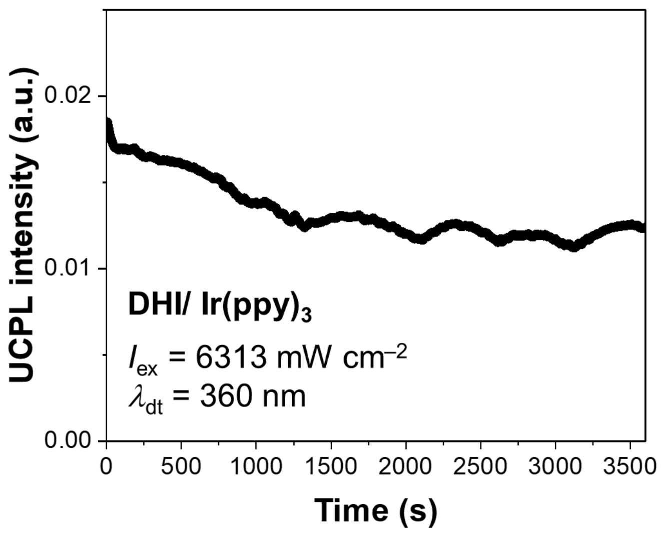

Figure R9. Time-dependent UCPL intensity in deaerated THF ([DHI] = 1 mM, [Ir(ppy)$_3$] = 50 μM) under continuous laser irradiation at 445 nm for 1 h (λ_{ex}= 445 nm, λ_{dt}= 360 nm, I_{ex} = 6.3 W cm^{-2}). We observed a gradual decrease in the UC emission intensity, likely arising from reactions such as photodimerization reported under UV irradiation (*Bull Chem. Soc. Jpn.*, 1982, *55*, 1310-1312). To minimize this effect, the spectra were recorded soon after the irradiation and with minimal exposure time less than 0.5 s.

8. The UC QY of unsubstituted DHI, which has the highest Ith, saturates at a lower excitation intensity (~2500 mW/cm^2) compared to Me-DHI and iBu-DHI (>5000 mW/cm^2), raising concerns about data consistency.

We thank the reviewer for this comment. The UC emission intensity of the DHI is unstable at high excitation intensity (Figure R9, S13), leading to a deviation from the typical trend.

9. Fig. S15a labels the Y-axis as "normalized intensity," but the spectra are clearly not normalized.

We appreciate the referee for pointing out this. We have normalized the spectrum intensity at 591 nm because the sensitizer's absorption is negligible around this region. We have revised the caption as follows (Figures R10 and S19).

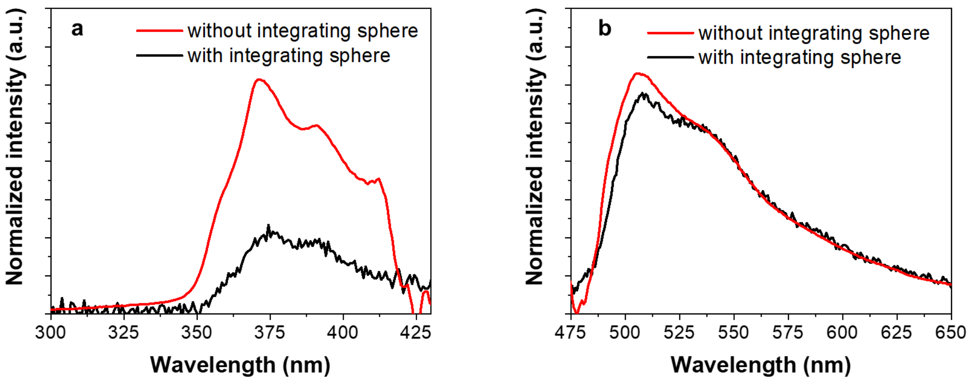

Figure R10. **a** Normalized UC emission and **b** phosphorescence spectra of the spin-coated film of *i*Bu-DHI doped with Ir(ppy)$_3$ detected with (black) and without (red) integrating sphere (I_{ex} = 4260 mW cm^{-2}). The spectra were normalized to the intensity at 591 nm.

10. Regarding the possible 2-EtBu-DHI aggregation, suggested by Fig. S9d data. The authors are requested to test lower acceptor concentrations, such as 1 mM (as used for DHI) or even less, and include UC data for 2-EtBu-DHI at these concentratio
s in Fig. S10a-c.

We appreciate this comment. We have tested 2-EtBu-DHI (1 mM) with Ir(ppy)$_3$ where the ^{1}H-NMR did not provide a clear signal from aggregates (Figure R11). The UC emission observed for 2-EtBu-DHI at this concentration was very weak (Figure R7, Φ_{UC} < 0.001%). Even at this concentration, unusual excited-state quenching channels seem to be present in this system. We have included the UC properties in the revised manuscript.

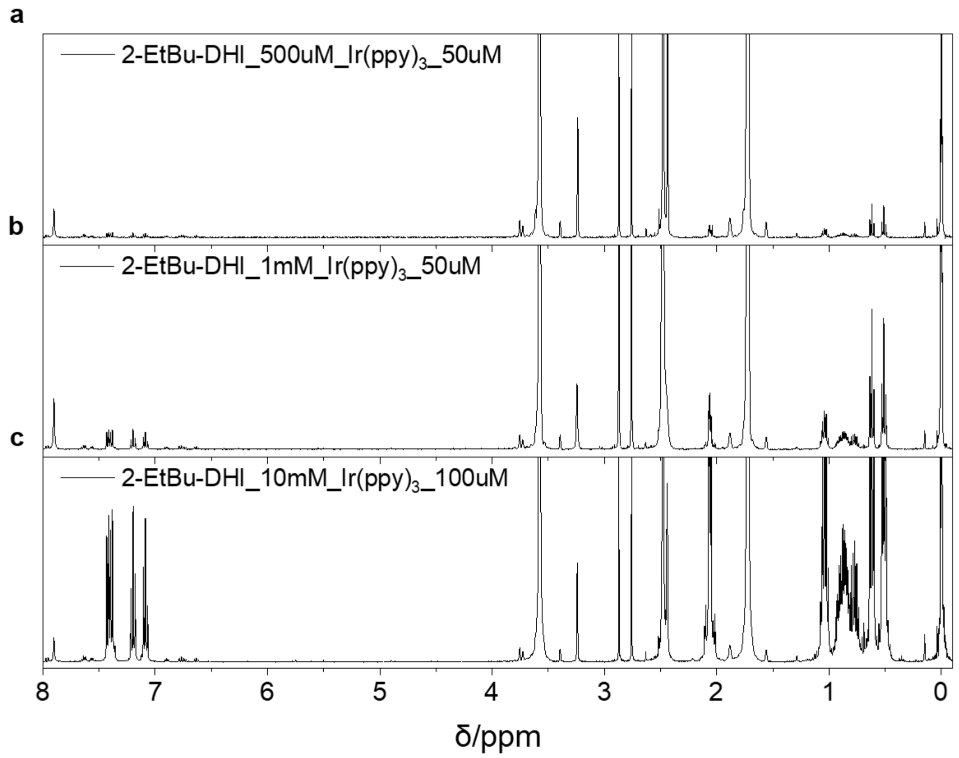

Figure R11. ^{1}H-NMR (400 MHz, THF-*D8*, TMS) spectra of the **a** 500 μM: 50 μM mixture, **b** 1 mM: 50 μM mixture, and **c** 10 mM: 100 μM mixture of 2-EtBu-DHI and $Ir(ppy)_3$.

11. The UC QY of unsubstituted DHI, which has the highest threshold intensity (Ith), saturates at a lower excitation intensity (~2500 mW/cm²) compared to Me-DHI and iBu-DHI (>5000 mW/cm²), raising concerns about data consistency.

We thank the referee for this constructive comment. At high excitation intensity, we observed a gradual decrease in the UC emission of the mixed solution of $Ir(ppy)_3$ and DHI under continuous irradiation. This is possibly due to reactions such as dimerization under UV irradiation (*Bull Chem. Soc. Jpn*, **1982**, *55*, 1310–1312). This reduction in the UC emission intensity led to apparent "saturation" of DHI at around 2500 mW cm^{-2} in the previous manuscript. In the revised manuscript with new measurements, we have optimized the measurement condition for DHI, including exposure time. We have added the following comments to the caption of Figure S13 (Figure R9).

"We observed a gradual decrease in the UC emission intensity, likely arising from reactions such as photodimerization reported under UV irradiation (*Bull Chem. Soc. Jpn*, **1982**, *55*, 1310–1312). To minimize this effect, the spectra were recorded soon after irradiation, with minimal exposure time (< 0.5 s)."

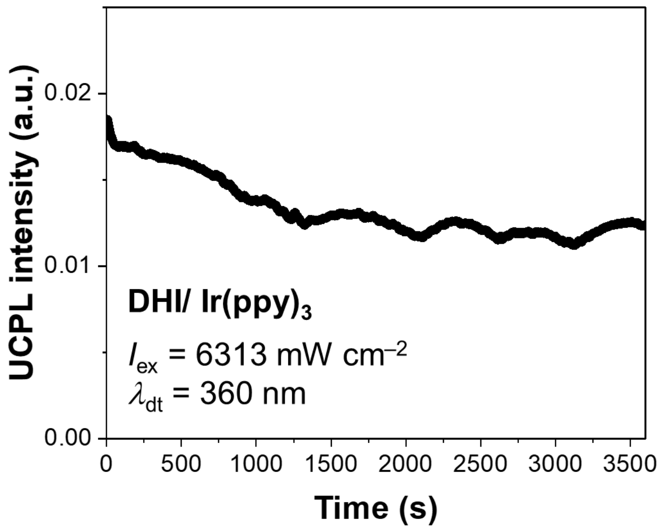

Figure R9. Time-dependent UCPL intensity in deaerated THF ([DHI] = 1 mM, $[Ir(ppy)_3]$ = 50 μM) under continuous laser irradiation at 445 nm for 1 h (λ_{ex}= 445 nm, λ_{dt}= 360 nm, I_{ex} = 6.3 W cm^{-2}). We observed a gradual decrease in the UC emission intensity, likely arising from reactions such as photodimerization reported under UV irradiation (*Bull Chem. Soc. Jpn.*, 1982, *55*, 1310-1312). To minimize this effect, the spectra were recorded soon after irradiation, with minimal exposure time (< 0.5 s)."

12. To substantiate their claim of aggregate- or dimer-related excited state quenching in DHI solutions, the authors should conduct acceptor concentration-dependent measurements of spectra, lifetime, and QY.

We appreciate this suggestion. We performed experiments for DHI in THF solutions from 0.01 mM to 1 mM (Figure R12, Table R3) since DHI was undissolved in 10 mM concentration. The peak of the normalized absorption and fluorescence spectra did not change much with concentration. A decrease in fluorescence quantum yield was observed with increasing concentration. But we could not identify the dimer-related quenching process in these measurements as the fluorescence lifetime was similar under these concentrations. Based on the much shorter triplet lifetime of DHI than *i*Bu-DHI and the significant reduction of the fluorescence quantum yield after crystallization, we speculate quenching due to intermolecular interaction during TTA process is more prominent in DHI.

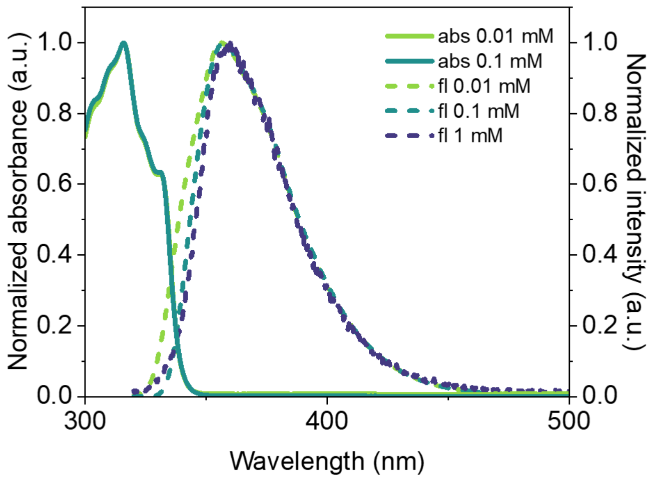

Figure R12. Normalized absorption (solid line) and normalized emission (dashed line) spectra of DHI in THF at various concentrations.

Table R3. Fluorescence quantum yields (Φ_F) and fluorescence lifetimes (τ_F) of DHI in THF.

	Φ_F		τ_F (ns)		
Concentration (mM)	0.2	1.0	0.01	0.2	1.0
DHI	0.96	0.82	2.2	2.0	2.1

13. To enable a fair comparison of DHI derivatives in Table S2, all parameters must be measured at a consistent acceptor concentration (1 mM or lower) and in the absence of aggregation. 2-EtBu-DHI must be included in the analysis as well.

We appreciate this comment. We have measured the UC properties at a concentration of 1 mM for each acceptor and have added to the supporting information (Figure R7, S10). The τ_T values were longer than those observed at 10 mM, possibly due to suppressed triplet quenching (*J. Am. Chem. Soc.*, **2023**, *145*, 22168–22175), and the I_{th} values became lower. However, the UCQY was not maximized at 1 mM due to the low triplet transfer efficiencies of Me-DHI and *i*Bu-DHI (Φ_{TET} < 70% for MeDHI and Φ_{TET} < 50% for *i*Bu-DHI).

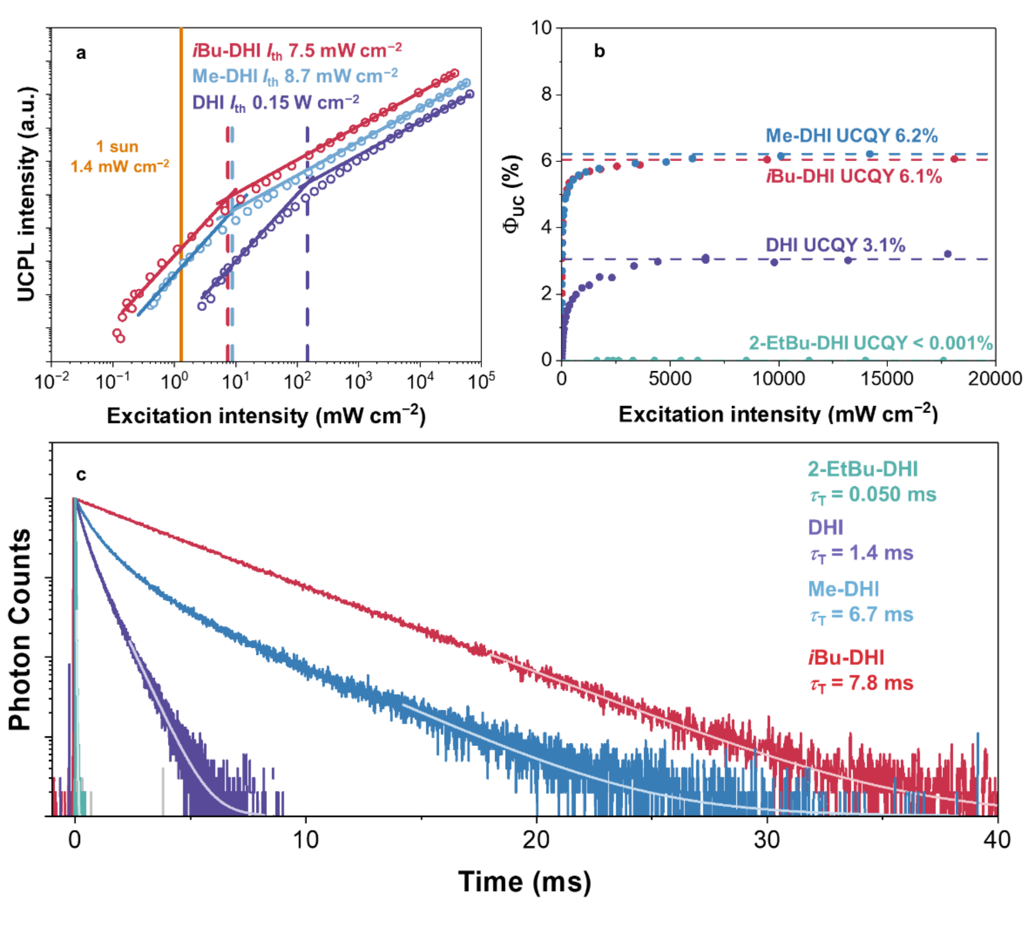

Figure R7. **a** Plots of excitation intensity-dependent UC emission intensity, **b** Φ_{UC} at various excitation intensities in deaerated THF. The yellow line represents the intensity of sunlight. **c** UC emission decay curves of DHI (purple), Me-DHI (blue), *i*Bu-DHI (red), and 2-EtBu-DHI (green) in deaerated THF ([DHI derivatives: $Ir(ppy)_3$] = 1 mM : 50 µM, λ_{ex} = 445 nm, λ_{dt} = 370 nm). Excitation light was removed with a 425 nm short-pass filter. The light gray line is the instrumental response function (IRF) of the instrument. The fitting curves for the tail part of the UC decays were obtained by the known relationship,[4]

$$I_{\mathrm{UC}}(t) \propto \exp\left(\frac{-t}{\tau_{\mathrm{UC}}}\right) = exp\left(\frac{-2t}{\tau_{\mathrm{T}}}\right) \qquad (1)$$

, where τ_{UC} and τ_{T} are the UC emission lifetime and acceptor triplet lifetime, respectively.

14. Fig. 3 lacks data for unsubstituted DHI crystalline films, which is essential for a comprehensive comparison.

We appreciate the comment. Due to the poor solubility of DHI (~ mM at maxima), we could not fabricate the spin-coated film under the same conditions used for other DHI derivatives ([Acceptor]= 100 mM, [Donor]= 1 mM). We have tried to measure UCPL in drop-cast films, but we could not detect the UC emission from the sample, possibly due to the low PLQY. We have included the following sentence in the manuscript as the original manuscript did not provide any comments on the DHI film (page 8 line 17).

"In contrast, a spin-coated DHI film did not exhibit detectable UC emission, likely due to its low Φ_{F}."

15. The claim that "deactivation of excited states by exciton trap sites in crystalline DHI systems is avoided by de-trapping via thermal excitation" is highly questionable. If true, the PLQY should decrease at lower temperatures due to insufficient thermal energy for de-trapping. This requires verification.

We thank the referee for this constructive comment. As the referee mentioned, if the trapping results in nonradiative decay, Φ_{F} drop should be observed at low temperature. We measured the Φ_{F} at 77K, which showed increased QY values. This result indicates that the detrimental nonradiative process is a thermally activated process and our previous explanation of trapping and de-trapping is not applicable to the current materials. We have removed the description of de-trapping from the manuscript.

Table R4. Fluorescence quantum yield in the solid state at room temperature and 77K (λ_{ex} = 320 nm).

	$\Phi_{\mathrm{F,RT}}$ (%)	$\Phi_{\mathrm{F,77K}}$ (%)
DHI	9.2	44
*i*Bu-DHI	66	85

16. Although the authors identify *i*Bu-DHI as the best crystalline-state acceptor to date, they should also emphasize its practical limitations, namely, the difficulty in controlling crystal size, grain boundaries, and ensuring consistent UC and PL performance across batches.

We appreciate this comment. As the referee mentions, there are several practical limitations, as the performance depends on the fabrication process. Optimization of hybrid structures via processes such as controlled crystallization (*J. Mater. Chem. C*, **2023**, *11*, 1678–1683) may improve the UC performance with controlled crystal size and grain boundaries. We added the sentence to the revised manuscript (page 14 line 23)

"Note that while the significantly low I_{th} values and high Φ_{UC} in the films formed by the spin coating or drop casting are attractive in terms of low-cost fabrications, these processing are not suitable to control crystal sizes and grain boundaries necessary to minimize batch or processing condition-dependence in UC and PL performance. Optimization of hybrid structures via processes (*J. Mater. Chem. C*, **2023**, *11*, 1678–1683) such as controlled crystallization may improve the UC performance with controlled crystal size and grain boundaries."

17. It is unclear why the average phosphorescence lifetime of the $Ir(ppy)_3$ donor increases in the iBu-DHI film (Table S3), whereas in other DHI films it decreases significantly due to TET.

In addition to the rapid initial triplet energy transfer, reabsorption and triplet back energy transfer potentially contribute to the phosphorescence decay profiles in the sensitizer-doped films. Since the average phosphorescence lifetime includes long-lived, acceptor triplet components, using the average phosphorescence lifetime is not suitable for analyzing the TET process (Figure R13, S21, Table R5, S2). The difference in the long-lived component may arise from differences in the structure of acceptor packing or donor–acceptor interfaces, and in UC quantum yields that contribute to photon recycling.

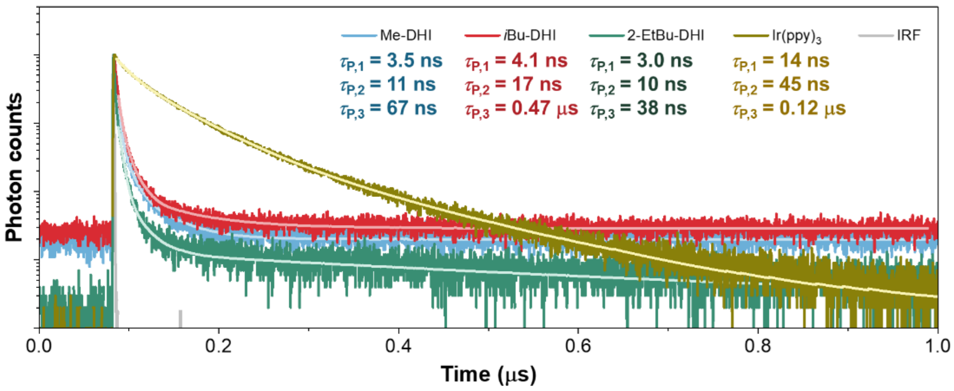

Figure R13. Time-resolved phosphorescence decay curves of the spin-coated films of $Ir(ppy)_3$ in the presence of Me-DHI (blue), *i*Bu-DHI (red), and 2-EtBu-DHI (green), along with a reference drop-cast film of $Ir(ppy)_3$ alone (yellow). Decay profiles were recorded upon excitation at 405 nm, with detection at 515 nm. The instrumental response function (IRF) is also shown (gray).

Table R5. Phosphorescence lifetime parameters obtained from multi-exponential fitting of the time-resolved decay profile of the spin-coated films of $Ir(ppy)_3$ in the presence of Me-DHI, *i*Bu-DHI, and 2-EtBu-DHI, along with a reference drop-cast film of $Ir(ppy)_3$ alone. Decay profiles were recorded upon excitation at 405 nm, with detection at 515 nm. The table summarizes the fitted lifetimes (t_i), corresponding pre-exponential factor (A_i), fractional intensity (f_i), and the calculated average lifetime $\langle \tau \rangle$ from equations (6–8).

	$\langle \tau \rangle$ (ns)	τ_1 (ns)	τ_2 (ns)	τ_3 (ns)	A_1	A_2	A_3	f_1 (%)	f_2 (%)	f_3 (%)
$Ir(ppy)_3$/ Me-DHI	11.6	3.52	11.1	66.9	1722	244.8	13.49	63	28	9
$Ir(ppy)_3$/ *i*Bu-DHI	136	4.09	17.0	474	585.0	31.33	2.363	59	13	28
$Ir(ppy)_3$/ 2-EtBu-DHI	8.94	2.99	10.3	37.6	1597	150.8	24.40	66	21	13
$Ir(ppy)_3$	58.7	14.3	45.1	121	846.9	1006	153.1	16	60	24

$$I(t) = \sum_{i=1}^{n} A_i \exp\left(-\frac{t}{\tau_i}\right) \quad (6)$$

$$\langle\tau\rangle = \frac{\sum_{i=1}^{n} A_i {\tau_i}^2}{\sum_{i=1}^{n} A_i \tau_i} \quad (7)$$

$$f_i = \frac{A_i \tau_i}{\sum_{i=1}^{n} A_i \tau_i} \quad (8)$$

18. What are the UCQY and Ith at t=0 min for the spin-coated iBu-DHI/$Ir(ppy)_3$ film prepared under air-saturated conditions (Fig. S19)?

We appreciate this comment. It was difficult to evaluate UCQY and I_{th} at t = 0 min under air-saturated conditions because the weak UCPL intensity required approximately 1 minute of exposure time. We have included the following comments (see Figure R14 and S23).

"It was difficult to evaluate UCQY and I_{th} in air at t = 0 min under air-saturated conditions because the weak UCPL intensity required approximately 1 minute of exposure time."

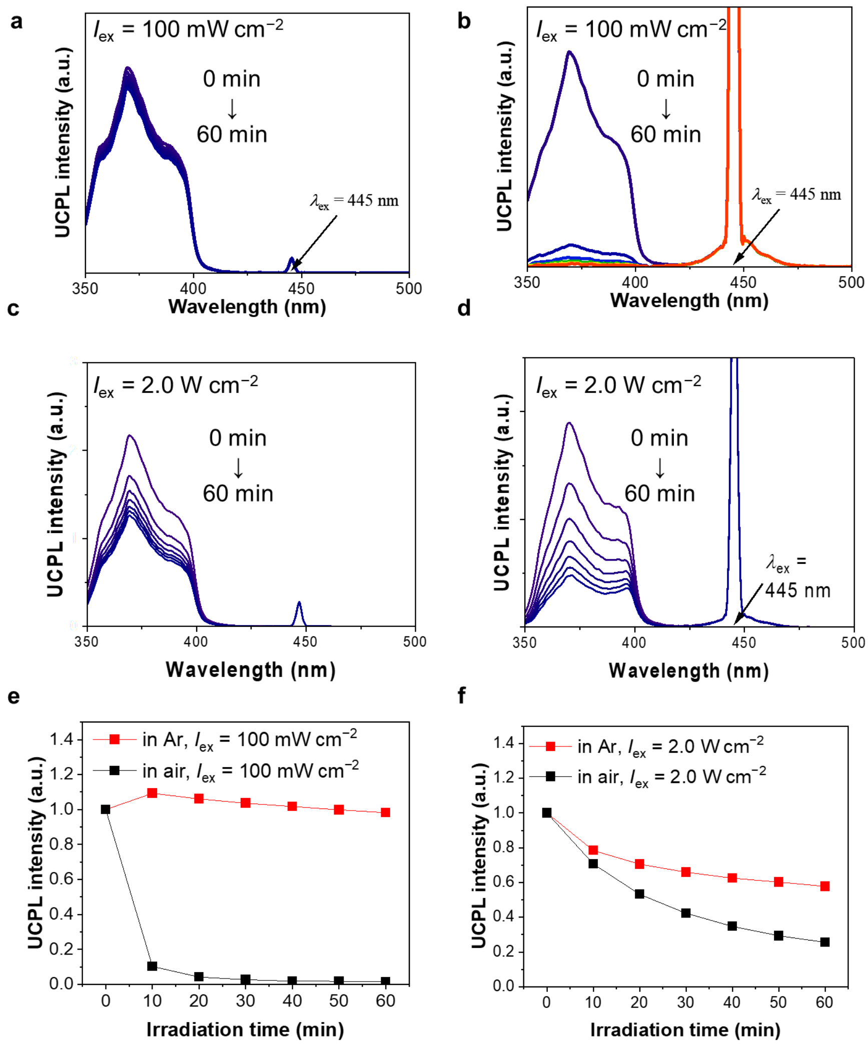

Figure R14. Time-dependent UC emission spectra of a spin-coated film of *i*Bu-DHI/Ir(ppy)$_3$ (molar ratio for preparation; *i*Bu-DHI : Ir(ppy)$_3$ = 100 : 1) prepared and measured **a** in Ar and **b** in air were recorded under excitation at 100 mW cm^{-2}. **c** The results in Ar and **d** in air were recorded under 2.0 W cm^{-2}. Excitation light was removed with a 400 nm short-pass filter. Time-dependent UC emission intensity with (red) and without (black) encapsulation **e** under excitation intensity at 100 mW cm^{-2} and **f** at 2.0 W cm^{-2} (λ_{dt} = 370 nm). It was difficult to evaluate UCQY and I_{th} in air at t = 0 min under air-saturated conditions because the weak UCPL intensity required approximately 1 minute of exposure time.

19. The authors claim that the $Ir(ppy)_3$ donor is homogeneously distributed within DHI microcrystals without segregation, based on SEM and SEM-EDX data. However, these techniques cannot distinguish whether the donor is embedded within the crystals or merely on their surface. Additional evidence is needed, or the statement should be rephrased accordingly.

We thank the reviewer for this comment and agree that rephrasing is necessary. We have added the following sentence to the manuscript (page 11 line 8).

"It is difficult to determine from SEM resolution whether the donors are located within the crystal or on its surface, or whether they are molecularly dispersed. However, the observation of high Φ_{UC} and low I_{th} in these crystal samples suggests that the donors are uniformly dispersed within the acceptor crystal, thereby enabling efficient triplet sensitization."

In addition, because the current results do not clarify whether the sensitizer is molecularly dispersed, we mentioned only micrometer-scale segregation, which we could confirm from the data (page 11 line 6).

"Ir and N atoms of $Ir(ppy)_3$ are homogeneously distributed in these microcrystals without micrometer-scale segregation, unlike the commonly observed inhomogeneous solid-state sensitizer–annihilator mixtures (*Phys. Chem. Chem. Phys.* **2012**, *14*, 4322–4332)."

20. The authors state that "the drastic reduction of the Ith value for iBu-DHI/Ir(ppy)3 implies fast triplet energy diffusion in the crystal", but this is not necessarily true. A lower Ith could also result from significantly enhanced TET from donor to acceptor. To distinguish between TET and diffusion contributions, the authors should quantify TET in spin-coated DHI films, for example, by conducting donor phosphorescence quenching experiments with increasing acceptor concentration in an inert polymer matrix.

We appreciate the comments on TET efficiency. Unlike the TET in neat films, where triplet energy migrates among neighboring acceptors, the TET process in a polymer matrix depends on molecular diffusion and collision. We think it is better to compare the phosphorescence lifetimes or quantum yields of the donor-acceptor films with those of neat solid $Ir(ppy)_3$, as we cannot rule out partial nanoscale segregation. By using the phosphorescence quantum yield of $Ir(ppy)_3$ in the neat solid (Φ_P = 9.4%), the lower limit of TET efficiency of Me-DHI, *i*Bu-DHI, and 2-EtBu-DHI was estimated to be 59%, 71%, and 93%, respectively (Table R6, S3).

Table R6. Phosphorescence quantum yield of the neat solid of $Ir(ppy)_3$ and spin coated DHI films with $Ir(ppy)_3$.

	Φ_p (%)	Φ_{TET} (%)
$Ir(ppy)_3$	9.4	-
$Ir(ppy)_3$/Me-DHI	3.9	59
$Ir(ppy)_3$/*i*Bu-DHI	2.7	71
$Ir(ppy)_3$/2-EtBu-DHI	0.63	93

When the phosphorescence quantum yield of the THF solution of $Ir(ppy)_3$ (100 μM) was used as a reference (Φ_p = 88%), Φ_{TET} values of Me-DHI, *i*Bu-DHI, and 2-EtBu-DHI UC films are calculated as 96%, 97%, and 99%, respectively. In either case, the difference in TET efficiency between *i*Bu-DHI and 2-EtBu-DHI does not account for the 3 orders of magnitude difference in I_{th}, indicating the contribution of triplet diffusion in the crystal discussed in section 15.

We have inserted the following sentence into the manuscript, and the details have been added to the supporting information (section 15).

"By using the phosphorescence quantum yield of $Ir(ppy)_3$ in the neat solid (Φ_p = 9.4%), the lower limit of TET efficiency of Me-DHI, *i*Bu-DHI, and 2-EtBu-DHI was estimated to be 59%, 71%, 93%, respectively (Table S3)"

To further investigate the contribution of triplet diffusion, the TET and TTA reaction times were theoretically estimated using the method reported by Sato and Shigeta et. al.. (*J. Phys. Chem. Lett.*

2018, *9*, 6638–6643) , Figure R15, R16, R17, 4, S28, S29, Table R7, S7). Thanks to sufficient π-orbital overlap between the edges of the π-planes, a moderate electronic coupling strength is realized for *i*Bu-DHI, providing a 4-order-of-magnitude faster TET reaction time (1.25 μs and 4.20 × 10^4 μs or 2.85 × 10^4 μs). The TTA reaction times in the same directions were calculated to be 4.54 ns and 8.09 × 10 ns or 1.10 × 10^6 ns, respectively (Figure R15, R16, Table S7). While the current results cannot rule out the potential effect of the nanoscale donor-acceptor interface structures that we could not clarify due to experimental limitations, the calculated values explain the three orders of magnitude difference between *i*Bu-DHI and 2-EtBu-DHI in I_{th} inversely proportional to the rate constants for TTA. Note that the TET and TTA reaction rates between Me-DHI and *i*Bu-DHI were similar (Figure R16, Table S7), which is also consistent with the I_{th} difference explained mainly by the triplet lifetime. Based on both phosphorescence decay profiles and theoretical calculations, we concluded that the difference in triplet diffusion rate explains the significant difference in I_{th} between *i*Bu-DHI and EtBu-DHI. We have revised the paragraph as follows, and detail of the calculations were inserted in the supporting information.

"To elucidate the relationship between the structure and UC performance, theoretical calculations of the TET and TTA rates were conducted for single-crystal structures of *i*Bu-DHI and 2-EtBu-DHI. The X-ray diffraction patterns of the single crystals and those of the spin-coated films were similar (Figure 3f), and we evaluated the intermolecular geometries of the dimers in crystals (Figure 4e,f). In the case of the *i*Bu-DHI crystal, the closest atom–atom distance of the DHI units packed in the crystal structure is 0.4 nm (Figure 4e), which is much shorter than that of the 2-EtBu-DHI crystal (0.7 nm), the latter forming parallel-displaced stacking (Figure 4f). The difference in the electronic coupling matrix elements in these packing structures, and the TET and TTA reaction times were theoretically estimated using the *ab initio* and DFT/TD-DFT calculations, followed by the methods similar to those of Sato and Shigeta et. al. (*J. Phys. Chem. Lett.* **2018**, *9*, 6638–6643, Figure S28, S29, Table S7). The direction giving the fastest reaction times of TET and TTA in *i*Bu-DHI and 2-EtBu-DHI, which are most likely to contribute to the triplet dynamics, are shown in Figure 4ef. Thanks to sufficient π-orbital interaction between the edges of the π-planes, a moderate electronic coupling strength is realized for *i*Bu-DHI, providing a TET reaction time of 1.25 μs, which is a four orders of magnitude faster than that of 2-EtBu-DHI (4.20 × 10^4 μs for pair ***a***, 2.85 × 10^4 μs for pair ***b***). The TTA reaction times in the same directions were calculated to be 4.54 ns and 8.09 × 10^1 ns (for pair ***a***) or 1.10 × 10^6 ns (for pair ***b***) for *i*Bu-DHI and 2-EtBu-DHI, respectively (Figure S28, S29, Table S7). Although nanoscale donor–acceptor interface structures may affect TET efficiency, the calculated values explain the three orders of magnitude difference in I_{th} between *i*Bu-DHI and 2-EtBu-DHI, which is inversely proportional to the TTA rate constants, often approximated with the triplet diffusion rate constant. Note that the TET and TTA reaction rates between Me-DHI and *i*Bu-DHI were similar (Table S7), which is also

consistent with the I_{th} difference explained mainly by the triplet lifetime ($\propto \tau_T^{-2}$). Therefore, we concluded that the molecular organization of *i*Bu-DHI, with a proper balance of shielded and exposed π-surfaces provided by alkyl chains and adequate molecular distance, contributed to the remarkably low I_{th} and high Φ_{UC}. The performance of the present solid-state Vis-to-UV TTA-UC system could be further improved by optimizing the donor molecular structure and employing a controlled crystallization process."

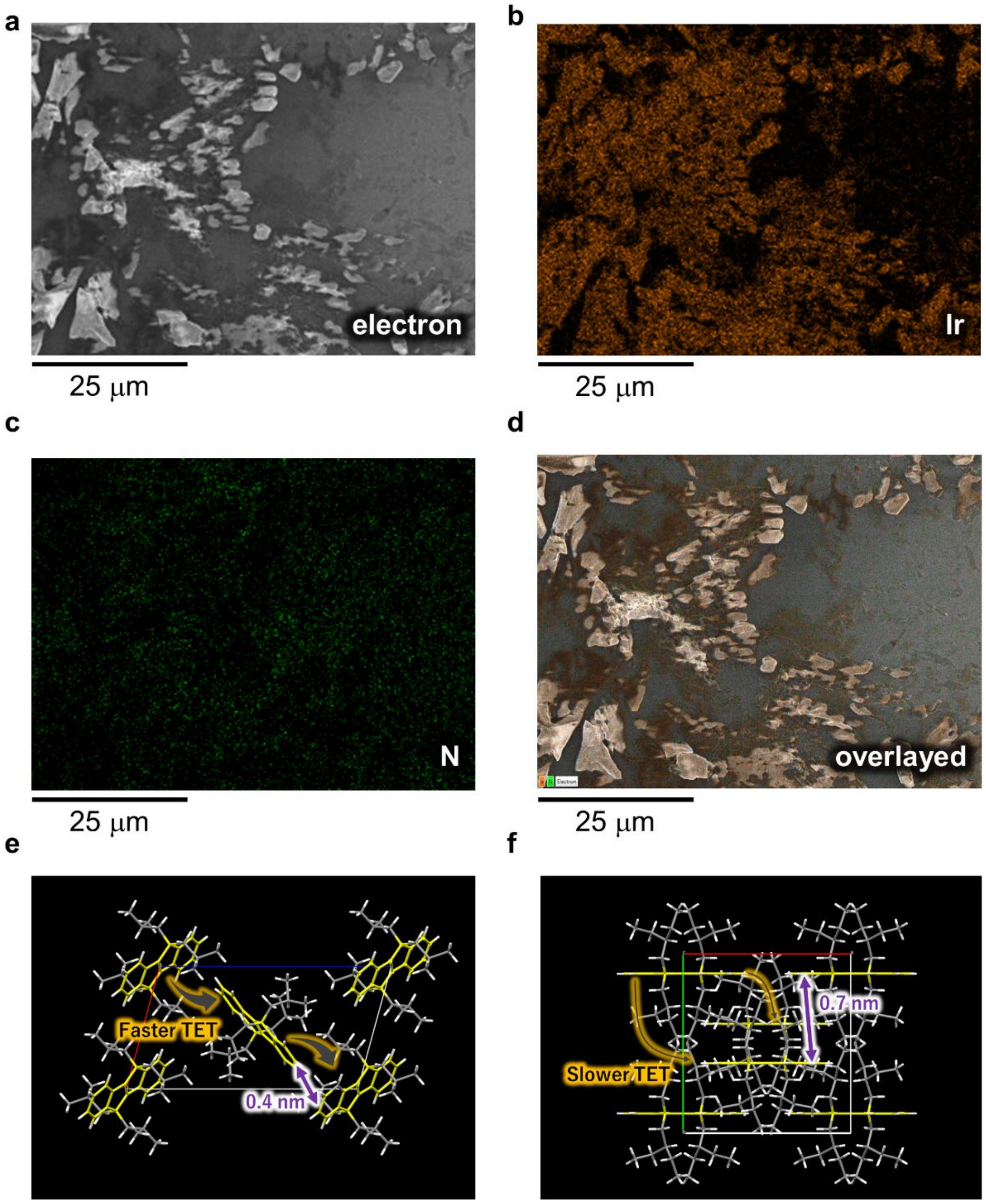

Figure R15. a SEM image, and SEM-EDX mappings of **b** Ir, **c** N, and **d** their overlay for the drop-cast film of *i*Bu-DHI with $Ir(ppy)_3$. Molecular packing structures in the crystal of **e** *i*Bu-DHI and **f** 2-

EtBu-DHI. The purple double arrows indicate the distances of the closest carbon atoms of the adjacent π-planes. The gray arrows indicating the preferred direction of the triplet diffusion theoretically predicted by *ab initio* calculations are also shown (Table R7).

Table R7. Electronic coupling matrix elements ($|T_{DA}|$) and reaction times (τ) of TET and TTA for Me-DHI, *i*Bu-DHI, and 2-EtBu-DHI.

Molecules	pair	$\left\|T_{DA}^{TET}\right\|$(meV)	τ_{TET}(ms)	$\left\|T_{DA}^{TTA}\right\|$ (meV)	τ_{TTA}(ns)	d [a] (Å)
Me-DHI	a	0.0242	6.29×10^1	0.0119	9.67×10^4	4.444
	b	0.0836	5.27	1.4892	6.17	3.830
	c	0.0002	7.39×10^5	0.0027	1.89×10^6	4.304
*i*Bu-DHI	a	0.0026	4.42×10^3	0.0236	1.18×10^4	5.799
	b	0.1570	1.25	1.2038	4.54	3.756
	c	0.0012	2.27×10^4	0.0099	6.73×10^4	6.208
	d	0.0001	3.35×10^6	0.0033	6.08×10^5	7.313
2-EtBu-DHI	a	0.0009	4.20×10^4	0.2802	8.09×10^1	7.043
	b	0.0011	2.85×10^4	0.0024	1.10×10^6	6.800
	c	0.00004	1.91×10^7	0.0015	3.01×10^6	5.093
	d	0.0005	1.52×10^5	0.0061	1.73×10^5	6.014

[a] Nearest C–C distance between pair of π-conjugation of pair molecules.

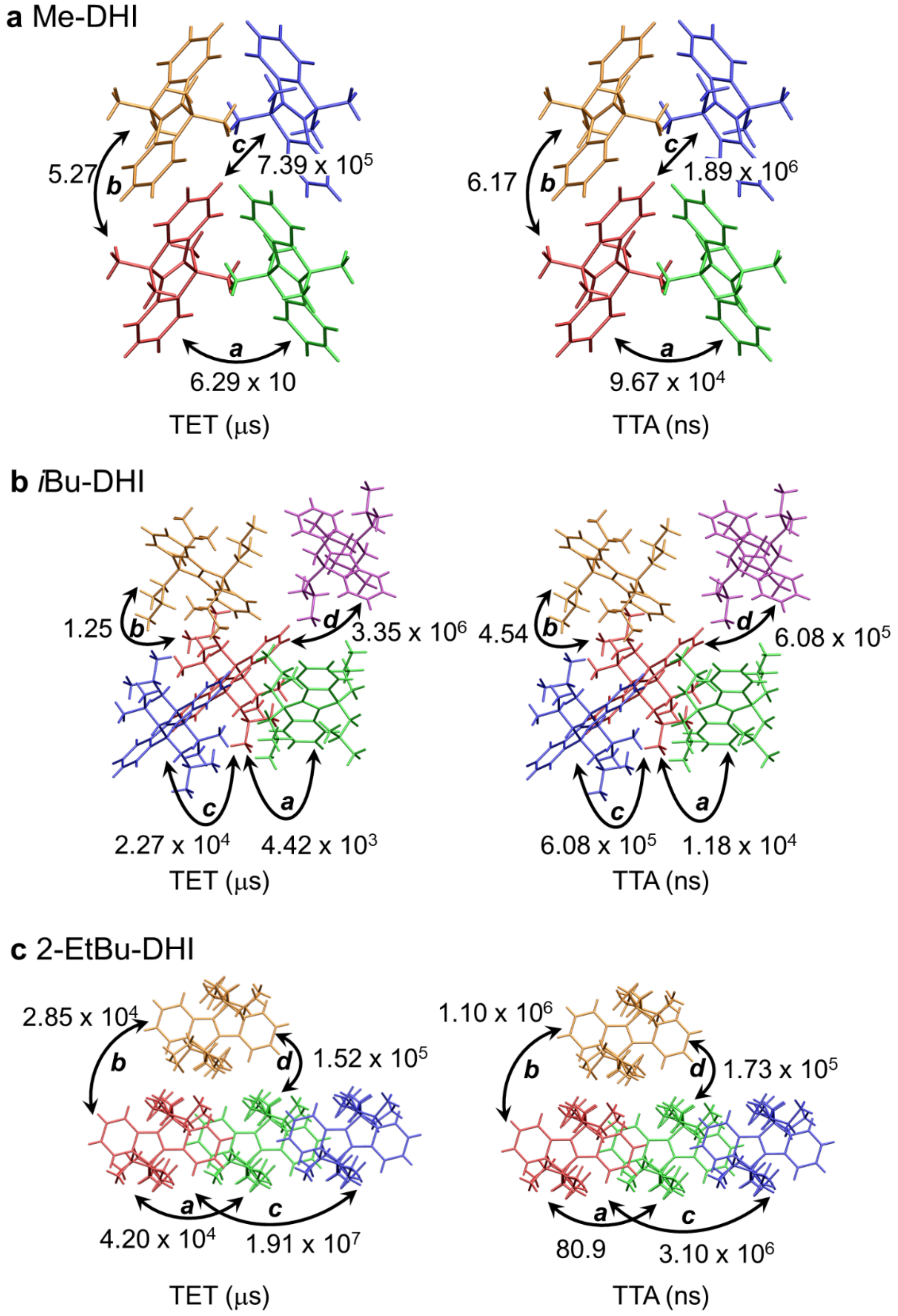

Figure R16. Calculated reaction time of TTA (τ_{TTA}) and TET (τ_{TET}) for **a** Me-DHI, **b** *i*Bu-DHI, and **c** 2-EtBu-DHI.

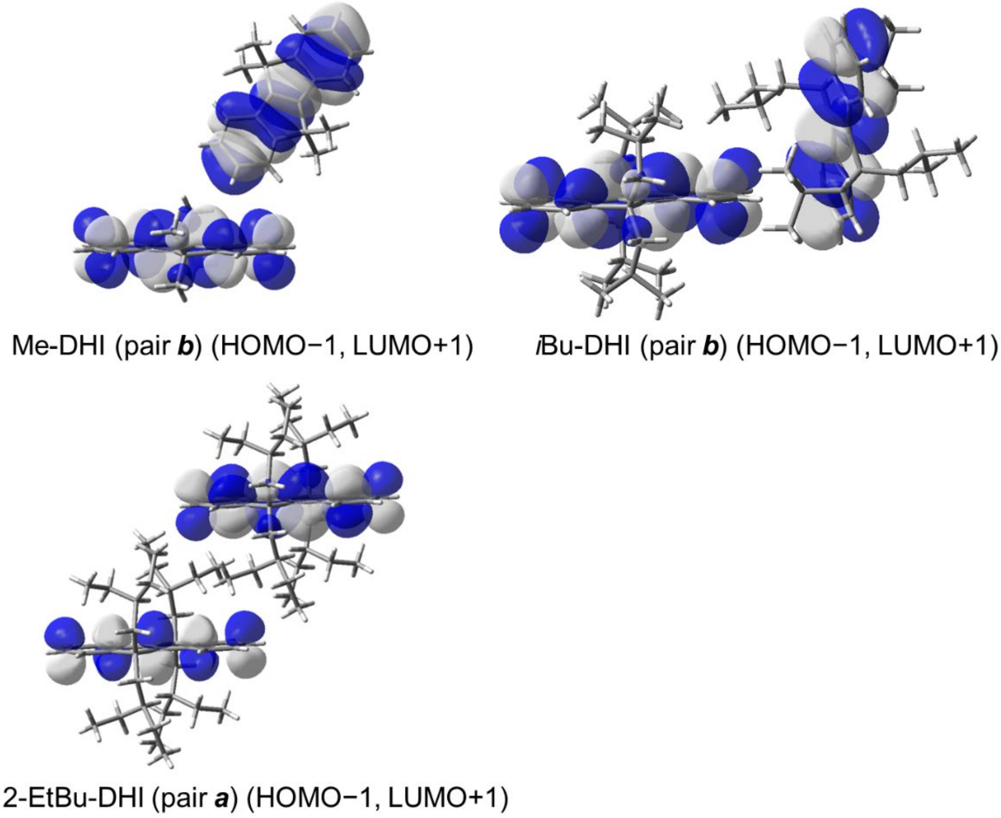

Figure R17. The interaction orbitals of the relevant pairs or pathways, HOMO−1 and LUMO+1, for pairs of Me-DHI (pair ***b***), *i*Bu-DHI (pair ***b***), and 2-EtBu-DHI (pair ***a***) in Figure R16. Iso-surface value is 0.02.

Reviewer 3's comments

In this work, the authors report the fabrication of triplet-triplet annihilation upconversion solid-state devices. The authors use the typical Ir(ppy)3 as sensitizer and present a series of four annihilators: the indenoindene backbone and three derivatives obtained by introducing alkyl spacers at the two sp3 carbons. The authors characterize TTA-UC in solution and solid-state by means of optical measurements to find that in both cases, iBu-DHI is the upconversion efficiency champion, which the authors relate to its higher PLQY, longer triplet lifetime and faster triplet diffusion. Compositional homogeneity of the films is probed using SEM-EDX, which confirms no sensitizer segregation and X-ray diffraction is employed to evaluate crystallinity, with comparison to single crystal structures. Steady-state and time-resolved photophysical measurements are used to determine PLQY, threshold intensity, and excited-state lifetimes. A strong upconversion efficiency from visible light to UV is achieved under excitation intensities below one sun. Additionally, the authors find that the more compact crystalline structure of iBu-DHI prevents oxygen permeation and enables the measurement of UC emission up to 1 hour in an air environment. Overall, the work is reasonably well executed and important for the field, and publication could be considered after addressing the following points:

We appreciate the reviewer's positive comments.

Major comments:

• The authors attribute the increased UC efficiency to higher PLQY, increased lifetime and fast triplet diffusion. Please make clearer evidence to support faster triplet diffusion.

We thank the reviewer for this comment. We have conducted theoretical calculations of TET and TTA reaction rates for the crystalline Me-DHI, *i*Bu-DHI, and 2-EtBu-DHI following the method reported by Sato and Shigeta et al. (*J. Phys. Chem. Lett.* **2018**, *9*, 6638–6643), Figure R14, R15, R16, 4, S28, S29, Table R7, S7). Thanks to sufficient π-orbital overlap between the edges of the π-planes, moderate electronic coupling is realized for *i*Bu-DHI, providing about 4 orders of magnitude faster TET reaction time (1.25 μs) than 2-EtBu-DHI (4.20×10^4 μs for pair ***a***, 2.85×10^4 for pair ***b*,** Figure R16). The TTA reaction times in the same directions were calculated to be 4.54 ns and 8.09×10^1 ns (for pair ***a***) or 1.10×10^6 (for pair ***b***), respectively (Table R7, S7). While the current results cannot rule out the potential effect of the nanoscale donor–acceptor interface structures that we could not clarify due to experimental limitations, the calculated values explain the three orders of magnitude difference in I_{th} between *i*Bu-DHI and 2-EtBu-DHI as it is inversely proportional to the rate constants for TTA. The paragraph was revised as follows, and the details of the calculations were inserted in the supporting information.

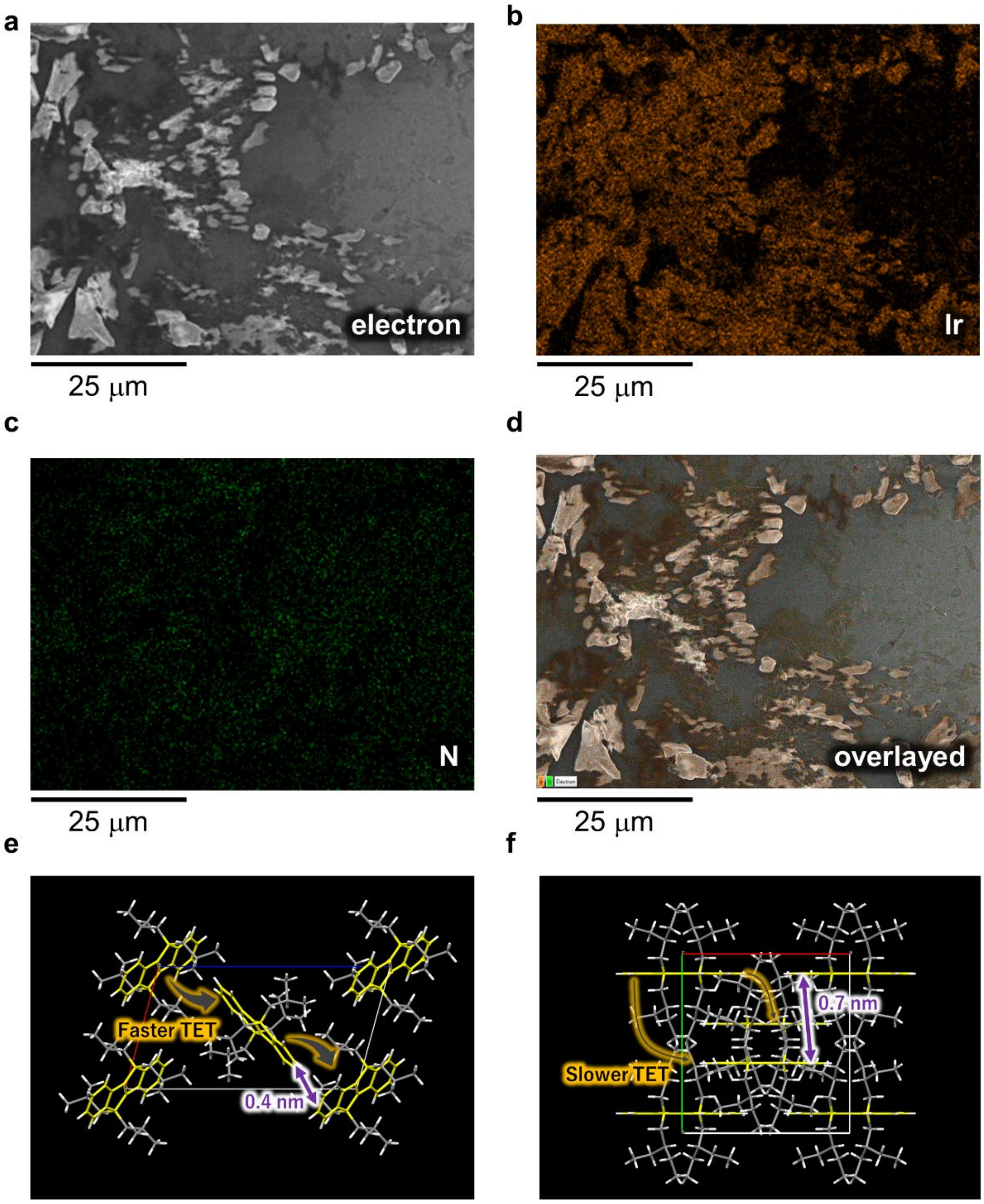

Figure R15. **a** SEM image, and SEM-EDX mappings of **b** Ir, **c** N, and **d** their overlay for the drop-cast film of *i*Bu-DHI with Ir(ppy)$_3$. Molecular packing structures in the crystal of **e** *i*Bu-DHI and **f** 2-EtBu-DHI. The purple double arrows indicate the distances of the closest carbon atoms of the adjacent π-planes. The gray arrows indicating the preferred direction of the triplet diffusion theoretically predicted by *ab initio* calculations are also shown (Table R7).

Table R7. Electronic coupling matrix elements ($|T_{DA}|$) and reaction times (τ) of TET and TTA for Me-DHI, *i*Bu-DHI, and 2-EtBu-DHI.

Molecules	pair	$\left\|T_{DA}^{TET}\right\|$(meV)	τ_{TET}(ms)	$\left\|T_{DA}^{TTA}\right\|$ (meV)	τ_{TTA}(ns)	d [a] (Å)
Me-DHI	a	0.0242	6.29×10^1	0.0119	9.67×10^4	4.444
	b	0.0836	5.27	1.4892	6.17	3.830
	c	0.0002	7.39×10^5	0.0027	1.89×10^6	4.304
*i*Bu-DHI	a	0.0026	4.42×10^3	0.0236	1.18×10^4	5.799
	b	0.1570	1.25	1.2038	4.54	3.756
	c	0.0012	2.27×10^4	0.0099	6.73×10^4	6.208
	d	0.0001	3.35×10^6	0.0033	6.08×10^5	7.313
2-EtBu-DHI	a	0.0009	4.20×10^4	0.2802	8.09×10^1	7.043
	b	0.0011	2.85×10^4	0.0024	1.10×10^6	6.800
	c	0.00004	1.91×10^7	0.0015	3.01×10^6	5.093
	d	0.0005	1.52×10^5	0.0061	1.73×10^5	6.014

[a] Nearest C–C distance between pair of π-conjugation of pair molecules.

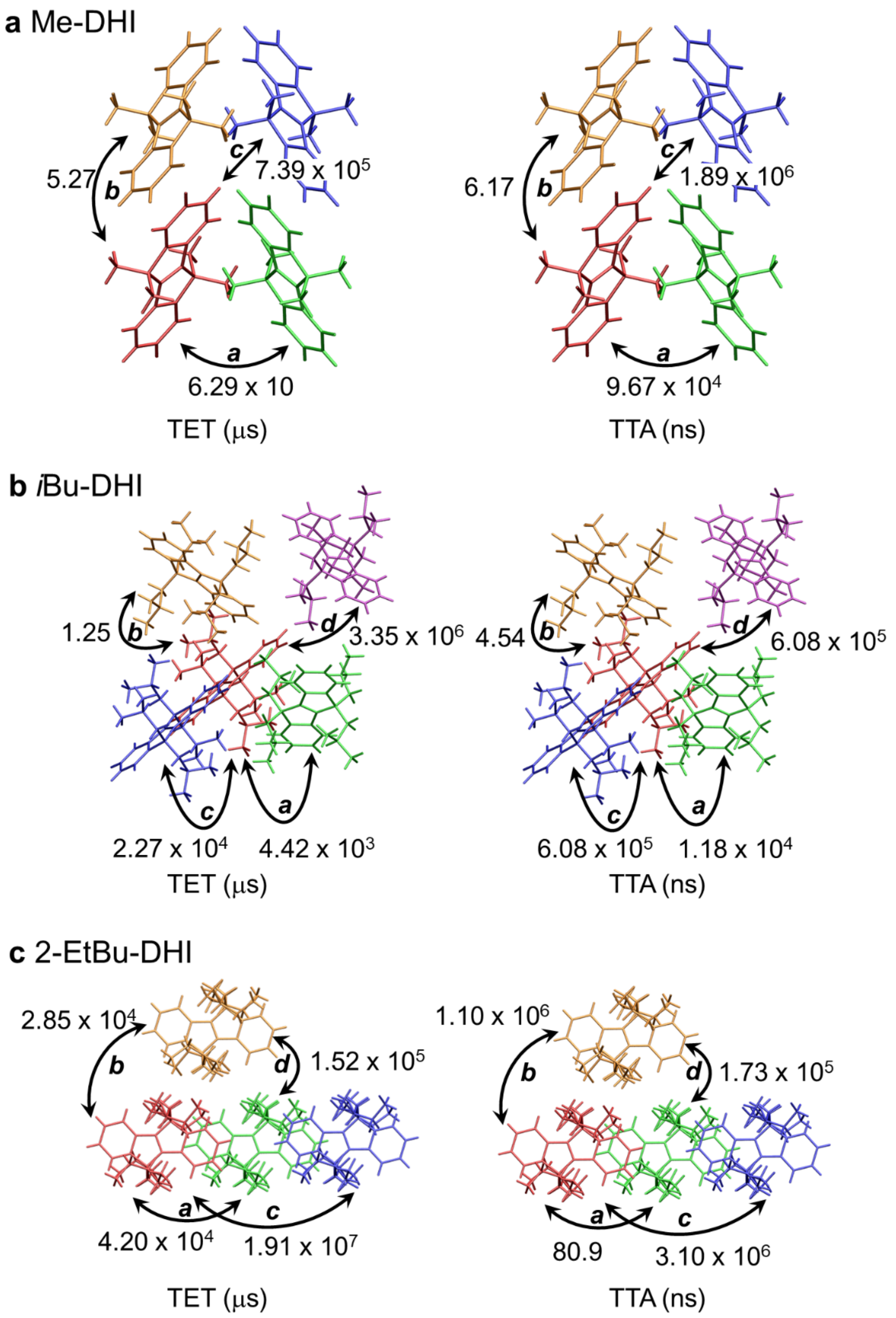

Figure R16. Calculated reaction time of TTA (τ_{TTA}) and TET (τ_{TET}) for **a** Me-DHI, **b** *i*Bu-DHI, and **c** 2-EtBu-DHI.

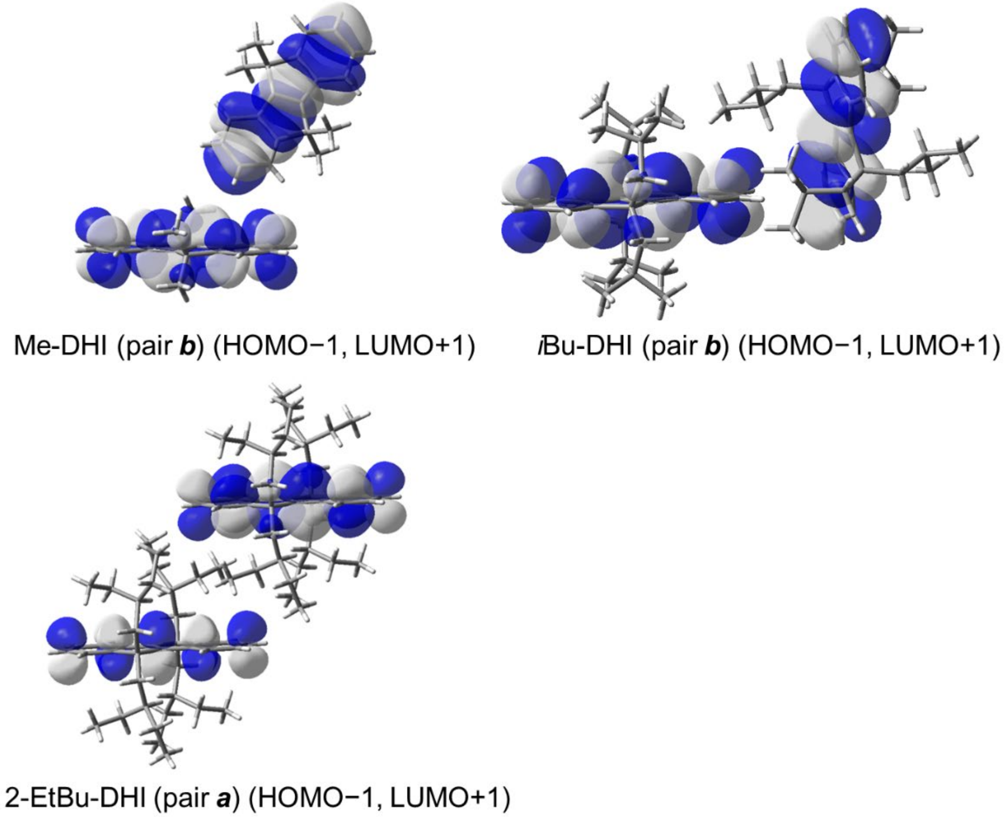

Figure R17. The interaction orbitals of the relevant pairs or pathways, HOMO−1 and LUMO+1, for pairs of Me-DHI (pair ***b***), *i*Bu-DHI (pair ***b***), and 2-EtBu-DHI (pair ***a***) in Figure R16. Iso-surface value is 0.02.

•The authors claim that these devices are unaffected by the formation of crystalline domains. However, without a direct comparison to an amorphous analogue or analysis of morphology–performance correlations, it is difficult to substantiate this claim. Additional evidence is necessary.

We appreciate this suggestion. Due to the highly symmetric structure and limited degree of structural freedom, it was difficult for us to prepare and evaluate amorphous analogues. Instead, we have annealed the spin-coated film of *i*Bu-DHI and $Ir(ppy)_3$ above its melting point (T_m~110 °C) to investigate how changes in the hybrid structure affect the UC properties. A significant drop in UC performance was observed (Figure R18, S15) for the annealed film, and we were unable to evaluate the excitation threshold due to the significantly weak UC emission. This is attributed to phase separation between *i*Bu-DHI and $Ir(ppy)_3$, which reduces TET efficiency, as evidenced by the annealed film showing $Ir(ppy)_3$ phosphorescence in the microscope image (Figure R19f, S16). We changed the commented sentence to the revised manuscript (page 2, line 3).

"Remarkably, the solid-state UC system is less affected by crystalline defects, exhibiting high photoluminescence quantum yield, long triplet lifetime, and fast triplet diffusion, with an absolute Φ_{UC} of 1.9% and a remarkably low excitation threshold intensity of 1.2 mW cm^{-2}."

We also added this data to the revised supporting information (Figure S15) and the following comments to the manuscript (page 9, line 19).

"We also found that when the spin-coated film of *i*Bu-DHI/$Ir(ppy)_3$ was annealed at 120 °C above the melting point of *i*Bu-DHI (T_m ~ 110 ℃), phosphorescence from $Ir(ppy)_3$ was clearly observed from the microscope image (Figure S15, S16f), and UC emission became significantly lower, indicating the reduction of triplet sensitization efficiency due to the segregation. This also underscores the importance of kinetically controlling the sensitizer-doping process in this hybrid material. It is noteworthy that *i*Bu-DHI molecules achieve high donor–acceptor compatibility and excellent TTA-UC properties through simple film-forming techniques, such as room-temperature casting and spin coating, without requiring any special heating treatment."

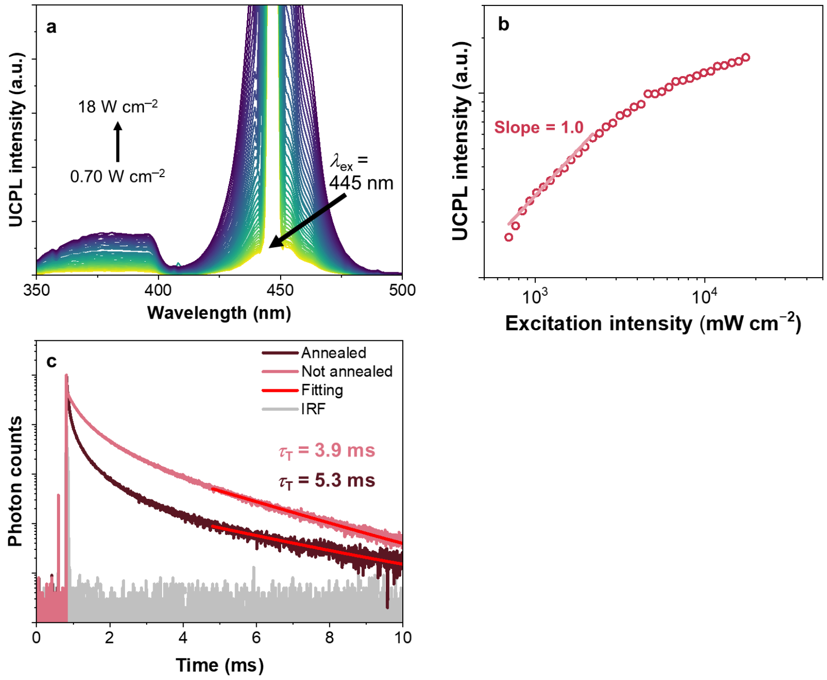

Figure R18. **a** UC emission spectrum and **b** excitation intensity dependence of the upconverted emission intensity of the spin-coated film of *i*Bu-DHI, (T_m ~ 110 °C) doped with $Ir(ppy)_3$ after annealing at 120 °C. Excitation light was removed with a 400 nm short-pass filter. **c** UC emission decay curves of the *i*Bu-DHI UC film before and after annealing.

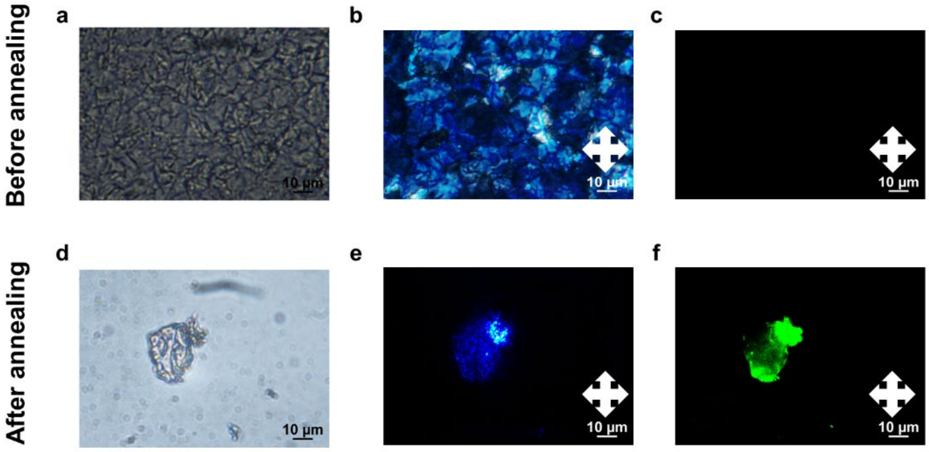

Figure R19. Microscope images of the spin-coated film of *i*Bu-DHI (T_m ~ 110 °C) doped with $Ir(ppy)_3$ **a–c** before and **d–f** after annealing at 120 °C. Images were taken without (**a,d**) and with (**b,e**) crossed nicoles. The cross-polarized images under broad band excitation (450–490 nm) with 505 nm long pass filters to detect $Ir(ppy)_3$ phosphorescence.

• Page 7, Figure S9: the authors wrote "aggregation of 2-EtBu-DHI was observed upon 445nm laser excitation, which might be the reason for negligible UC emission". Could the authors comment on why the compound with the longer alkyl chains is the one susceptible to aggregation?

We appreciate this comment. The aggregation appears to result from the interaction between $Ir(ppy)_3$ and 2-EtBu-DHI as we did not observe aggregates under UV irradiation of the concentrated 2-EtBu-DHI solution. To investigate the possibility of aggregation, we conducted ^{1}H-NMR spectroscopy on mixtures of 2-EtBu-DHI and $Ir(ppy)_3$ at molar ratios of 10:1, 20:1, and 100:1 (Figure R11). The 20:1 and 100:1 conditions are identical to those used for the UC measurements in the Supporting Information. The ^{1}H NMR spectra of all samples showed no signal distortion or chemical shift changes, suggesting that aggregation-induced species are not present in the ground state. Since we observed the formation of a white precipitate in the cuvette after laser exposure (Figure R20), we hypothesize that the aggregation is a photo-induced event that occurs exclusively during laser excitation. Still, we could not pin down the mechanism at this stage. We plan to examine the origin of this phenomenon in a future report.

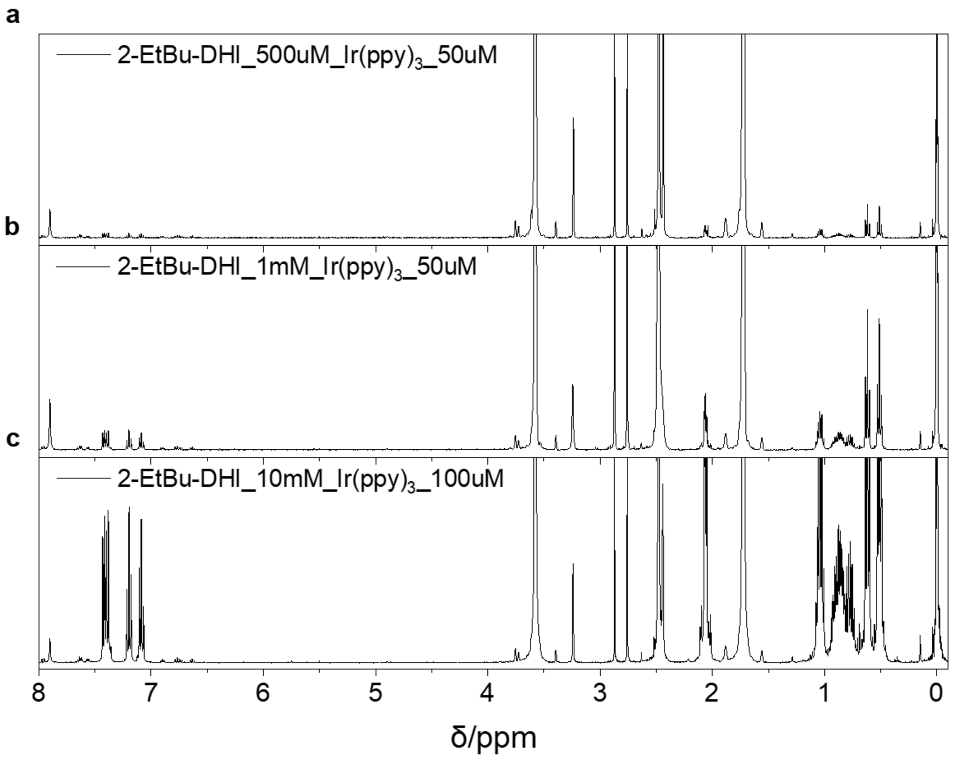

Figure R11. ^{1}H-NMR (400 MHz, THF-*D8*, TMS) spectra of the **a** 500 µM: 50 µM mixture, **b** 1 mM: 50 µM mixture, and **c** 10 mM: 100 µM mixture of 2-EtBu-DHI and $Ir(ppy)_3$.

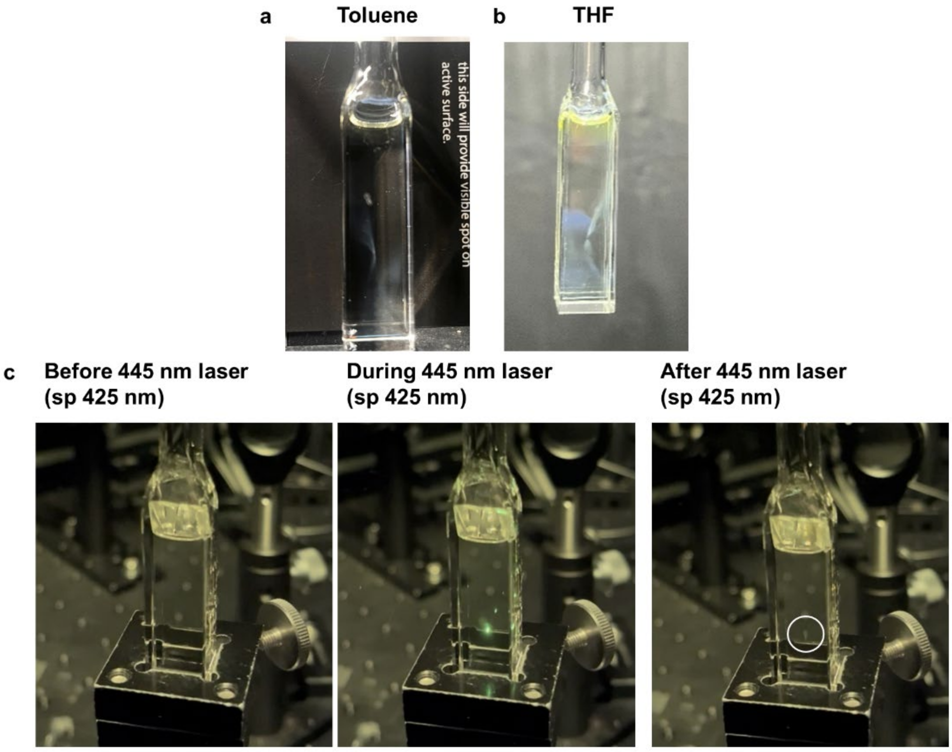

Figure R20. Photographs illustrating the influence of solvent environment on the formation of molecular aggregates in **a** toluene and **b** THF. **c** Solvent-dependent aggregation behavior and photo-induced assembly of 2-EtBu-DHI with Ir(ppy)$_3$.

Was the compound stable in solution upon excitation for other measurements (e.g. quantum yield, fluorescence lifetime)?

In the absence of Ir(ppy)$_3$, we did not find precipitates. Therefore, we could measure the quantum yield and fluorescence lifetime of 2-EtBu-DHI. However, during the UC lifetime measurements using the 445 nm xenon flash lamp, precipitation was also observed.

The compound can be dissolved in other solvents (i.e. methylene chloride is used for characterization by NMR spectroscopy); does this aggregation occur also in other solvents?

The compound dissolved in THF, toluene, chloroform, and dichloromethane. The compound slightly dissolved in cyclohexane, hexane, and dimethyl formaldehyde. To investigate whether aggregation occurs in other solvents, we tested the samples in toluene and THF (Figure R20). In these two solvents, aggregations were observed, suggesting that the interaction between Ir(ppy)$_3$ and 2-EtBu-DHI is not limited to a specific solvent environment. This indicates that the aggregation behavior is likely driven by intrinsic molecular interactions rather than solvent-specific effects.

Can the authors comment on the possibility of this aggregation being the reason why solid-state devices with 2-EtBu-DHI as an acceptor have the lowest performance?

We appreciate this critical comment. The PLQY of 2-EtBu-DHI is poorer than that of others, which is possibly due to the aggregation. As for the threshold excitation intensity, we observed a moderately high fluorescence quantum yield and a long upconversion emission lifetime similar to those of *i*Bu-DHI, indicating that quenching is not significant in crystalline 2-EtBu-DHI. Together with the theoretical calculations of TET and TTA rates, we think the poorer UC properties in the solid 2-Et-Bu-DHI with $Ir(ppy)_3$ can be mainly explained by the calculated TET and TTA rates, which provided orders-of-magnitude differences in the TTA and TET rates.

• In page 9, the authors comment on the similarities/differences in XRD diffraction patterns between single crystals and deposited samples. Given the difference in the position of the main reflection between drop casted and spin coated samples, could the authors comment on what does this imply (i.e. preferred crystalline orientation) and the effect it might have on TTA-UC?

We appreciate this comment. We found that spectral shifts and linewidths were caused by differences in substrate position and slit widths. We retook the measurements with proper positioning and the same slit. This produced similar peak patterns and line widths. Figure 3f was replaced with new data, and the description on spectral broadening was removed accordingly.

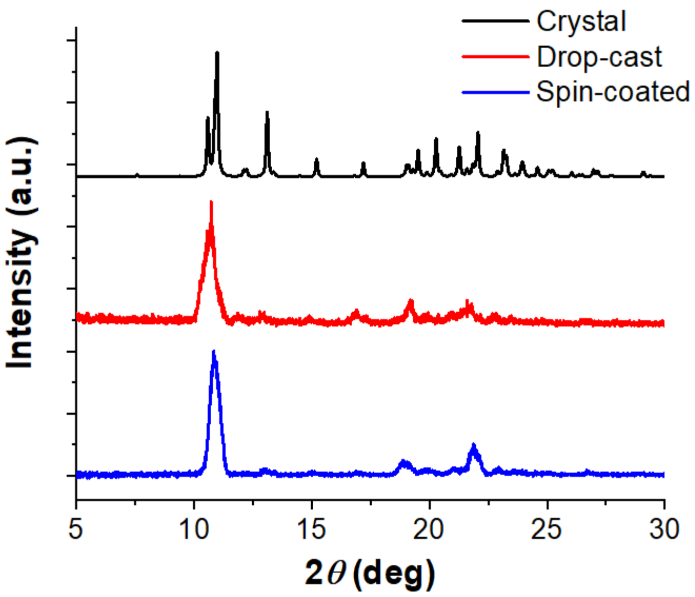

Figure R21. Comparison of XRD patterns of crystalline solid of *i*Bu-DHI (black) and solid samples of *i*Bu-DHI with $Ir(ppy)_3$ prepared by spin-coating (blue) and drop-casting (red) methods.

• Page S23, Figure S19. In panel a, add legend/arrow/color code indicating the changes in spectra upon time.

We appreciate this comment. We have modified the graph as shown in Figure R13 and inserted it in the revised manuscript (Figure S23).

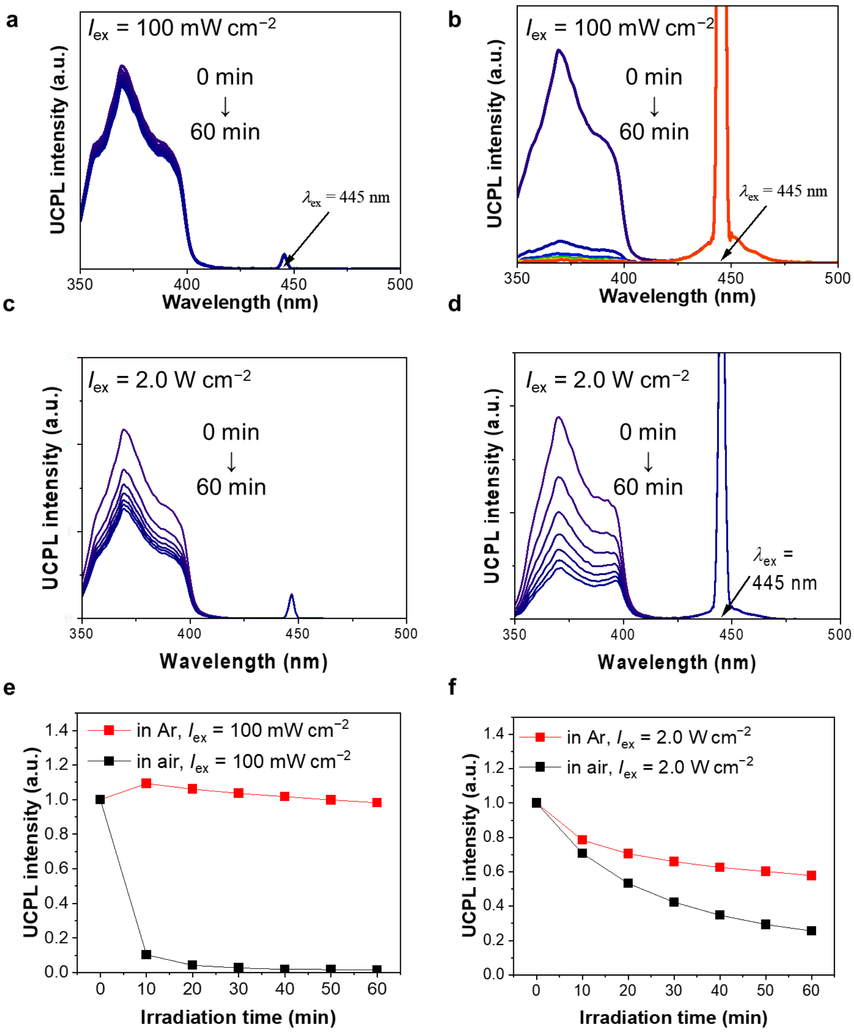

Figure R13. Time-dependent UC emission spectra of a spin-coated film of *i*Bu-DHI/Ir(ppy)$_3$ (molar ratio for preparation; *i*Bu-DHI : Ir(ppy)$_3$ = 100 : 1) prepared and measured **a** in Ar and **b** in air were recorded under excitation at 100 mW cm^{-2}. **c** The results in Ar and **d** in air were recorded under 2.0 W cm^{-2}. Excitation light was removed with a 400 nm short-pass filter. Time-dependent UC emission intensity with (red) and without (black) encapsulation **e** under excitation intensity at 100 mW cm^{-2} and **f** at 2.0 W cm^{-2} (λ_{dt} = 370 nm). It was difficult to evaluate UCQY and I_{th} in air at t = 0 min under air-saturated conditions because the weak UCPL intensity required approximately 1 minute of exposure time.

The caption of figure S19a states that the UC emission of the spin-coated film was "prepared and measured in air"; contrarily, in the main script (page 11) the text states "prepared in air-saturated conditions showed UC emission without encapsulation in an inert atmosphere". These statements seem contradictory. Could the authors clarify?

We apologize for the confusion. Both descriptions indicate that the samples were prepared in air and subsequently measured under ambient conditions without encapsulation. We have corrected the descriptions as follows (page 11, line 24),

"The spin-coated film of *i*Bu-DHI/Ir(ppy)$_3$ prepared in air-saturated conditions showed UC emission in air"

In some solid-state devices, it has been observed that higher power density excitation can lead to sample degradation, even under inert conditions. Could the authors comment on why they chose to use different power densities (up to 3 orders of magnitude). This experiment should be repeated comparing both samples at the same power density excitation (presumbly the highest one, which is needed to activate TTA-UC in the presence of oxygen).

We appreciate the comment. We have measured the stability of UC emission at an excitation intensity that provides clear UC spectra. For the fair comparison, we have measured the intensity again at the same excitation intensity of 100 mW cm^{-2} and 2.0 W cm^{-2} (Figure R13). While the UC emission intensity remained stable at 100 mW cm^{-2} in Ar, it significantly dropped in air. At a high excitation intensity of 2.0 W cm^{-2}, the UC emission intensity decreases even in Ar, possibly due to sample melting or damage. We have inserted the following data in the revised manuscript.

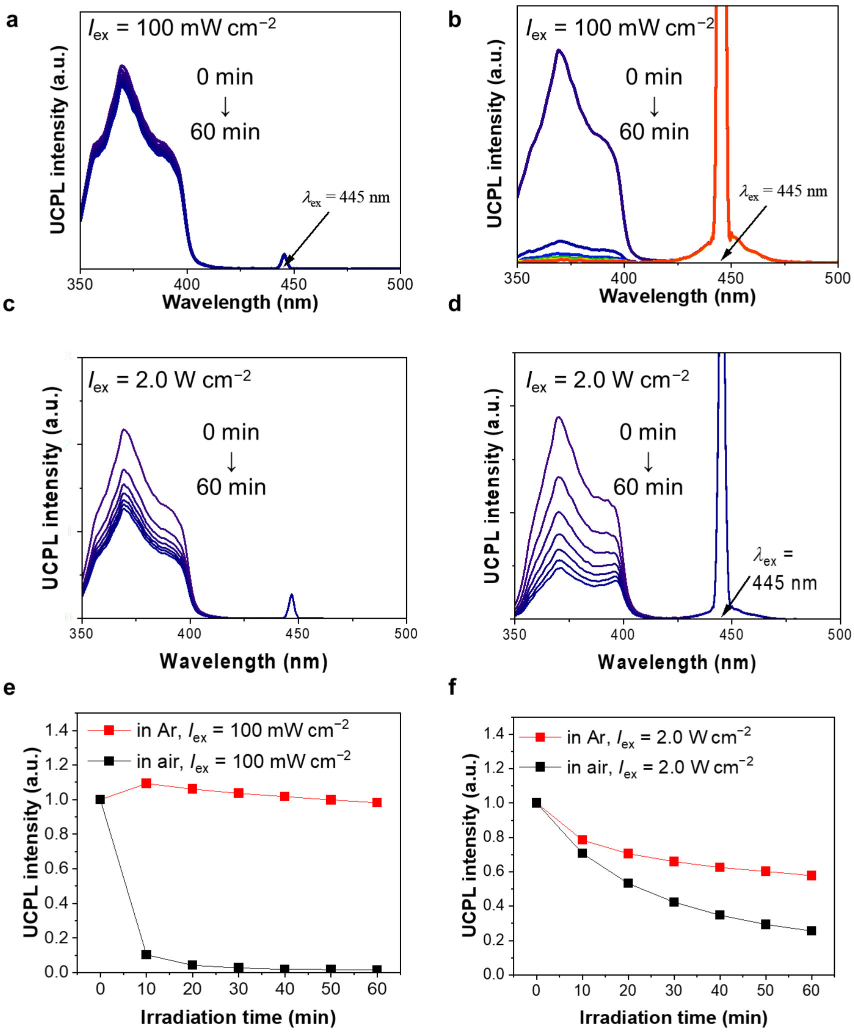

Figure R13. Time-dependent UC emission spectra of a spin-coated film of *i*Bu-DHI/Ir(ppy)$_3$ (molar ratio for preparation; *i*Bu-DHI : Ir(ppy)$_3$ = 100 : 1) prepared and measured **a** in Ar and **b** in air were recorded under excitation at 100 mW cm^{-2}. **c** The results in Ar and **d** in air were recorded under 2.0 W cm^{-2}. Excitation light was removed with a 400 nm short-pass filter. Time-dependent UC emission intensity with (red) and without (black) encapsulation **e** under excitation intensity at 100 mW cm^{-2} and **f** at 2.0 W cm^{-2} (λ_{dt} = 370 nm). It was difficult to evaluate UCQY and I_{th} in air at t = 0 min under air-saturated conditions because the weak UCPL intensity required approximately 1 minute of exposure time.

The authors state that the high-density molecular assembly of iBu-DHI/Ir(ppy)3 prevents oxygen diffusion. Would the 2-EtBu-DHI films be considered lower-density packaging, given that the distance between DHI units is larger for this compound? If so, could they compare the stability against oxygen for these two films?

We appreciate this comment. As mentioned by the referee, the crystal density of DHI derivatives decreases as the side group becomes bulkier. Since the TTA-UC emission depends on oxygen quenching and triplet diffusion, the triplet lifetime, it is tough to extract the contribution of the solid density alone.

In my opinion, Figure 4b is not sufficient to make claims about Irppy dispersion, as the scale is dramatically too big to draw conclusions.

We appreciate the comments. As the resolution of the SEM-EDX measurement resolution is on the micrometer scale, we cannot rule out the possibility of nano-scale aggregations. In addition, increasing the acceleration voltage above ~15 kV caused significant damage and melted the sample whereas zooming at 5 kV yielded a very opaque image, limiting resolution. Therefore, we just mentioned the "micrometer-scale segregation". We have added the following sentence (page 11, line 6).

"Ir and N atoms of Ir(ppy)$_3$ are homogeneously distributed in these microcrystals without micrometer-scale segregation, unlike the commonly observed inhomogeneous solid-state sensitizer–annihilator mixtures."

Minor comments:

• Please avoid claims like "never been developed"

We appreciate this comment. We have revised the manuscript by deleting the phrase "never been developed" and modifying the sentence as shown below (page 1, line 19),

"Realizing such a solid-state TTA-UC system has been challenging because it is difficult to achieve high fluorescence quantum yield and fast triplet exciton diffusion simultaneously, which requires methodologies to precisely control chromophore interactions and suppress quenching of both singlet and triplet excited states. "

• The field has moved away from the "free factor of 2" in upconversion efficiencies. Please consider removing from your calculations.

We appreciate this suggestion. We have removed "free factor of 2" from upconversion efficiencies and replaced all UC efficiency with "UC quantum yield (Φ_{UC})" in the revised manuscript.

(page 18, line 12) $\Phi_{\text{UC}} = \Phi_{\text{Std}}\left(\frac{1-10^{-A_{\text{Std}}}}{1-10^{-A_{\text{UC}}}}\right)\left(\frac{E_{\text{UC}}}{E_{\text{Std}}}\right)\left(\frac{I_{\text{Std}}}{I_{\text{UC}}}\right)\left(\frac{n_{\text{UC}}}{n_{\text{Std}}}\right)^2$

(page S15, line 11) $\Phi_{\text{UC}} = \frac{1}{2}\Phi_{\text{ISC}}\Phi_{\text{TET}}\Phi_{\text{TTA}}\Phi_{\text{F}}f$

(page S22, line 10) $\Phi_{\text{UC,obs}} = \Phi_{\text{UC}}(1-a)$

• In figure 1b, the authors should describe the chemical nature of the sensitizer.

We appreciate the comments. We have inserted the structure of Ir(ppy)3 as the "Donor" to Figure 1b as shown below (Figure R22b).

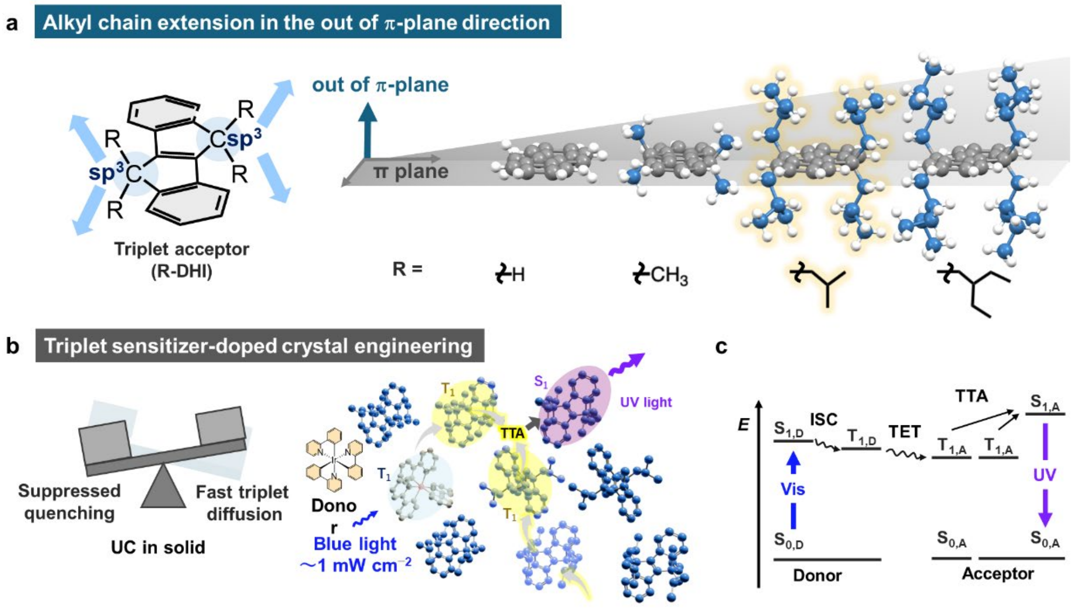

Figure R22. **a** Controlled intermolecular interaction by extending alkyl chains from the sp^3 carbon of dihydroindeno[2,1-a]indene (DHI) derivatives in the out-of-π-plane direction. **b** Crystal engineering for highly efficient solid TTA-UC systems that simultaneously achieve suppression of the excited singlet and triplet quenching, fast triplet diffusion, and donor solubilization. **c** An energy diagram of the TTA-UC process from visible to ultraviolet light.

• In figure 2, the ground and solution fluorescent lifetimes are hard to distinguish, having the same marker and a very similar color. Authors should change this for greater clarity. Arrows to indicate which axis is related to markers and bars should be added.

We appreciate the comment. We have modified the graph shown in Figure R23 to make the color difference more straightforward in the revised manuscript.

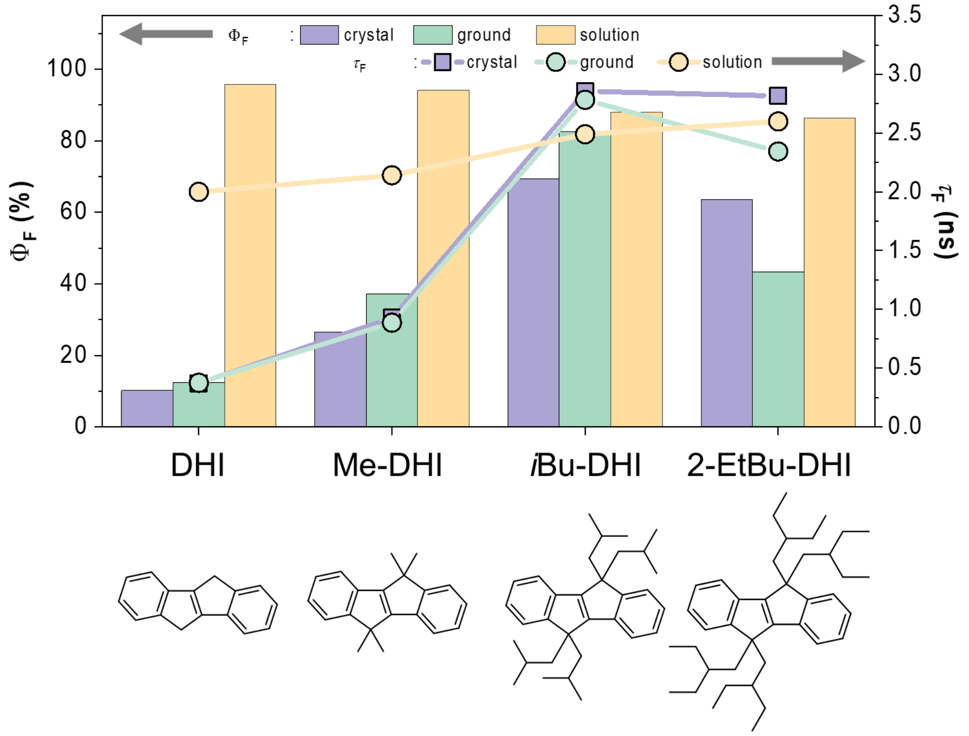

Figure R23. Fluorescence quantum yields (Φ_F) and fluorescence lifetimes (τ_F) of DHI, Me-DHI, *i*Bu-DHI, and 2-EtBu-DHI in the crystalline solid (purple), in the ground powder (green), and in the solution state (yellow; 200 µM in deaerated THF). Chemical structures of DHI, Me-DHI, *i*Bu-DHI, and 2-EtBu-DHI are also shown at the bottom.

• Page 10, Figure 3c: missing a legend (green, blue and red points can be deduced from Figure 3b, not grey data points, which is the same acceptor as red but drop-casted instead of spinned). Additionally, authors should add Ith to all of them.

We appreciate the referee for pointing out this. We have added the legend and inserted "I_{th}" to the graph as shown in Figure 4. We have updated Figure 4 in the revised manuscript.

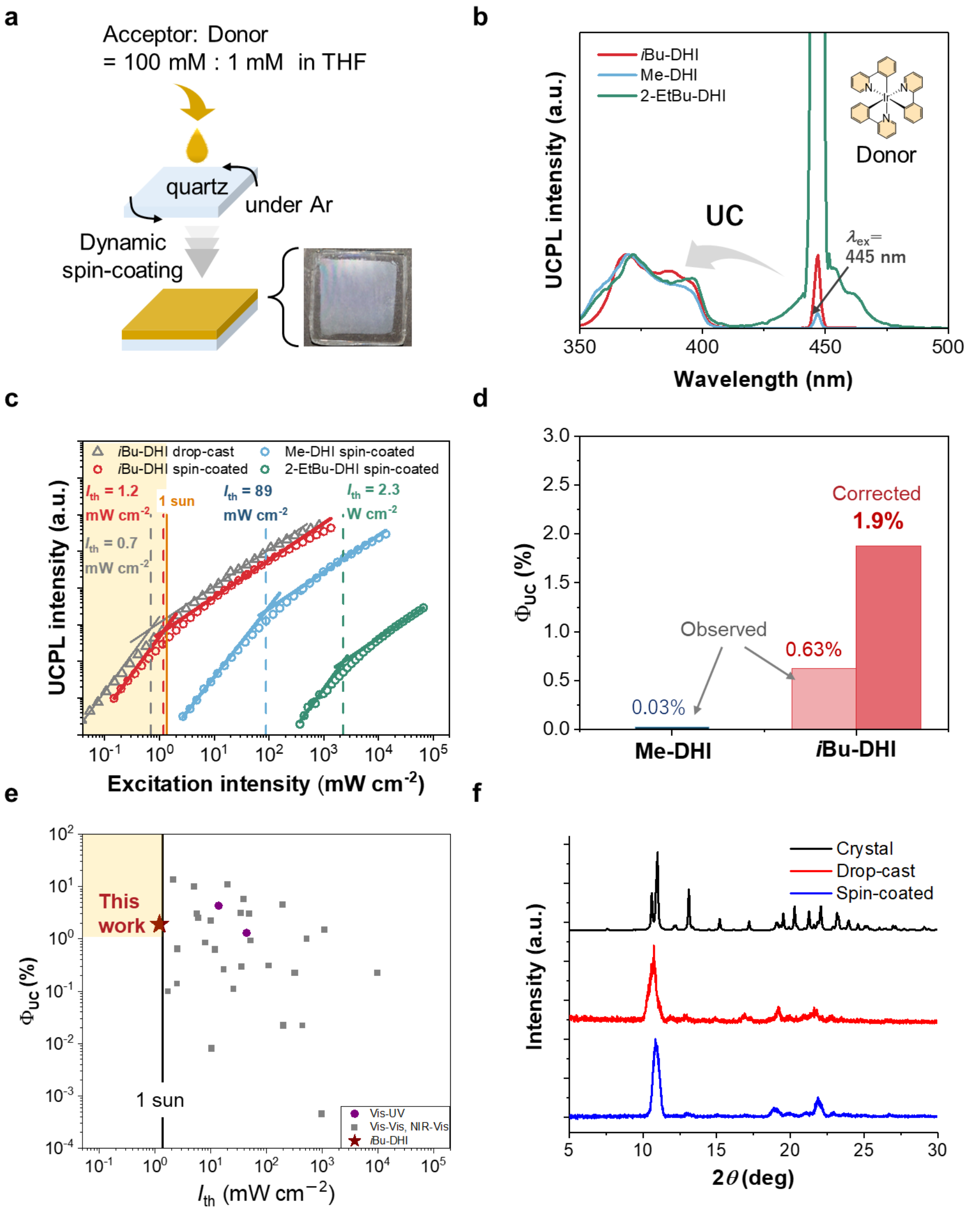

Figure R24. **a** Fabrication procedure of UC solid films by dynamic disperse spin-coating, **b** UC emission spectra, **c** Excitation intensity dependence of the upconverted emission intensity around 370 nm, and **d** absolute Φ_{UC} in solid of Me-DHI (blue), *i*Bu-DHI (red), and 2-EtBu-DHI (green) paired with Ir(ppy)$_3$ (λ_{ex} = 445 nm, short-pass filter: 400 nm, molar ratio for preparation; acceptor: donor = 100: 1). The gray plots in Figure 3c show the data of *i*Bu-DHI/Ir(ppy)$_3$ prepared by the drop-casting method. **e** Φ_{UC}–I_{th} plots with the reported values of solid TTA-UC systems. The details of the references for the plot are shown in the supporting information (Figure S30 and Table S8). **f** Comparison of XRD patterns of crystalline solid of *i*Bu-DHI (black) and solid samples of *i*Bu-DHI with Ir(ppy)$_3$ prepared by spin-coating (blue) and drop-casting (red) methods.

• Page S14, Figure S11. Legend indicates laser used is 369 nm while caption states 445 nm.

We appreciate this comment. In this measurement, the excitation wavelength was 445 nm and the detection wavelength was 369 nm. We have corrected the legend and caption of Figure R25 (S12) to avoid confusion.

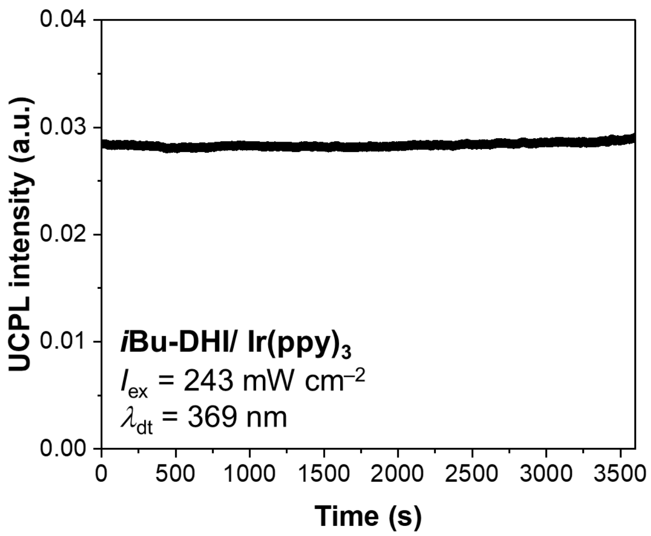

Figure R25. Time-dependent UCPL intensity in deoxidized THF ([*i*Bu-DHI] = 10 mM, [Ir(ppy)$_3$] = 100 µM, in an Ar atmosphere) under continuous laser irradiation for 1 hour (λ_{ex} = 445 nm, λ_{dt} = 369 nm, I_{ex} = 243 mW cm^{-2}).

• The addition of the crystalline structure of DHI would help visualize the pi-stacking that is prevented in the derivatives thanks to the addition of the alkyl chains.

We appreciate the comment. We inserted the crystal structure of DHI in the revised manuscript (Figure R26, S27).

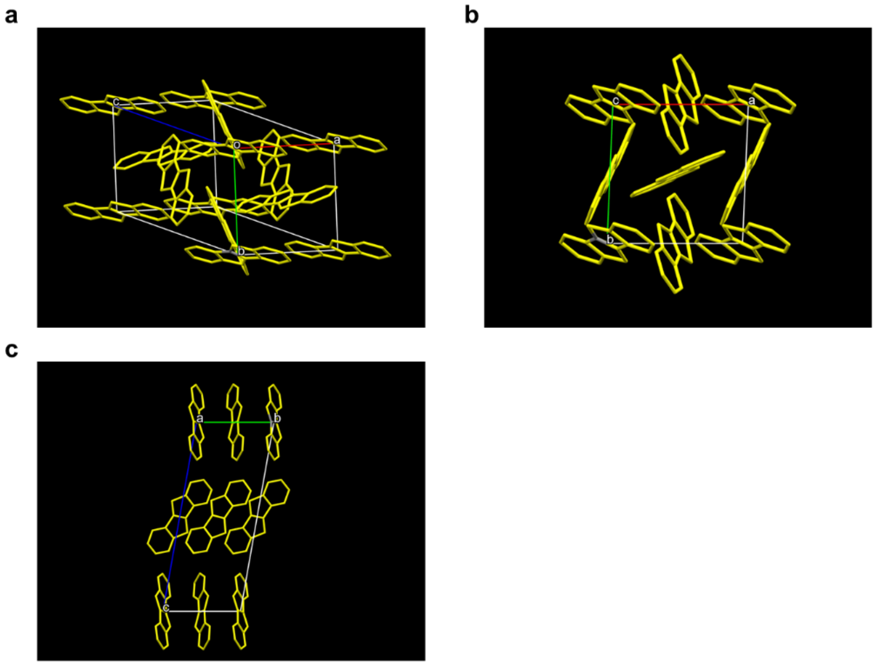

Figure R26. Reported crystal structure of DHI (CSD deposition number: 1949606, DOI:10.5517/ccdc.csd.cc23fqjt). **a** A diagonal view of the unit cell from above, **b** the packing on the ab plane, and **c** on the bc plane.

Response Letter

Comments from Reviewer 1

The authors addressed all of the (mostly minor) issues that I had very carefully.

I also had a look at the comments of the other referees and I believe that the authors did a great job during the revision. The manuscript with the outstanding results on solid-state upconversion systems should be published without further delay as the findings and the high-level methodology will certainly stimulate further research activities.

We appreciate the reviewer's positive comments.

Comments from Reviewer 2

While the revision addresses some points, several issues still remain and require further revision.

We thank the reviewer 2 for valuable suggestions.

1) The authors introduced a ½ prefactor in Eq. 3 (SI), enforcing the 50% maximum for UC QY, but the revised manuscript's relative-method equation for UC QY omits this prefactor and therefore treats UC QY like an ordinary PLQY. As written, that equation can output up to 100% (since PLQY is bounded by 100%), which is inconsistent with their SI definition and leaves the "factor of 2" problem unresolved.

We apologize for the confusion caused by our previous response. As referee 2 mentioned, the equation for the relative UCQY is equivalent to the one widely used for PLQY (a comparison of the ratio of absorbed photons to the emitted photons, Figure R1). The evaluation of relative UCQY uses the sensitizer's absorption, and the UC scheme includes a bimolecular process of triplet-triplet annihilation (TTA), which converts 2 free triplets into 1 triplet pair intermediate (TT) that can produce a singlet. This results in a theoretical maximum of 50% (Figure R1a). This UCQY is generally expressed as the product of efficiency of intersystem crossing (ISC), triplet energy transfer (TET), triplet-triplet annihilation (TTA), singlet generation efficiency (*f*), and fluorescence (Equation R2 in Table R1). Note that we must divide the product by 2 to obtain the UCQY, as the bimolecular TTA process halves the yield. This equation is important when we calculate the *f* value. Many groups multiply the UCQY by 2 to obtain a standardized value (η_{UC}), which has a theoretical maximum of 100% (Equation R3, R4 in Table R1). However, as referees 1 and 3 noted, this expression may lead to confusion in the quantitative evaluation of the UC process. In the revised manuscript, we used the expression without the perfector 2 to achieve a theoretical maximum of 50% (Equations R1 and R2 in Table R1).

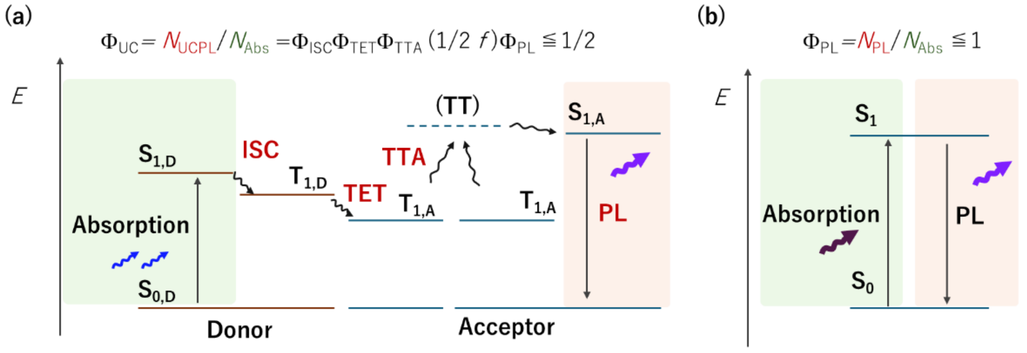

Figure R1. (a) Energy diagram and photophysical processes in TTA-UC and (b) in general fluorescence. Triplet pair intermediate (TT) was included to explain each process (*JACS Au*, **2021**, *1*, 2188–2201).

Table R1. Comparison of the equations for UCQY evaluations in the revised manuscript and the initial manuscript.

	Revised manuscript	Initial manuscript
Equations for the relative method	$\Phi_{UC} = \Phi_{Std}\left(\frac{1\text{-}10^{-A_{Std}}}{1\text{-}10^{-A_{UC}}}\right)\left(\frac{E_{UC}}{E_{Std}}\right)\left(\frac{I_{Std}}{I_{UC}}\right)\left(\frac{n_{UC}}{n_{Std}}\right)^2$ Eq. R1	$\eta_{UC} = 2\Phi_{Std}\left(\frac{1\text{-}10^{-A_{Std}}}{1\text{-}10^{-A_{UC}}}\right)\left(\frac{E_{UC}}{E_{Std}}\right)\left(\frac{I_{Std}}{I_{UC}}\right)\left(\frac{n_{UC}}{n_{Std}}\right)^2$ Eq. R3
Φ_{UC} or η_{UC} expressed by products of each process	$\Phi_{UC}=\frac{1}{2}f\Phi_{ISC}\Phi_{TET}\Phi_{TTA}\Phi_{F}$ Eq. R2	$\eta_{UC}=f\Phi_{ISC}\Phi_{TET}\Phi_{TTA}\Phi_{F}$ Eq. R4
Theoretical maximum	50%	100%

2) The claim "no new 77K emission features ⇒ no ISC-mediated deactivation" (Fig. R4) is not justified, because phosphorescence can be fully quenched nonradiatively or too weak to be observed under these measurement conditions. So, these results cannot exclude ISC as a competing deactivation channel.

We appreciate this comment. As referee 2 pointed out, we cannot exclude ISC as the deactivation channel for PLQY in unsubstituted DHI in crystals. We have added the following comment to the revised manuscript (page 7, line 7).

"Changes in molecular geometry upon crystallization could also promote intersystem crossing to the triplet state and enhance the nonradiative deactivation process."

3) Fig. 3e and Table S8 do not provide a clean "solid-state" benchmark: it mixes genuinely rigid systems with low-T_g matrices (e.g., Ref. 16, 31) where molecular diffusion contributes to TTA-UC, making the comparison misleading. At the same time, authors omit relevant high-performing, truly solid-state reports (e.g., Mater. Horiz., 2022,9, 3048; Sol. RRL 2022, 2100873, Adv. Optical Mater. 2025, 13, 2403032), so the literature context is selective and incomplete.

We appreciate the referee for pointing out this. Following the reviewer's comment, we have updated Table S8 (Table R2, Figure R2-R5), Figure 3e, and Figure S30 (Figure R6, R7) to focus exclusively on solid-state systems, excluding molecular diffusion-based systems.

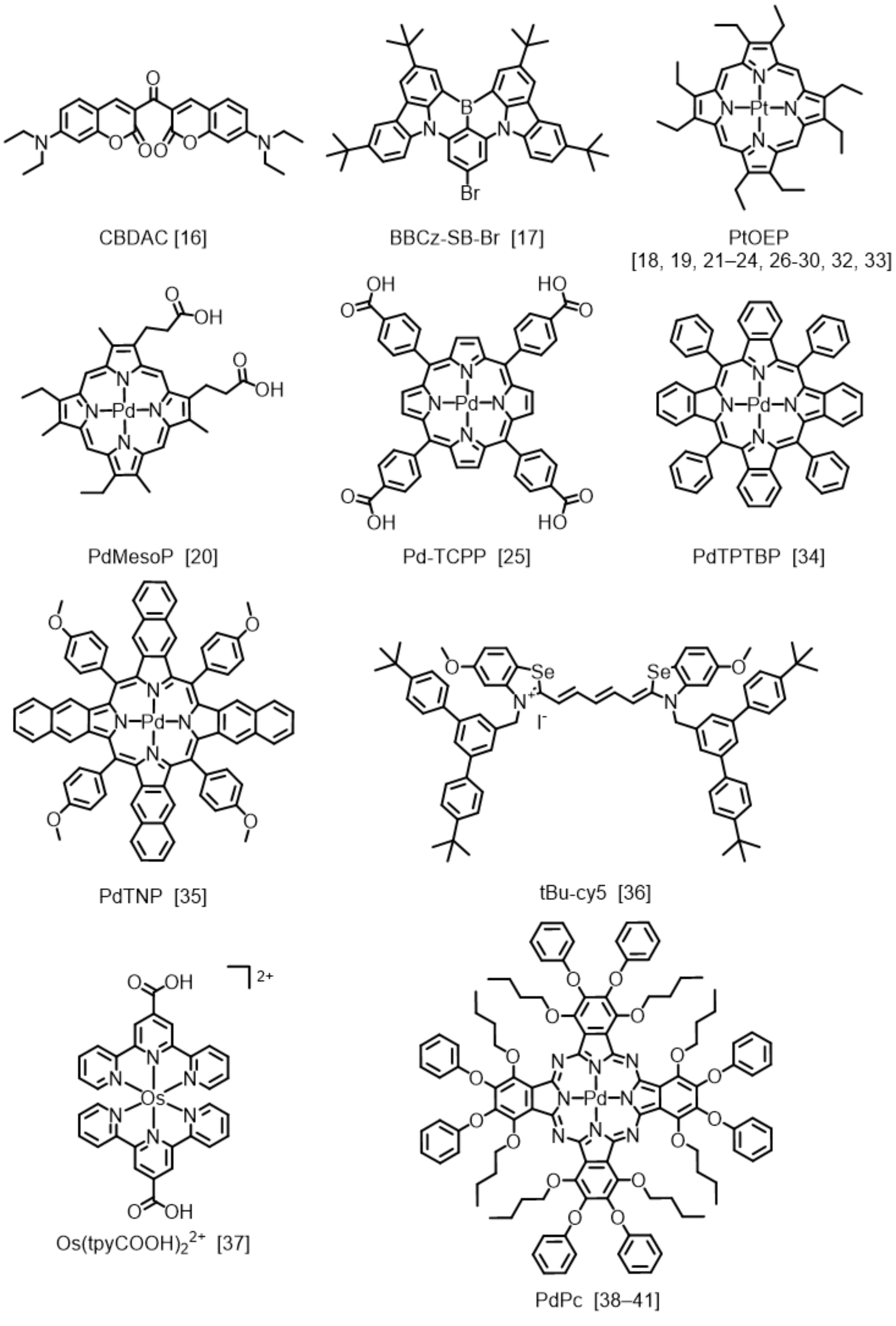

Figure R2. Chemical structures of molecular triplet donors from Ref. 16 to 41.

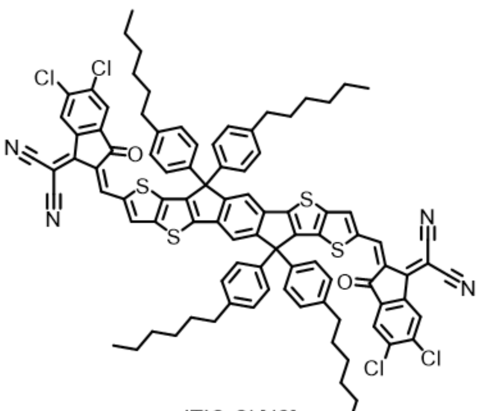

ITIC-Cl [42]

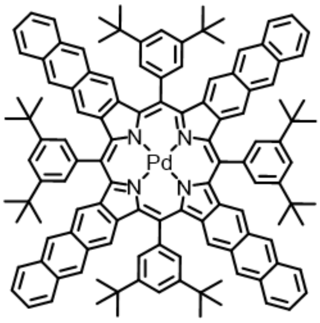

PdTPTAP [44]

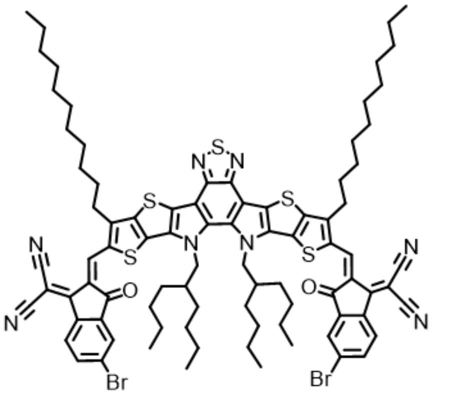

PYIT1 [47]

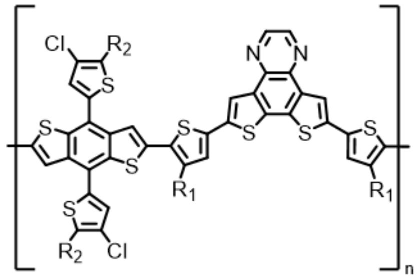

PBQx-TCl [47]

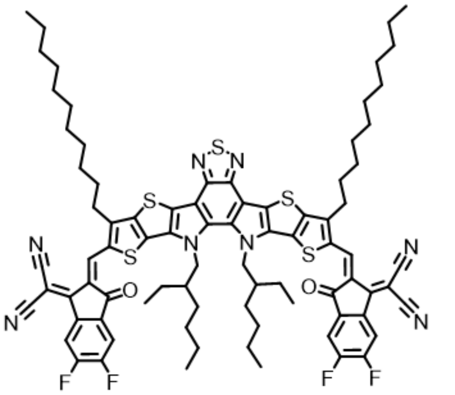

Y6[42, 48]

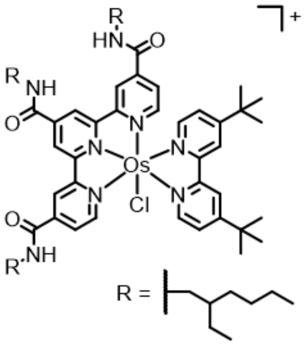

Os(atpy)(tbbpy)Cl^{+} [50]

Figure R3. Chemical structures of molecular triplet donors from Ref. 42 to 50.

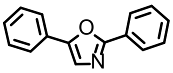

PPO [16]

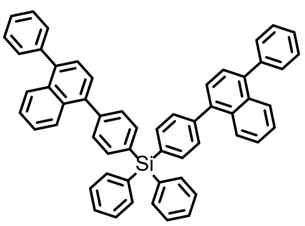

SiDPN-2 [17]

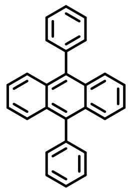

DPA [18, 22, 24, 28, 30]

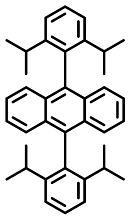

bDPA-2 [19]

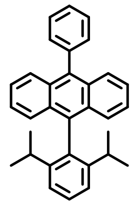

bDPA-1 [19]

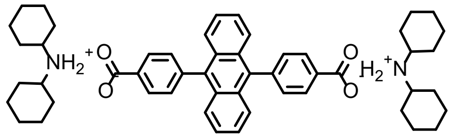

$(DCA)_2ADC$ [20]

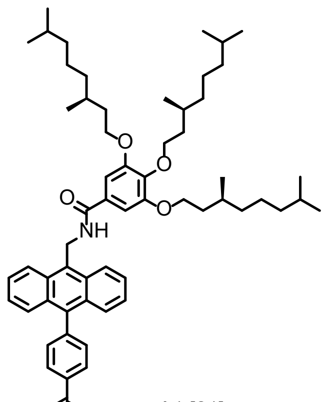

A1 [21]

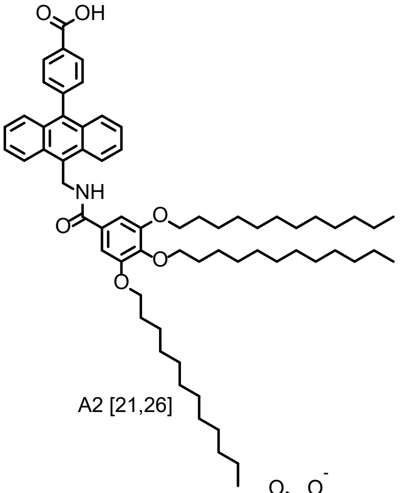

A2 [21,26]

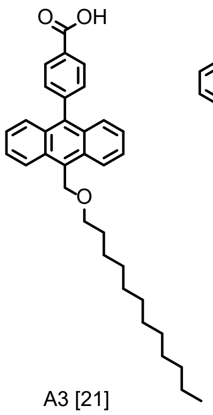

A3 [21]

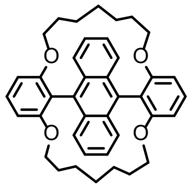

C7-sDPA [23]

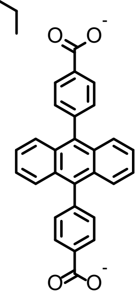

adba [25, 31]

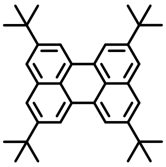

TTBP [26]

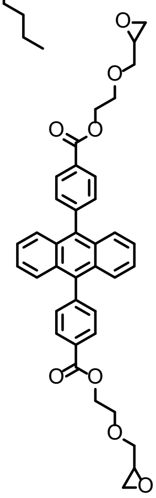

Epo-DPA [27]

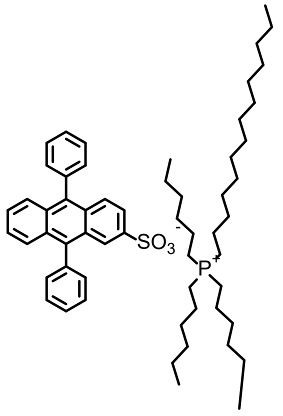

$P_{66614}DPAS$ [27]

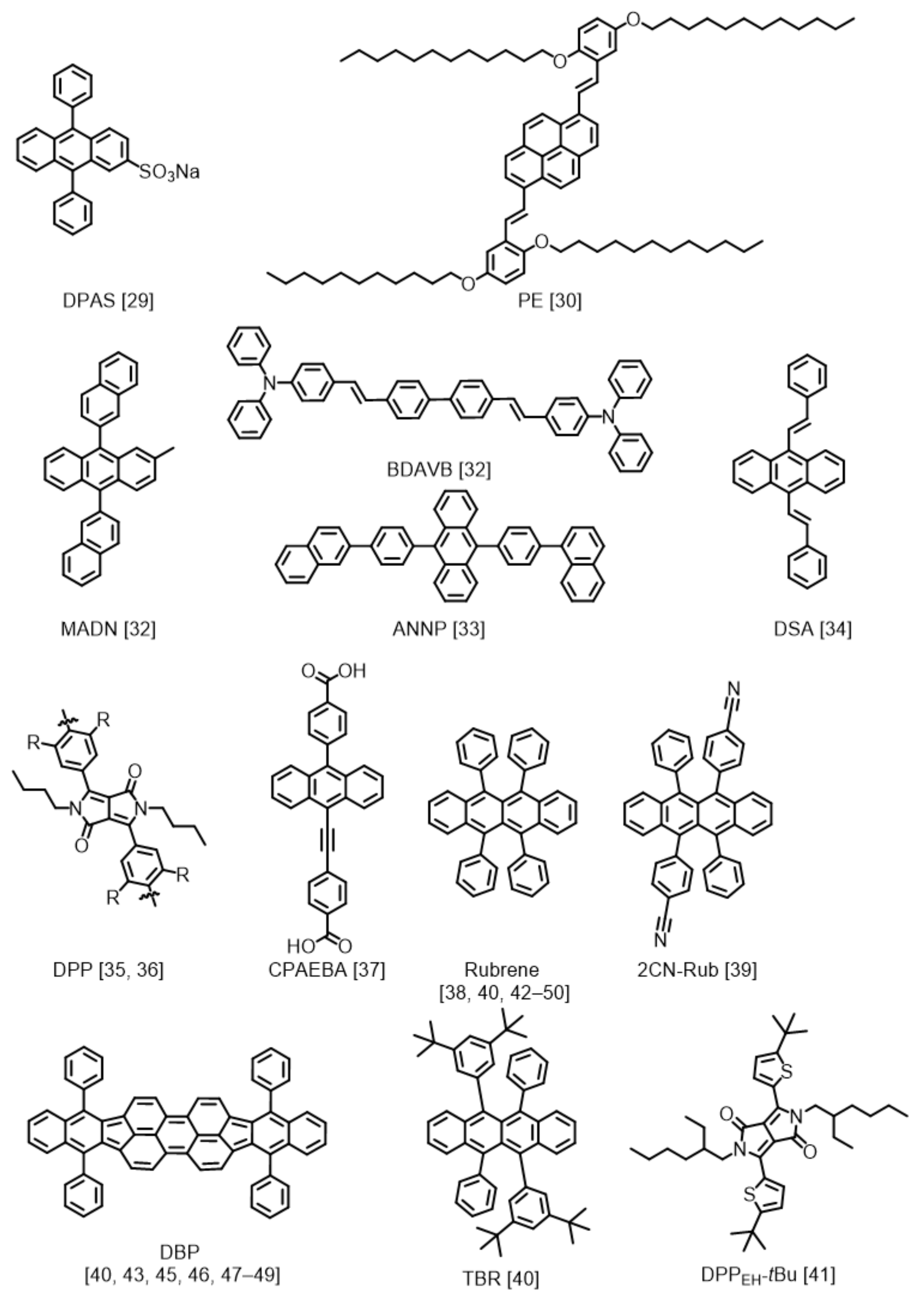

Figure R5. Chemical structures of molecular triplet acceptors from Ref. 29 to 50.

Table R2. Summary of energy migration based solid-state TTA-UC performances reported to date[a,b].

Excitation wavelength (nm)	UC emission wavelength (nm)	Donor[c]	Acceptor[c]	Φ_{UC}[d] (%)	I_{th} (mW cm^{-2})	Ref.
440	390–393	CBDAC	PPO	4.3	13.8	16
445	394	BBCz-SB-Br	SiDPN-2	1.3	44	17
530	435	PtOEP	DPA	3	5.6	18
532	427	PtOEP	bDPA-2	0.92	52	19
532	427	PtOEP	bDPA-1	0.31	110	19
532	435	PdMesoP	$(DCA)_2ADC$	3	49	20
532	436	PtOEP	DPA	0.022	200	19
532	440	PtOEP	A1	0.14	2.5	21
532	440	PtOEP	A2	0.1	1.7	21
532	440	PtOEP	DPA	2.5	6	22
532	440	PtOEP	C7-sDPA	5–10	5	23
532	440	PtOEP	DPA	0.022	450	24
532	440	PtOEP	DPA	1.0	531	24
532	440	PtOEP	DPA	0.22	326	24
532	440	Pd-TCPP	adba	0.64	2.5	25
532	440	PtOEP	A2/TTBP	4.5	195	26
532	440	PtOEP	Epo-DPA/ P_{66614}DPAS	5.7	39.1	27
532	440	PtOEP	DPA	8	5	28
532	445	PtOEP	DPAS	23.8	31.7	29
532	445	PtOEP	DPA/PE	2.7	39	30
532	450	CdSe/CdS	adba	0.00045	1000	31
532	470	PtOEP	A3	0.008	10.5	21
532	400–500	PtOEP	MADN/ BDAVBi	1.23	238	32
542	434	PtOEP	ANNP	13.4	2.1	33
640	514	PdTPTBP	DSA	0.29	35.9	34
721	573	PdTNP	DPP-PAF-2	1.5 (corrected)	1100	35
721	573	tBu-cy5	DPP-COF-2	7.6 (corrected)	980	36
724	540	$Os(tpyCOOH)_2^{2+}$	CPAEBA	0.006 (corrected)	10000	37
730	560	PdPc	Rubrene	1.2	1400	38
730	580	PdPc	2CN-Rub	3.0	3300	39

730	610	PdPc	Rubrene/DBP	0.07	2100	40
730	610	PdPc	TBR/DBP	0.3	2400	40
730	635	PdPc	DPP_{EH}-*t*Bu	1.06	16200	41
750	565	ITIC-Cl	Rubrene	1.42	39	42
772	610	$MoSe_2$	Rubrene/DBP	1.1	260	43
785	570	PdTPTAP	Rubrene	0.25	115	44
785	610	$MA_{0.15}FA_{0.85}PbI_3$	Rubrene/DBP	1.55	500	45
785	610	$MA_{0.15}FA_{0.85}PbI_3$	Rubrene/DBP	0.2445	61	46
808	573	PYIT1/PBQx-TCl	Rubrene	0.85	8	47
808	608	PYIT1/PBQx-TCl	Rubrene/DBP	2.2	10	47
808	610	Y6	Rubrene/DBP	0.0268	68.2	48
808	610	Y6	Rubrene/DBP	0.0179	92.5	48
808	612	PbS	Rubrene/DBP	0.26	17	49
808	612	PbS	Rubrene/DBP	0.62	12	49
808	612	PbS	Rubrene/DBP	0.11	26	49
850	565	Y6	Rubrene	0.515	19	42
938	580	Os(atpy)(tbbpy)Cl^+	Rubrene	1.55	10000	50

[a] Only reports documenting I_{th} and Φ_{UC} values are included in the summary.

[b] Some values were estimated from figures in the literature.

[c] Chemical structures are shown in Figure S33.

[d] The maximum value of Φ_{UC} is 50%.

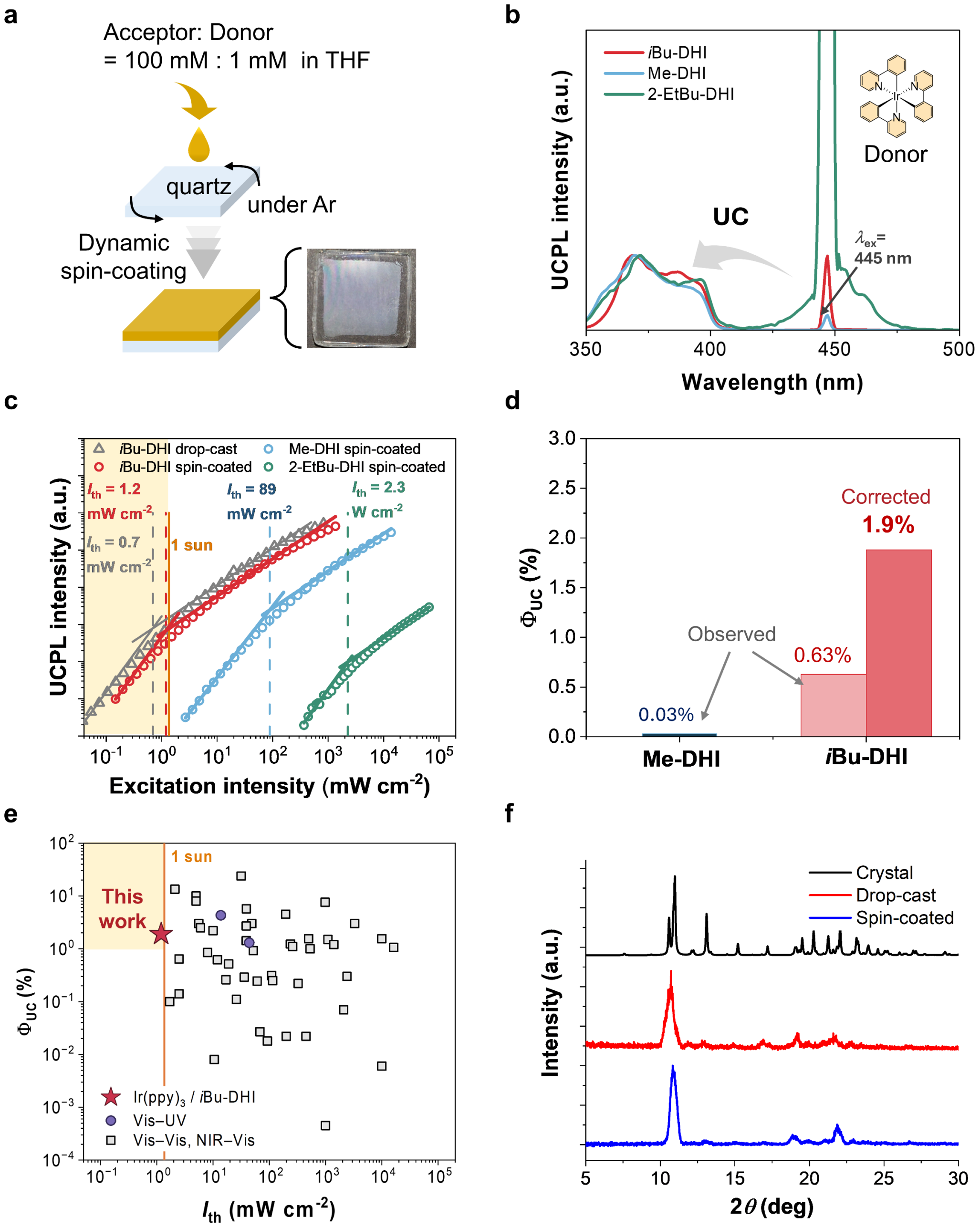

Figure R6. a Fabrication procedure of UC solid films by dynamic disperse spin-coating, **b** UC emission spectra, **c** Excitation intensity dependence of the upconverted emission intensity around 370 nm, and **d** absolute Φ_{UC} in solid of Me-DHI (blue), *i*Bu-DHI (red), and 2-EtBu-DHI (green) paired with Ir(ppy)$_3$ (λ_{ex} = 445 nm, short-pass filter: 400 nm, molar ratio for preparation; acceptor: donor = 100: 1). The gray plots in Figure 3c show the data of *i*Bu-DHI/Ir(ppy)$_3$ prepared by the drop-casting method. **e** Φ_{UC}–I_{th} plots with the reported values of solid TTA-UC systems. Details of the references for the plot are provided in the supporting information (Figure S30 and Table S8). **f** Comparison of XRD patterns of crystalline solid of *i*Bu-DHI (black) and solid samples of *i*Bu-DHI with Ir(ppy)$_3$ prepared by spin-coating (blue) and drop-casting (red) methods.

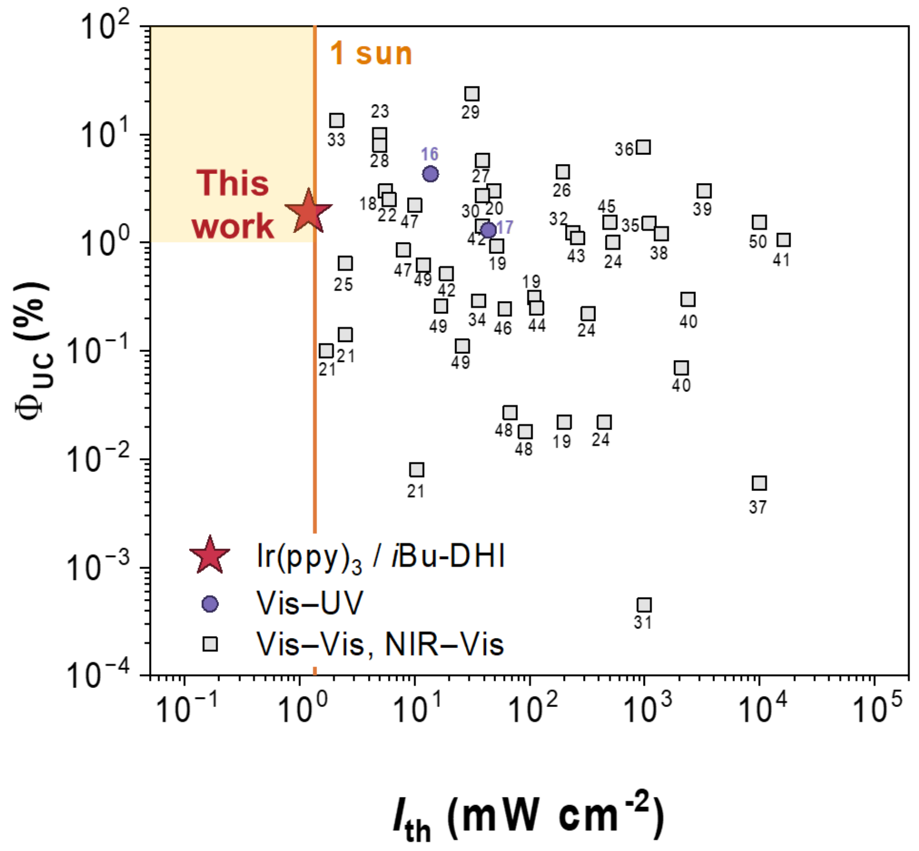

Figure R7. Φ_{UC}–I_{th} plots with the reported values of solid TTA-UC systems with reference numbers.

Comments from Reviewer 3

I thank the authors for their work and changes to the manuscript. I feel my concerns have been well addressed, though I strong encourage the authors to include the information (particularly the aggregation work/R20) in the manuscript or supporting information, as it could be quite helpful for follow-on studies.

I highlight that I found the other reviewers' comments to be quite reasonable, and therefore they should be fully resolved before publication.

We appreciate the reviewer's positive comments.

Following the reviewer's suggestion, we have added a discussion of the aggregation of 2-EtBu-DHI in the revised manuscript (Figures R8 and, R9).

Page 7, line 21

"The 2-EtBu-DHI UC emission was much weaker due to aggregation (section 7 in Supplementary Information)."

Page S17, line 1

"Irradiation at 445 nm to the mixed solution of $Ir(ppy)_3$ and 2-EtBu-DHI resulted in the formation of aggregates. This appears to be the result of an interaction between $Ir(ppy)_3$ and 2-EtBu-DHI as aggregates were not observed upon UV irradiation of the concentrated 2-EtBu-DHI solution. To investigate the interaction between $Ir(ppy)_3$ and 2-EtBu-DHI in the ground state, ^{1}H-NMR spectroscopy was conducted on mixtures of 2-EtBu-DHI and $Ir(ppy)_3$ at molar ratios of 10:1, 20:1, and 100:1 (Figure S14). The 20:1 and 100:1 conditions are identical to those used for the UC measurements (Figure S9-11). The ^{1}H-NMR spectra of all samples showed the same pattern as 2-Et-Bu-DHI, suggesting that aggregation-induced species are not present. After laser exposure, a white precipitate formed in the cuvette (Figure S15b), indicating that the aggregation is a photo-induced event that occurs exclusively during laser excitation.

To evaluate whether aggregation occurs in other solvents, the sample in toluene was also tested (Figure S15a). Similar aggregation was observed in the toluene solution, suggesting that the interaction between $Ir(ppy)_3$ and 2-EtBu-DHI is not limited to a specific solvent environment. This indicates that the aggregation behavior is likely driven by intrinsic molecular interactions rather than by solvent-specific effects. However, the mechanism remains to be determined. Further investigation is required to clarify the origin of this phenomenon."

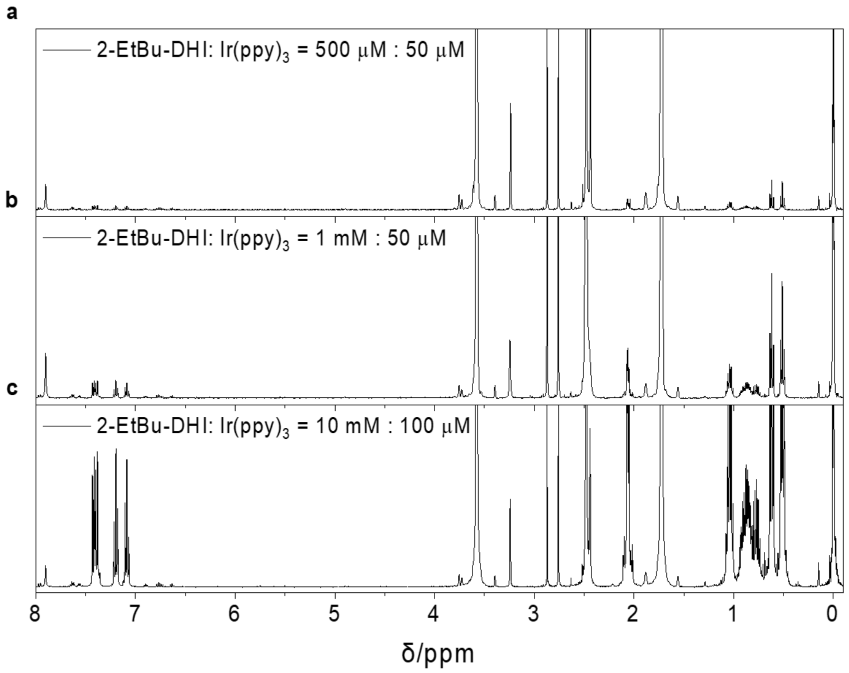

Figure R8. ^{1}H-NMR (400 MHz, THF-*D8*, TMS) spectra of the **a** 500 μM: 50 μM mixture, **b** 1 mM: 50 μM mixture, and **c** 10 mM: 100 μM mixture of 2-EtBu-DHI and $Ir(ppy)_3$.

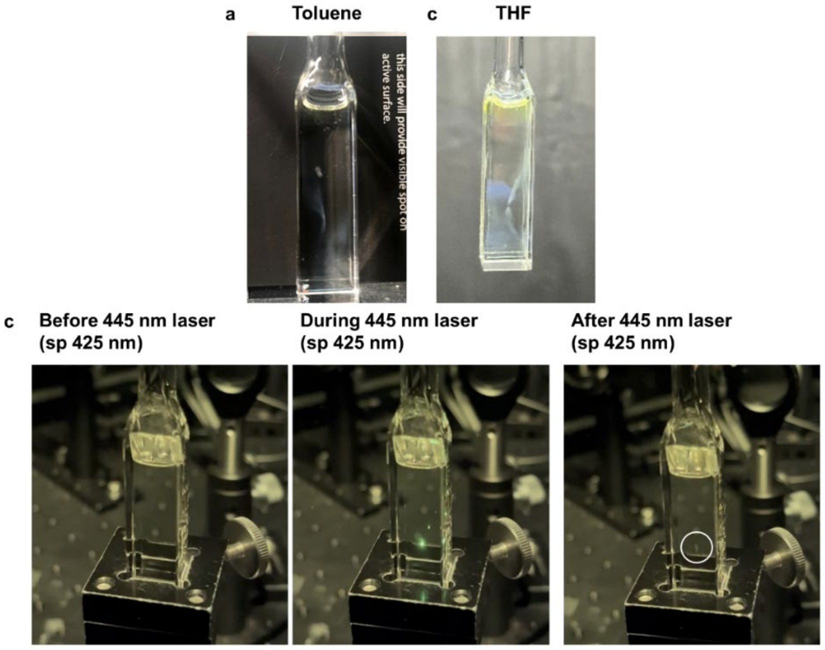

Figure R9. Photographs illustrating the influence of solvent environment on the formation of molecular aggregates in **a** toluene and **b** THF. **c** Solvent-dependent aggregation behavior and photo-induced assembly of 2-EtBu-DHI with $Ir(ppy)_3$. The concentrations of 2-EtBu-DHI and $Ir(ppy)_3$ were 10 mM and 100 μM, respectively.
